# Supplementary material for: Timing matters: Exon skipping therapy is most effective when initiated early in a mouse model of Duchenne muscular dystrophy
Source: Mol Ther Nucleic Acids. 2026 Jul 17;37(3):103017. doi: 10.1016/j.omtn.2026.103017 (PMC13448210; doi:10.1016/j.omtn.2026.103017)
Supplement: Supplementary file 1 [file mmc1.pdf]

OMTN, Volume *37*

Supplemental information

Timing matters: Exon skipping therapy is most effective when initiated early in a mouse model of Duchenne muscular dystrophy

Sofia Stenler, Junyu Huang, Tirsa L.E. van Westering, Anna M.L. Coenen-Stass, Yahya Jad, Kaarel Krjutškov, Samir EL Andaloussi, Matthew J.A. Wood, and Thomas C. Roberts

Supplemental Information

Table S1

RT-qPCR and RT-ddPCR assays used in this study.

All sequence are 5′ to 3′.

		**Sequence**
***Dmd* exon 20-21**	**FWD**	AGATGACAACTACTGCCGAA
	**REV**	GAAGAGCTGACAATCTGTTGAC
	**PROBE**	AGTCTACCACCCTATCAGAGCCAACA
***Dmd* exon 23-24**	**FWD**	GAAACTTTCCTCCCAGTTGGT
	**REV**	CAGGCCATTCCTCTTTCAGG
	**PROBE**	TCAACTTCAGCCATCCATTTCTGTAAGGT
***Dmd* exon 1-2**	**FWD**	AGCCTCCTGGAGCAATAAAA
	**REV**	AATTGTGCATTTATCCATTTTGTGA
***Dmd* exon 44-45**	**FWD**	GCGATTCGACAGATCAGTTG
	**REV**	CCTTTCTGCCAGCTCTTTGC
***Dmd* exon 63-64**	**FWD**	CCACGAGACCCAAACCACT
	**REV**	GGGCCTTCTGGAGCCTTCT

Table S2

List of Small RNA TaqMan RT-qPCR assays used in this study.

All assays were obtained from Thermo Fisher Scientific.

Target	Product ID
mmu-miR-16-5p	000391
mmu-miR-31-5p	000185
mmu-miR-34c-5p	000428
mmu-miR-206-3p	000510

Table S3

Summary of RNA-Seq counts data and differential expression analysis.

Spreadsheet containing DESeq2 outputs including normalized counts data and pairwise differential expression analyses.

A

		Total RNA				rRNA minus
	Sample ID	ng/µl	A260/A280	A260/A230	RIN	ng/µl
Adult (14 weeks)	C57 1	77.2	2.11	1.85	9.6	16
	C57 2	72.4	2.09	1.79	8.4	10
	C57 3	63.9	2.08	1.77	9.2	9
	mdx 1	154.3	2.07	1.97	7.8	8.8
	mdx 2	65.7	2.03	1.71	6.7	8.7
	mdx 3	84.7	2.08	1.73	7.6	15
	PPMO 1	73.3	2.06	1.79	9.4	11
	PPMO 2	76	2.05	1.62	9.2	13
	PPMO 3	78.3	2.07	1.79	9.2	9.8
Aged (78-80 weeks)	C57 1	62.2	2.09	1.77	9.2	15
	C57 2	45.4	2.01	1.58	8.9	12
	C57 3	46.9	2.07	1.71	8.7	7.4
	mdx 1	70.5	2.06	1.77	8.1	11
	mdx 2	61	2.08	1.82	7.9	14
	mdx 3	51.9	2.07	1.72	8.4	14
	PPMO 1	60.8	2.03	1.53	8.7	12
	PPMO 2	46.5	2.01	1.63	8.4	7.5
	PPMO 3	77.5	2.06	1.71	9	4.8

B

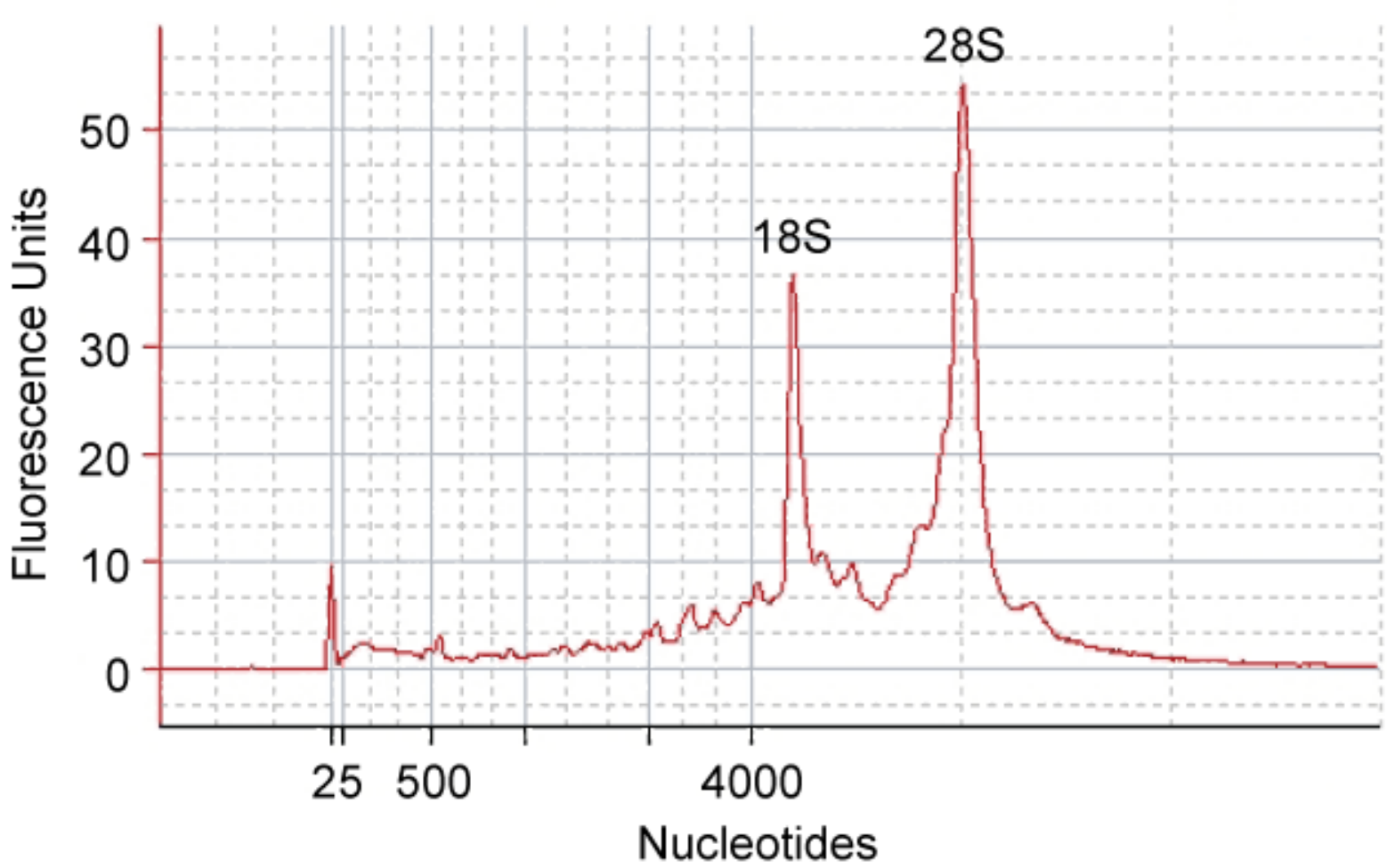

C

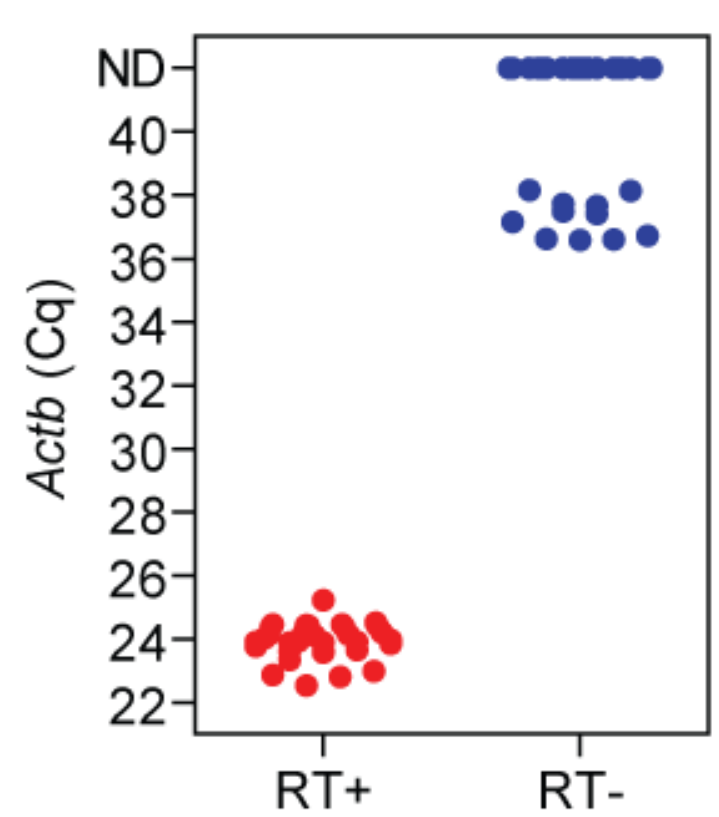

Figure S1

RNA-Seq quality control analyses.

(**A**) Summary of sample assessments of RNA concentration, quality, and integrity by UV spectrophotometry, bioanalyzer, and fluorimetry (post rRNA depletion). (**B**) Typical bioanalyzer trace indicating intact RNA samples with prominent rRNA peaks (pre rRNA removal). (**C**) RNA samples were assessed by genomic DNA contamination by measurement of *Actb* mRNA expression by RT-qPCR. This assay is designed in such a way that it has the potential to amplify both mRNA and its encoding DNA. Amplification in a RT- control sample would therefore be indicative of genomic DNA contamination. *Actb* levels in the RT- samples were either note detected (ND) or in the single copy range (Ct >35).

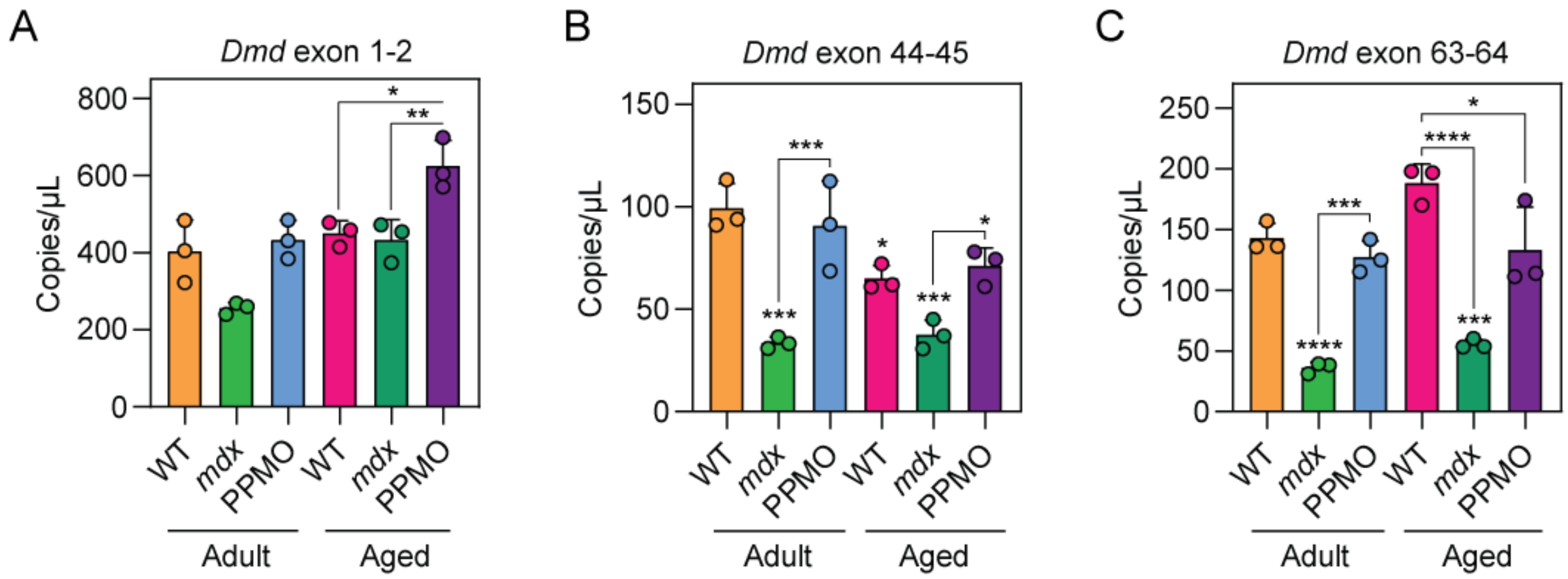

Figure S2

Analysis of *Dmd* transcript imbalance phenomena in Adult and Aged *mdx* mice.

RNA samples from WT, *mdx*, and PPMO-treated *mdx* mice at Adult and Aged time points were analysed by RT-ddPCR to determine absolute quantification of *Dmd* transcripts targeting regions (**A**) exon 1-2 (**B**) exon 44-45, and (**C**) exon 63-63. All values are mean+SD (*n*=3). Statistical significance was tested by one-way ANOVA with Tukey's *post hoc test*. Statistical comparisons are relative to the Adult WT group unless otherwise indicated, **P*<0.05, ***P*<0.01, ****P*<0.001, *****P*<0.0001.

A

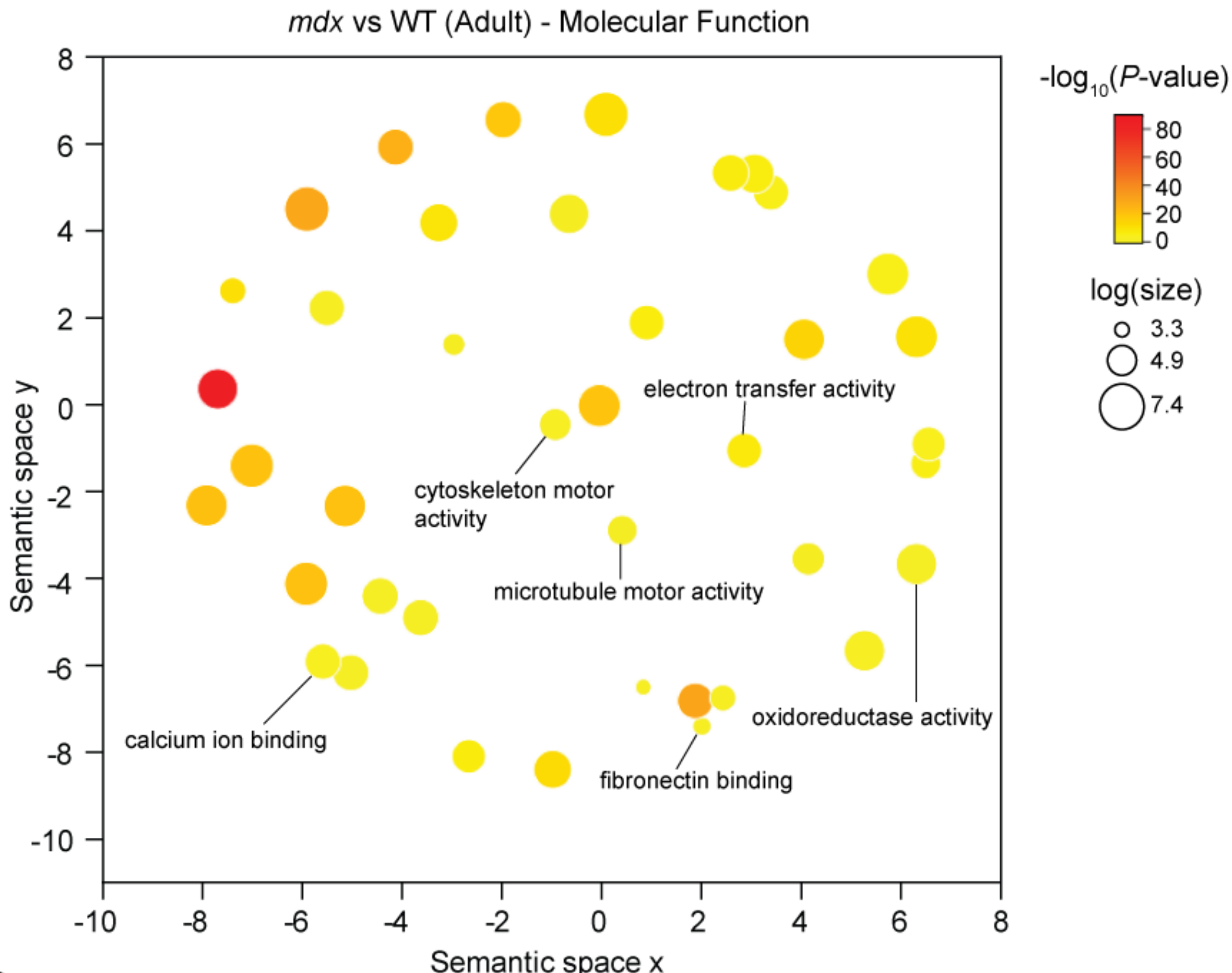

mdx vs WT (Adult) - Molecular Function
-log10(P-value)
80
60
40
20
0
log(size)
3.3
4.9
7.4
Semantic space y
Semantic space x
electron transfer activity
cytoskeleton motor activity
microtubule motor activity
calcium ion binding
oxidoreductase activity
fibronectin binding

B

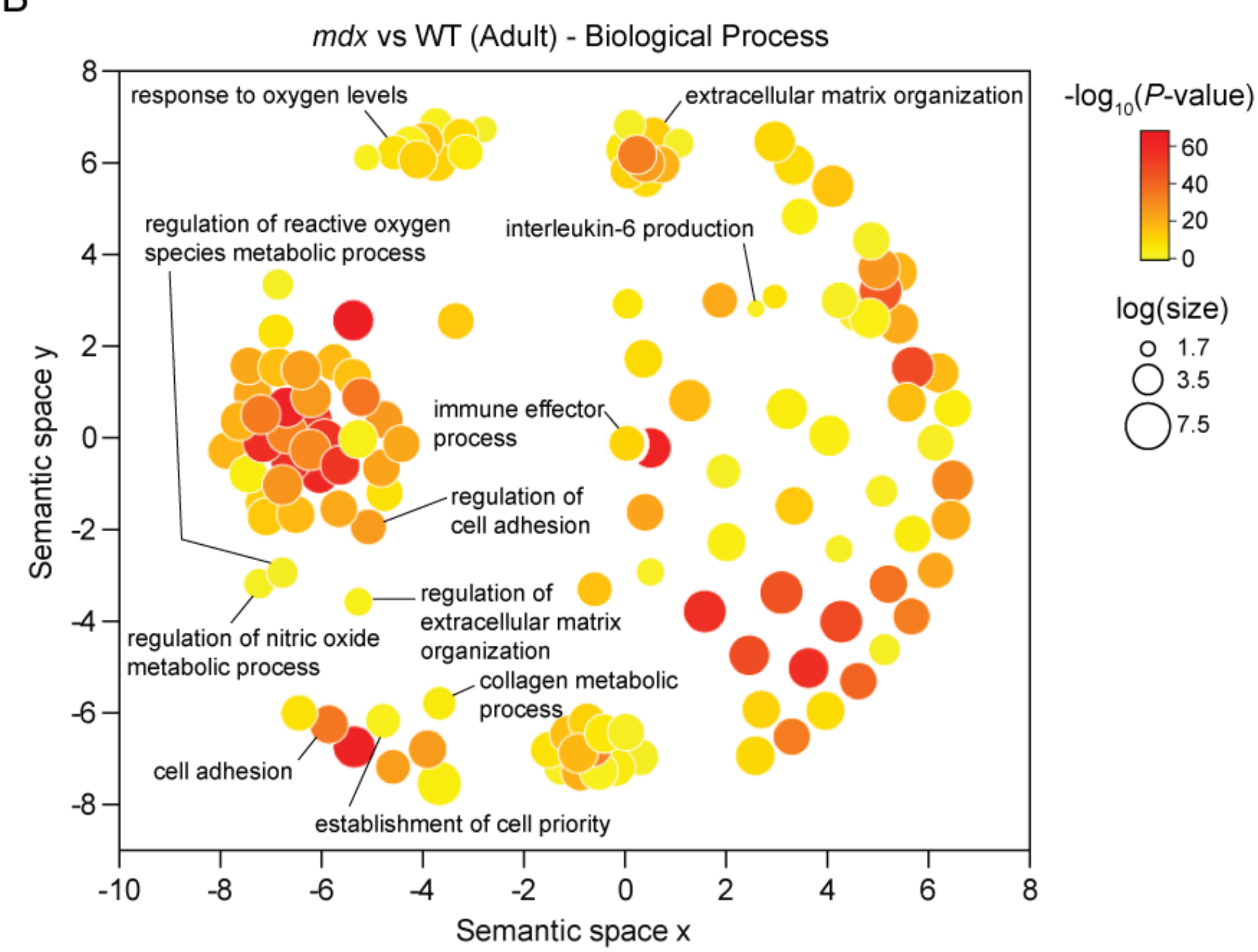

mdx vs WT (Adult) - Biological Process
-log10(P-value)
60
40
20
0
log(size)
1.7
3.5
7.5
Semantic space y
Semantic space x
response to oxygen levels
extracellular matrix organization
regulation of reactive oxygen species metabolic process
interleukin-6 production
immune effector process
regulation of cell adhesion
regulation of extracellular matrix organization
regulation of nitric oxide metabolic process
collagen metabolic process
cell adhesion
establishment of cell priority

Figure S3

Gene ontology analysis for differentially expressed genes in Adult *mdx* vs WT.

Differentially-expressed genes from RNA-Seq data (adjusted $P<0.01$) for the Adult *mdx* vs WT libraries were analysed for enriched gene ontology (GO) terms using g:Profiler and the statistically significant (adjusted $P<0.05$) terms visualized using REVIGO to reduce redundancy and improve interpretability for (**A**) molecular function, and (**B**) biological process. Circle size indicates the frequency of the gene ontology annotation term in the underlying database, and colour indicates the statistical significance of the enrichment. Distances between circles reflect semantic similarity among GO terms.

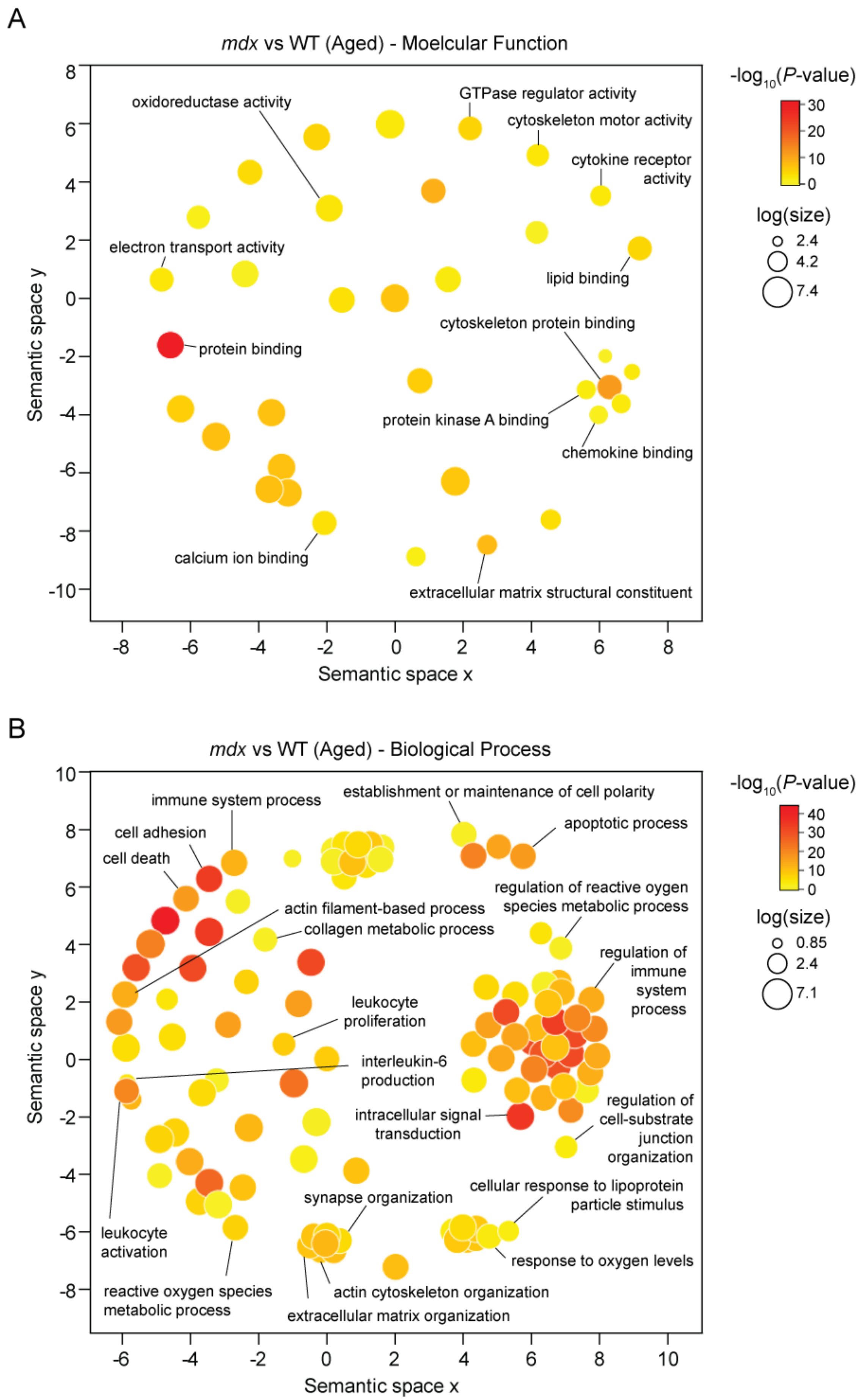
A
mdx vs WT (Aged) - Moelcular Function
oxidoreductase activity
GTPase regulator activity
cytoskeleton motor activity
cytokine receptor activity
electron transport activity
lipid binding
cytoskeleton protein binding
protein binding
protein kinase A binding
chemokine binding
calcium ion binding
extracellular matrix structural constituent
Semantic space x
Semantic space y
-log10(P-value)
log(size)
2.4
4.2
7.4
B
mdx vs WT (Aged) - Biological Process
immune system process
establishment or maintenance of cell polarity
cell adhesion
apoptotic process
cell death
regulation of reactive oxygen species metabolic process
actin filament-based process
collagen metabolic process
regulation of immune system process
leukocyte proliferation
interleukin-6 production
intracellular signal transduction
regulation of cell-substrate junction organization
synapse organization
cellular response to lipoprotein particle stimulus
leukocyte activation
response to oxygen levels
reactive oxygen species metabolic process
actin cytoskeleton organization
extracellular matrix organization
Semantic space x
Semantic space y
-log10(P-value)
log(size)
0.85
2.4
7.1

Figure S4

Gene ontology analysis for differentially expressed genes in Aged *mdx* vs WT.

Differentially-expressed genes from RNA-Seq data (adjusted *P*<0.01) for the Aged *mdx* vs WT libraries were analysed for enriched gene ontology (GO) terms using g:Profiler and the statistically significant (adjusted *P*<0.05) terms visualized using REVIGO to reduce redundancy and improve interpretability for (**A**) molecular function, and (**B**) biological process. Circle size indicates the frequency of the gene ontology annotation term in the underlying database, and colour indicates the statistical significance of the enrichment. Distances between circles reflect semantic similarity among GO terms.

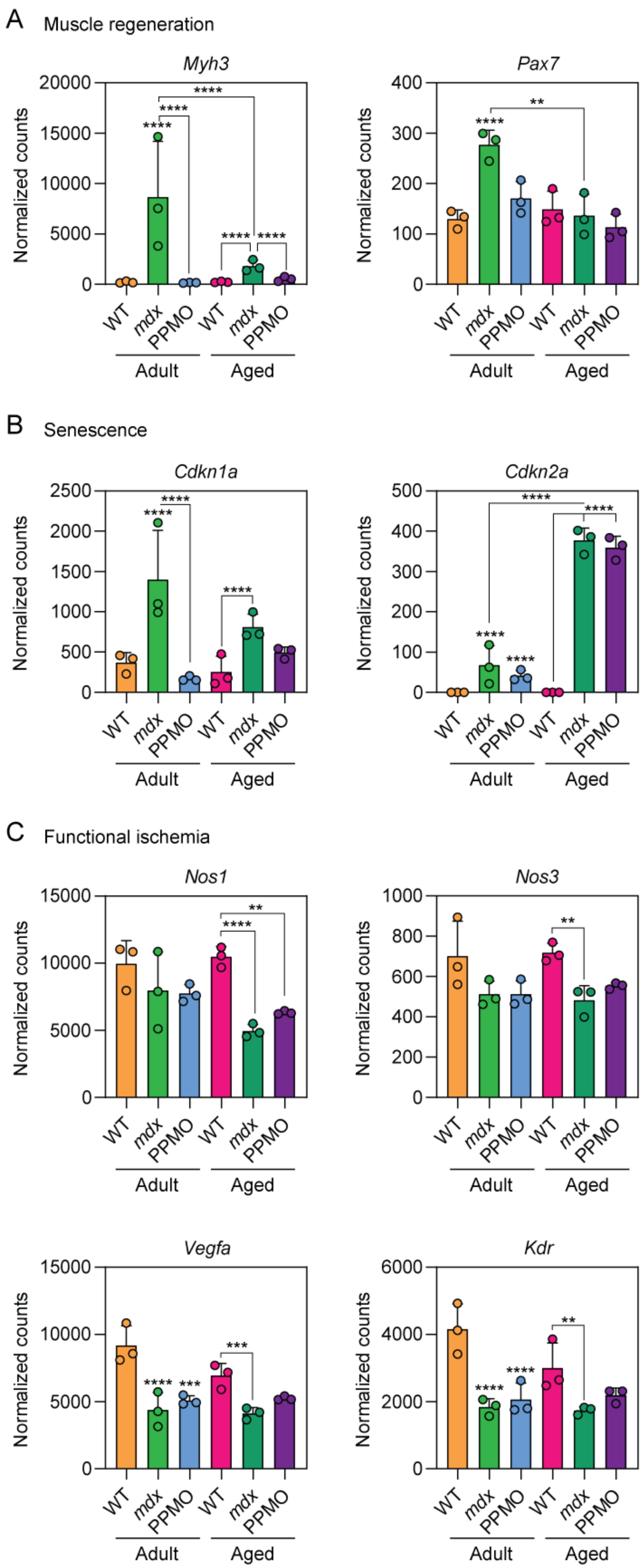
A Muscle regeneration
Myh3
Pax7
B Senescence
Cdkn1a
Cdkn2a
C Functional ischemia
Nos1
Nos3
Vegfa
Kdr
Normalized counts
WT
mdx
PPMO
Adult
Aged

Figure S5

Differential expression of muscle regeneration, senescence, and functional ischemia markers in dystrophic muscle.

RNA-Seq normalized counts data for marker genes associated with (**A**) muscle regeneration, (**B**) senescence, and (**C**) functional ischemia. Values are mean+SD. Statistical significance was assessed using DESeq2 and Benjamini-Hochberg-adjusted *P*-values indicated. Statistical comparisons are relative to the Adult WT group unless otherwise indicated, $^{**}P<0.01$, $^{***}P<0.001$, $^{****}P<0.0001$.

Handling of reactive oxygen species

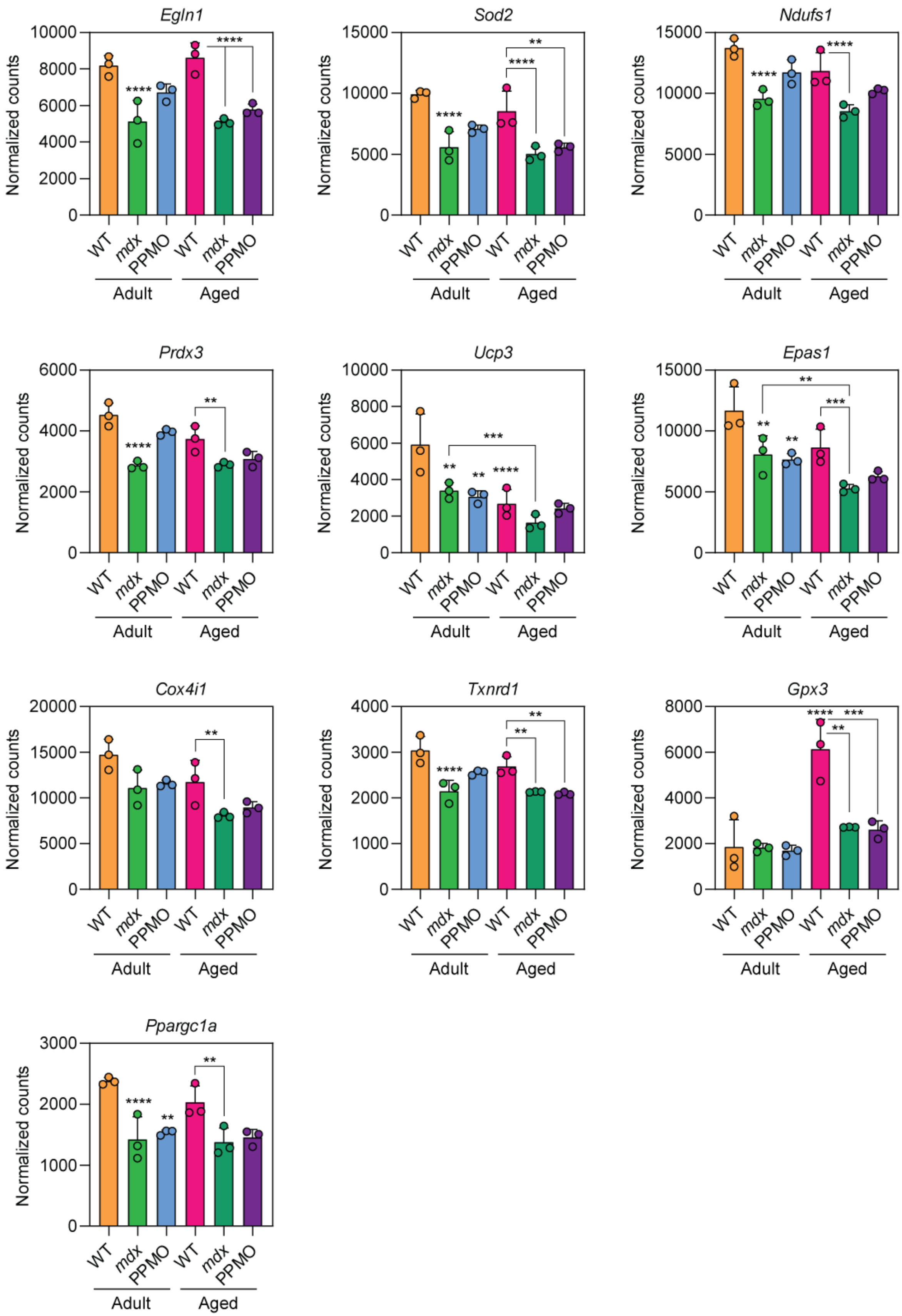

Figure S6

Differential expression of reactive oxygen species handling markers in dystrophic muscle.

RNA-Seq normalized counts data for marker genes associated with handling of reactive oxygen species. Values are mean+SD. Statistical significance was assessed using DESeq2 and Benjamini-Hochberg-adjusted *P*-values indicated. Statistical comparisons are relative to the Adult WT group unless otherwise indicated, $^{**}P<0.01$, $^{***}P<0.001$, $^{****}P<0.0001$.

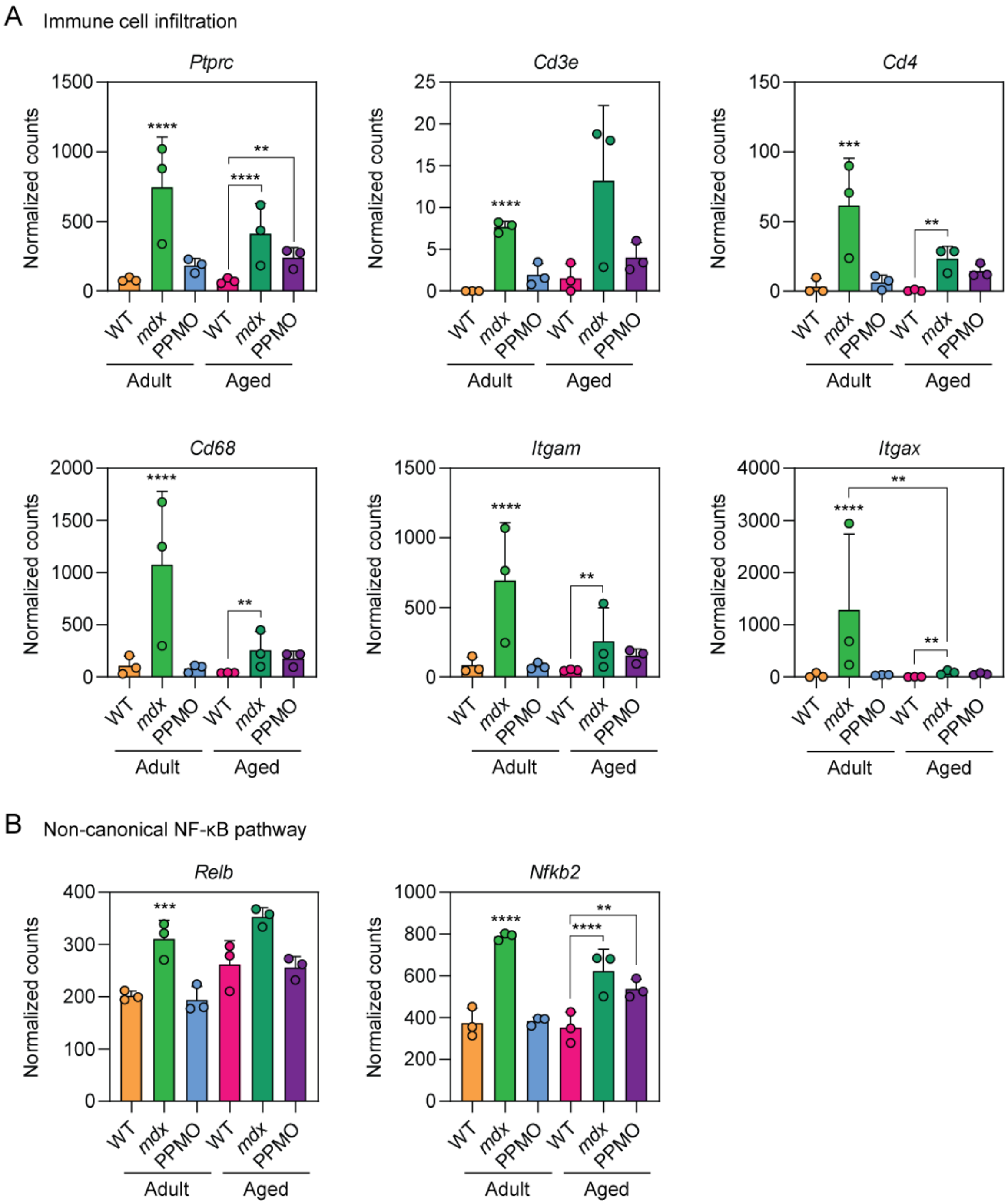
A
Immune cell infiltration
Ptprc
Cd3e
Cd4
Cd68
Itgam
Itgax
B
Non-canonical NF-κB pathway
Relb
Nfkb2
Normalized counts
WT
mdx
PPMO
Adult
Aged
****
***
**

Figure S7

Differential expression of immune infiltration and NF-κB pathway markers in dystrophic muscle.

RNA-Seq normalized counts data for marker genes associated with (**A**) immune cell infiltration and (**B**) the non-canonical NF-κB pathway. Values are mean+SD. Statistical significance was assessed using DESeq2 and Benjamini-Hochberg-adjusted *P*-values indicated. Statistical comparisons are relative to the Adult WT group unless otherwise indicated, $^{**}P<0.01$, $^{***}P<0.001$, $^{****}P<0.0001$.

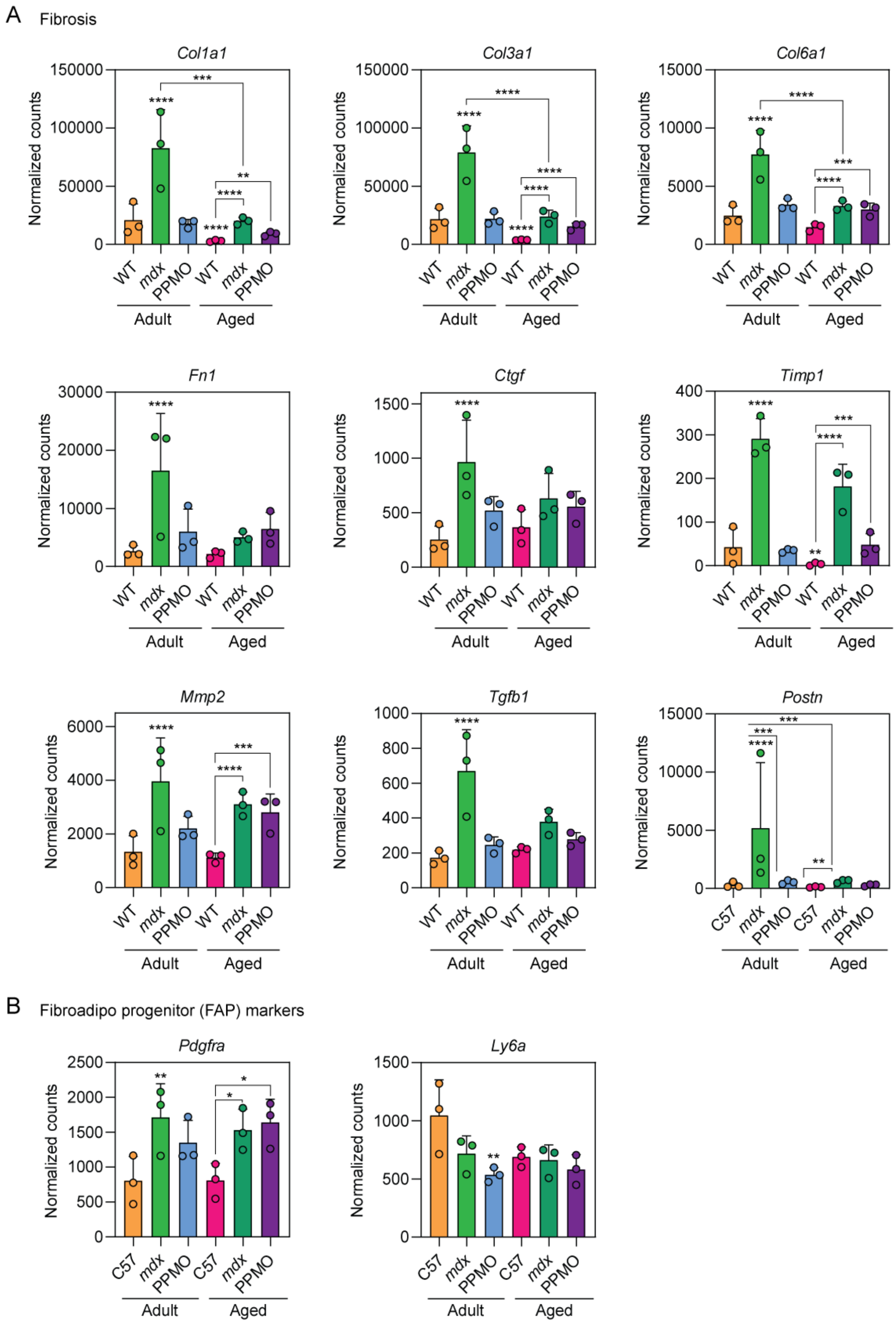
A Fibrosis
Col1a1
Col3a1
Col6a1
Fn1
Ctgf
Timp1
Mmp2
Tgfb1
Postn
Normalized counts
WT
mdx
PPMO
C57
Adult
Aged
B Fibroadipo progenitor (FAP) markers
Pdgfra
Ly6a

Figure S8

Differential expression of fibrosis markers in dystrophic muscle.

RNA-Seq normalized counts data for marker genes associated with (**A**) fibrosis, and (**B**) fibroadipo progenitor (FAP) markers. Values are mean+SD. Statistical significance was assessed using DESeq2 and Benjamini-Hochberg-adjusted *P*-values indicated. Statistical comparisons are relative to the Adult WT group unless otherwise indicated, *$P<0.05$, **$P<0.01$, ***$P<0.001$, ****$P<0.0001$.

AKT/PI3K/mTOR axis

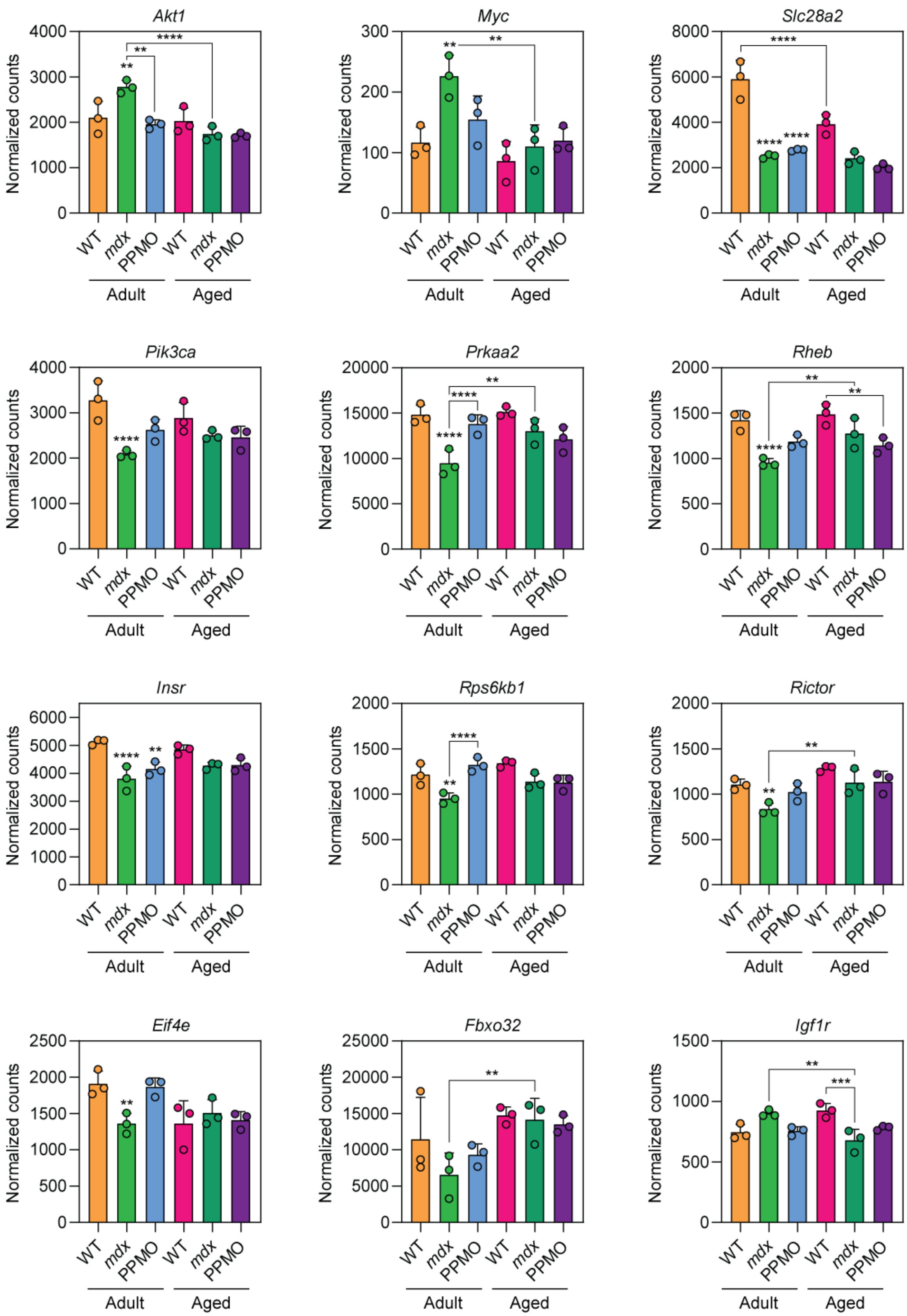

Figure S9

Differential expression of AKT/PI3K/mTOR axis markers in dystrophic muscle.

RNA-Seq normalized counts data for marker genes associated with the AKT/PI3K/mTOR axis. Values are mean+SD. Statistical significance was assessed using DESeq2 and Benjamini-Hochberg-adjusted *P*-values indicated. Statistical comparisons are relative to the Adult WT group unless otherwise indicated, $^{**}P<0.01$, $^{***}P<0.001$, $^{****}P<0.0001$.

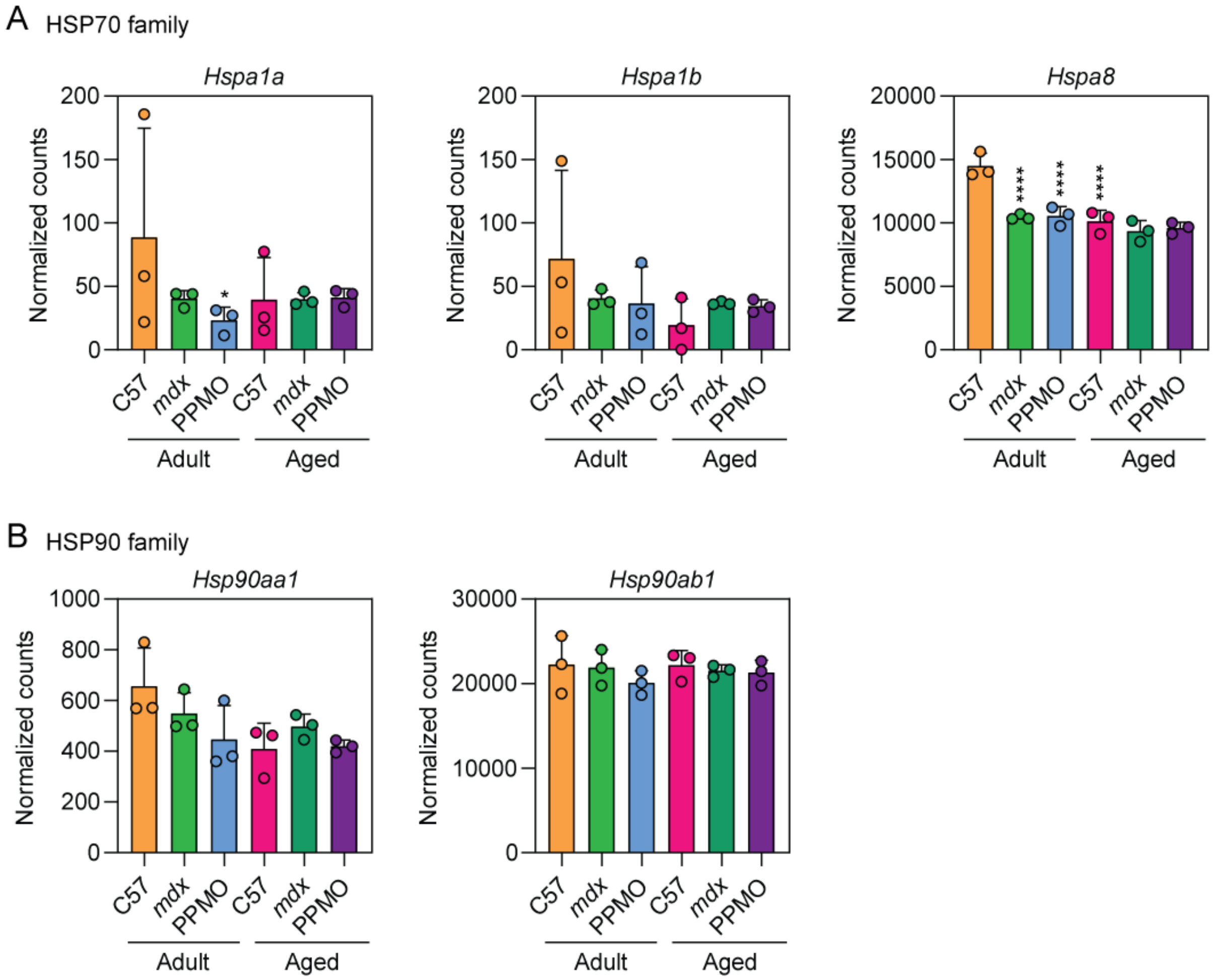
A HSP70 family
Hspa1a
Hspa1b
Hspa8
Normalized counts
C57
mdx
PPMO
Adult
Aged
B HSP90 family
Hsp90aa1
Hsp90ab1

Figure S10

Differential expression of molecular chaperones in dystrophic muscle.

RNA-Seq normalized counts data for marker genes associated with molecular chaperons from the (**A**) HSP70 family, and (**B**) HSP90 family. Values are mean+SD. Statistical significance was assessed using DESeq2 and Benjamini-Hochberg-adjusted *P*-values indicated. Statistical comparisons are relative to the Adult WT group unless otherwise indicated, $^{**}P<0.01$, $^{****}P<0.0001$.

Ubiquitin-proteasome system

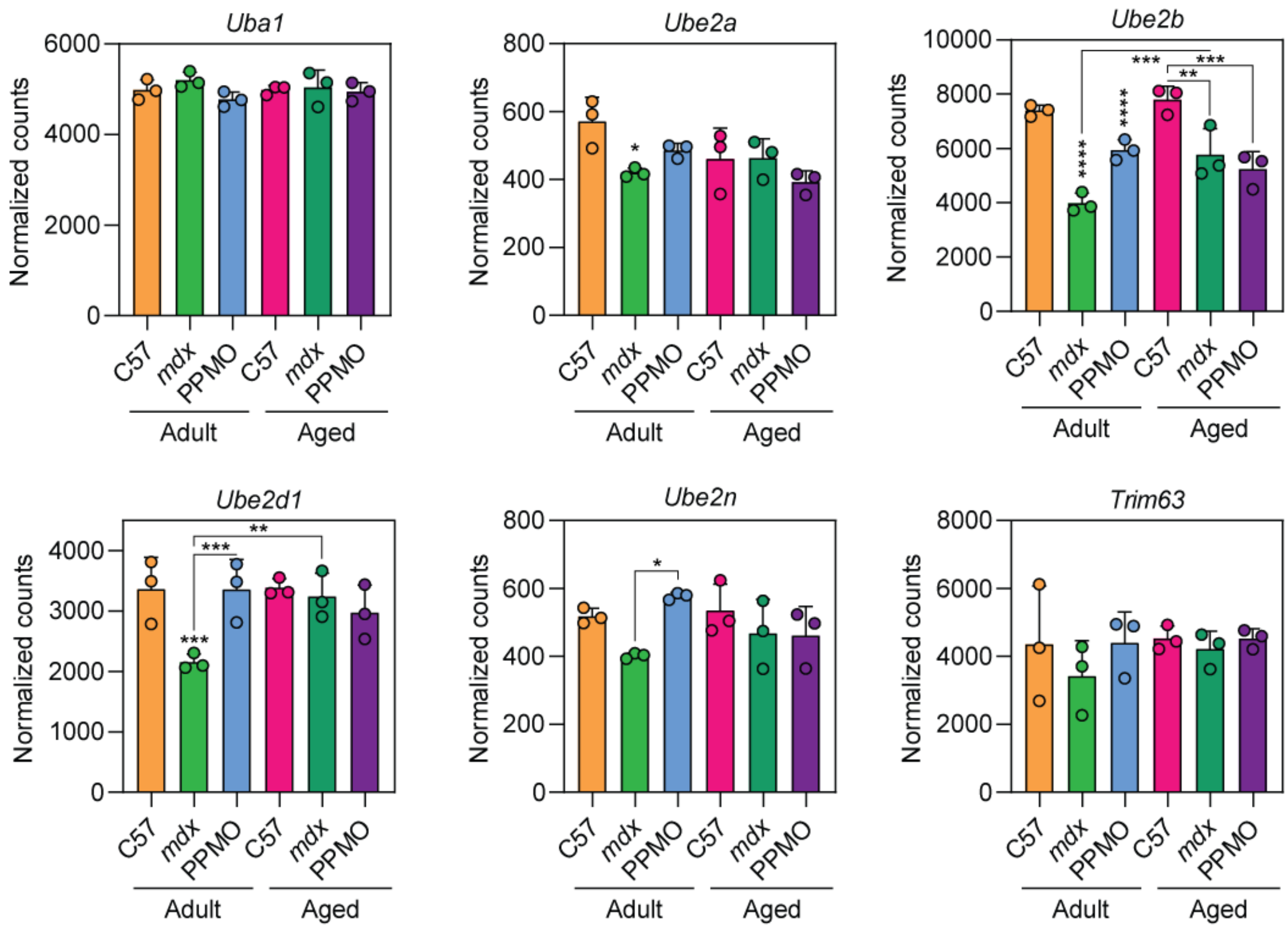

Figure S11

Differential expression of components of the ubiquitin-proteasome system in dystrophic muscle.

RNA-Seq normalized counts data for marker genes associated with components of the ubiquitin-proteasome system. Values are mean+SD. Statistical significance was assessed using DESeq2 and Benjamini-Hochberg-adjusted *P*-values indicated. Statistical comparisons are relative to the Adult WT group unless otherwise indicated, $^{*}P<0.05$, $^{**}P<0.01$, $^{***}P<0.001$, $^{****}P<0.0001$.

Autophagy

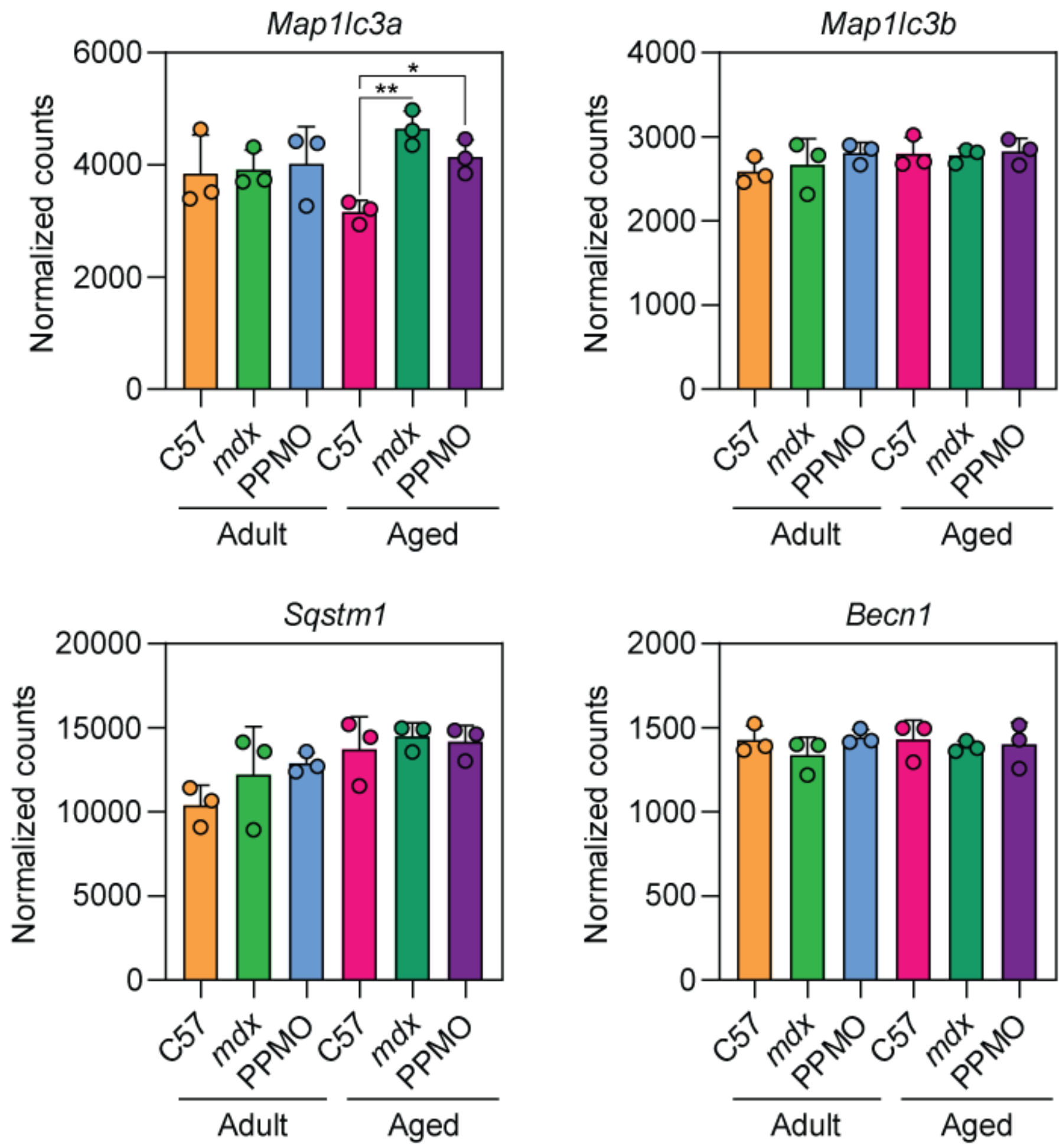

Map1lc3a
Normalized counts
6000
4000
2000
0
**
*
C57
mdx
PPMO
C57
mdx
PPMO
Adult
Aged
Map1lc3b
Normalized counts
4000
3000
2000
1000
0
C57
mdx
PPMO
C57
mdx
PPMO
Adult
Aged
Sqstm1
Normalized counts
20000
15000
10000
5000
0
C57
mdx
PPMO
C57
mdx
PPMO
Adult
Aged
Becn1
Normalized counts
2000
1500
1000
500
0
C57
mdx
PPMO
C57
mdx
PPMO
Adult
Aged

Figure S12

Differential expression autophagy associated genes in dystrophic muscle.

RNA-Seq normalized counts data for marker genes associated with autophagy. Values are mean+SD. Statistical significance was assessed using DESeq2 and Benjamini-Hochberg-adjusted *P*-values indicated. *$P<0.05$, **$P<0.01$.
