# Supplementary material for: Timing matters: Exon skipping therapy is most effective when initiated early in a mouse model of Duchenne muscular dystrophy
Source: Mol Ther Nucleic Acids. 2026 Jul 17;37(3):103017. doi: 10.1016/j.omtn.2026.103017 (PMC13448210; doi:10.1016/j.omtn.2026.103017)
Supplement: Supplementary file 2 [file mmc3.pdf]

Molecular Therapy
Nucleic Acids
Original Article

Timing matters: Exon skipping therapy is most effective when initiated early in a mouse model of Duchenne muscular dystrophy

Sofia Stenler,[1,2] Junyu Huang,[3,4] Tirsa L.E. van Westering,[1,5] Anna M.L. Coenen-Stass,[1,6] Yahya Jad,[3,4] Kaarel Krjutškov,[7,8] Samir EL Andaloussi,[9] Matthew J.A. Wood,[1,3,4,10] and Thomas C. Roberts[1,3,4,10]

[1]Department of Physiology, Anatomy and Genetics, University of Oxford, South Parks Road, Oxford OX1 3QX, UK; [2]Uppsala University, Office for Science and Technology, 751 05 Uppsala, Sweden; [3]Department of Paediatrics, University of Oxford, South Parks Road, Oxford OX1 3QX, UK; [4]Institute of Developmental and Regenerative Medicine, University of Oxford, IMS-Tetsuya Nakamura Building, Old Road Campus, Roosevelt Dr, Headington, Oxford OX3 7TY, UK; [5]Charles River Laboratories, Darwinweg 24, Leiden, 2333 CR South Holland, the Netherlands; [6]Merck KGaA, Frankfurter Str. 250, 64293 Darmstadt, Germany; [7]Celvia CC AS, 50411 Tartu, Estonia; [8]Department of Obstetrics and Gynaecology, Institute of Clinical Medicine, University of Tartu, 50411 Tartu, Estonia; [9]Department of Laboratory Medicine, Karolinska Institutet, 141 86 Huddinge, Sweden; [10]MDUK Oxford Neuromuscular Centre, South Parks Road, Oxford, UK

Exon skipping is a leading therapeutic approach for Duchenne muscular dystrophy (DMD), whereby modulation of pre-mRNA splicing is used to restore the dystrophin translation reading frame. Four exon skipping drugs have received FDA accelerated approval, despite limited clinical efficacy. To investigate how treatment timing influences exon skipping outcomes, dystrophin-deficient *mdx* mice were injected with peptide-conjugated phosphorodiamidate morpholino oligonucleotide (PPMO) exon skipping conjugates beginning at adult (12-week-old) or aged (75-week-old) stages, followed by biochemical and transcriptomic analyses in tibialis anterior muscles. Mean *Dmd* exon 23 skipping was 79% in adults and 44% in aged PPMO-treated *mdx* mice, whereas dystrophin protein restoration was 35% and 8%, respectively. Histopathological improvements were evident only in the adult treated mice. PPMO-treatment in adult *mdx* mice induced a broad transcriptomic shift toward a wild-type signature, whereas treatment in aged mice resulted in negligible gene expression changes, indicating that late intervention fails to reverse disease-associated pathologies despite low-level dystrophin restoration. Increased expression of the dystrophin-repressing microRNA miR-31-5p, which was more strongly upregulated in aged *mdx* muscle, provides a potential mechanistic explanation. In conclusion, PPMO-mediated exon skipping is substantially more effective when initiated in adult vs. aged dystrophic muscle, supporting early therapeutic intervention in DMD-affected individuals.

INTRODUCTION

Duchenne muscular dystrophy (DMD) is an X-linked, monogenic muscle-wasting disorder caused by pathogenic variants in the gene encoding the dystrophin protein (human: *DMD*, mouse: *Dmd*) with an incidence of 1 in 5,000 live male births.[1] Dystrophin is a structural and signaling protein which acts to assemble a complex of proteins at the sarcolemma (the dystrophin-associated protein complex, DAPC). Loss of dystrophin sensitizes muscle to contractile damage,[2] leading to cycles of muscle turnover (i.e., myonecrosis and compensatory regeneration) and persistent inflammation. As a result, muscle quality declines over time, with progressive replacement of myofibers with connective and adipose tissue (i.e., fibro-fatty degeneration). Patients with DMD typically present with signs of muscle weakness, delays in the achievement of motor milestones, and movement difficulties. Loss of ambulation typically occurs in late childhood, although there is substantial inter-individual variability in disease progression. DMD is ultimately fatal, most commonly due to cardiac or respiratory failure, with survival historically into the third decade but increasingly extending beyond this in the modern treatment era.[3–5]

Standard-of-care treatment of DMD involves long-term multidisciplinary care[6–8] and corticosteroid therapy, the latter of which has been demonstrated to delay the loss of ambulation by a few years[7,9,10] but is associated with a number of undesirable side effects[11] As such, DMD remains a substantial health burden, underscoring the need for therapies that meaningfully modify disease progression.

Recent years have seen substantial progress in the field of experimental therapeutics for DMD.[12] Various strategies have been employed to restore dystrophin. For example, antisense oligonucleotide (ASO)-mediated exon skipping can be utilized to modulate alternative splicing such that the dystrophin translation reading frame is restored.[13–16] Four such ASO drugs received accelerated approval

Received 12 January 2026; accepted 15 July 2026;
https://doi.org/10.1016/j.omtn.2026.103017.

Correspondence: Thomas C. Roberts, Institute of Developmental and Regenerative Medicine, University of Oxford, IMS-Tetsuya Nakamura Building, Old Road Campus, Roosevelt Dr, Headington, OX3 7TY Oxford, UK.
E-mail: thomas.roberts@idrm.ox.ac.uk

Published by Elsevier Inc. on behalf of The American Society of Gene and Cell Therapy.
This is an open access article under the CC BY license (http://creativecommons.org/licenses/by/4.0/).

from the US FDA for the treatment of DMD (eteplirsen, viltolarsen, golodirsen, and casimersen). While a major achievement for the DMD research and patient communities, the dystrophin restoration achieved by these compounds is minimal[17–20] and of debatable clinical benefit.[21–26] Similarly, the FDA approved the first gene therapy for DMD (Elevidys) in 2023,[27] despite failure to reach its clinical endpoint in a Phase 3 trial.[28] Furthermore, the safety of Elevidys (and high dose adeno-associated virus [AAV]-based gene therapies in general) has been called into question following multiple patient deaths.[29]

The current generation of FDA-approved exon skipping ASOs are all phosphorodiamidate morpholino oligonucleotides (PMOs). This chemistry does not occur in nature and is widely regarded as being safe.[30] However, PMO delivery to skeletal (and especially cardiac) muscle is very limited, which has motivated a search for delivery-assisting moieties that can enhance their activity. Our group and others have extensively explored the use of covalent conjugation of PMOs to cell-penetrating peptides for improved oligonucleotide delivery.[31–34] Peptide-conjugated PMOs (PPMOs) are far more potent than unconjugated PMOs, exhibit enhanced cellular internalization driven by endocytosis, and importantly, exhibit exon skipping activity in cardiac tissue.[31–36] PPMOs are currently being commercially developed by PepGen Ltd (for myotonic dystrophy) and Entrada Therapeutics (for DMD).[37,38] The benefits of peptide conjugation need to be balanced against safety concerns, particularly those related to nephrotoxicity. Proximal renal tubule damage has been reported for some PPMO conjugates in mice and cynomolgus monkeys.[39,40] Clinical trials for the PPMO drugs EDO-051 (PepGen) and vesleteplirsen (Sarepta) both reported hypomagnesemia and, in some cases, declining renal function, leading both companies to discontinue their development.[38,41–43]

Notably, other delivery assisting moieties for enhancing DMD therapeutics are currently being evaluated in clinical trials, including PMO-antibody and PMO-FAb fragment conjugates, developed by Avidity Biosciences and Dyne Therapeutics, repsectively.[44,45]

The *mdx* mouse is the most commonly used murine model of DMD, which carries a premature termination codon in *Dmd* exon 23 and exhibits some aspects of DMD pathology.[46,47] Skipping of this exon restores the dystrophin translation reading frame. We have previously performed a variety of omics analyses in this mouse with a focus on the effects of dystrophin restoration therapies, including gene expression/microRNA (miRNA) microarray,[48,49] small RNA-seq,[50] and high resolution mass spectrometry-based proteomics.[48,51]

A number of factors are important when considering the efficacy of dystrophin restoration therapies. (1) The total amount of dystrophin restored. It is widely accepted that the more dystrophin that can be restored, the better. Various lines of evidence suggest that ∼10% of healthy dystrophin levels is a sensible threshold for therapeutic benefit.[52–54] (2) The quality of dystrophin restored. Exon skipping splice correction and microdystrophin gene therapies both rely on the generation of internally deleted quasi-dystrophin proteins that resemble those found in some patients with Becker muscular dystrophy,[55] and which are associated with mild pathology. However, such quasi-dystrophins are unlikely to fully compensate for the loss of the full-length dystrophin protein. There is a growing appreciation that full-length dystrophins are desirable, which presents a challenge, given that the size of the dystrophin open reading frame (ORF) (∼11 kb) exceeds the packaging capacity of AAV vectors (∼4.6 kb). Restoration of full-length dystrophin has been demonstrated in human clinical trial participants treated with an exon skipping therapy to treat DMD exon 2 duplication.[56] Furthermore, there are multiple other efforts to develop full-length dystrophin delivery strategies using split-vector approaches,[57–59] or by using vectors with higher packaging capacities.[60–62] (3) Correct localization of dystrophin at the sarcolemma. Work from our group and from others has shown that uniform distribution of dystrophin protein along the sarcolemma within a myofiber is an important determinant of therapeutic success.[63–67] Importantly, exon skipping treatment with PPMO conjugates induced a uniform pattern of sarcolemmal dystrophin coverage (even at low doses),[64] while CRISPR-Cas9-mediated exon deletion resulted in a patchy pattern of dystrophin distribution.[65] This is an important observation, as incomplete sarcolemmal dystrophin coverage is likely to be insufficient to prevent myofiber turnover and correct disease pathology.[63] (4) Optimal timing for initiation of treatment. Early initiation of dystrophin restoration therapies may be beneficial to prevent or delay pathological degeneration of muscle. However, for "one-and-done" therapies, such as microdystrophin gene therapy or CRISPR-Cas9-mediated gene correction, the number of corrected nuclei will be progressively diluted as non-corrected myonuclei are added to myofibers as a consequence of growth and regeneration.

In this study, we assessed the efficacy of exon skipping by using PPMO ASO conjugates when the treatment was initiated at the adult and aged stages in mice. These data support the notion that early treatment may be beneficial in the treatment of DMD.

RESULTS

Experimental design

To investigate the efficacy of exon skipping treatment at different ages, *mdx* mice were treated with a PPMO Pip6a-PMO (Figure 1A), which we have previously shown as being effective at inducing skipping of *Dmd* exon 23 and restoring dystrophin protein expression in *mdx* mice.[31,33,48,50,68] For "adult" mice ($n = 3$), a single 12.5 mg/kg dose of PPMO was administered intravenously at 12 weeks of age and mice sacrificed at 14 weeks of age (Figure 1B). For "aged" mice ($n = 3$), three doses of 10 mg/kg PPMO were administered intravenously at 75, 76, and 77 weeks of age and the mice sacrificed at 78 weeks of age. The age of adult mice was selected as this is a time point after the initial regenerative "crisis" period. The adult-stage dosing regimen reflects a well-established and reproducible paradigm in our laboratory.[31,33,48,52,68] The aged mice were selected based on previous demonstrations of advanced histopathology in this model,[69] which we reasoned would be more reflective of

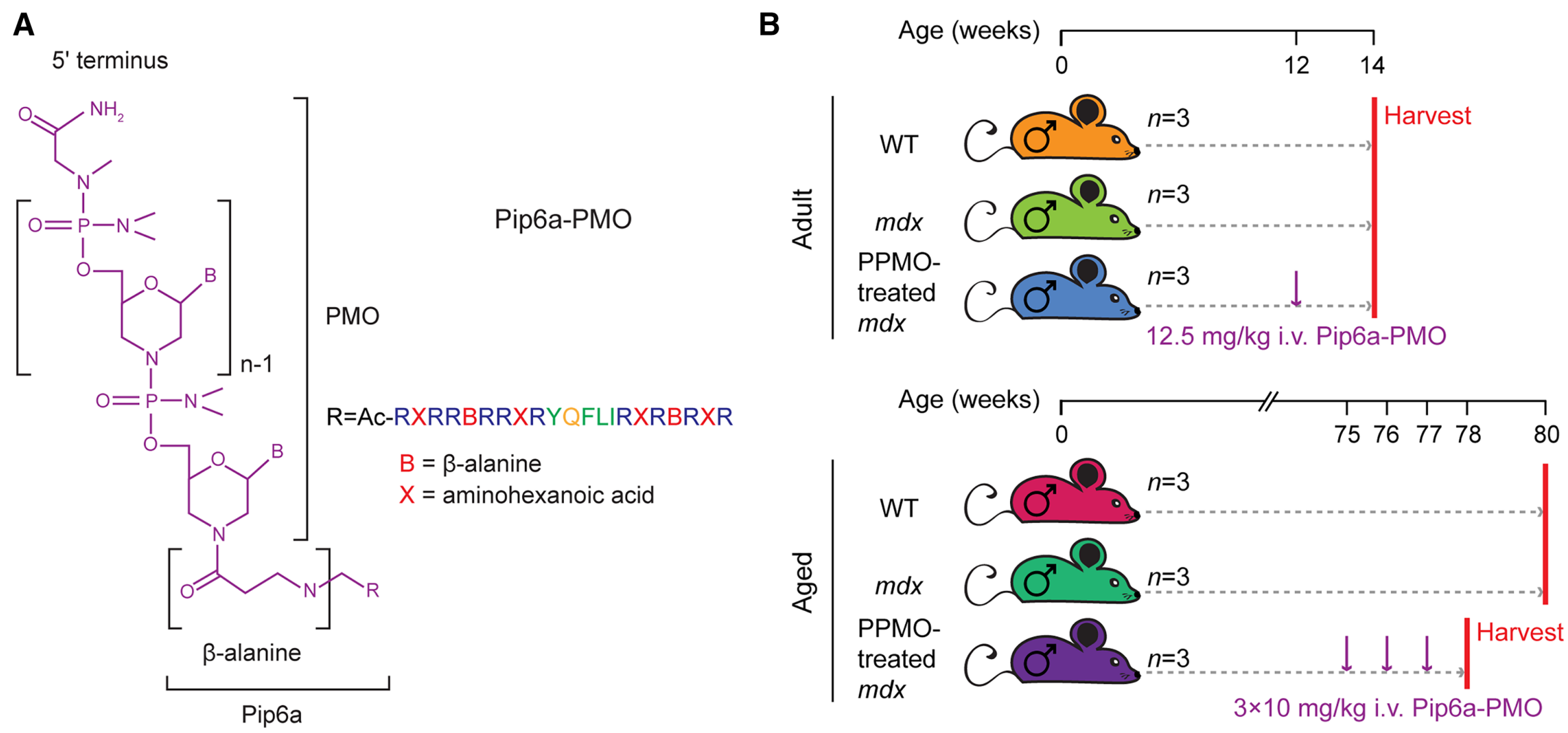

Figure 1. Exon skipping-mediated dystrophin restoration in adult and aged *mdx* mice

(A) Structure of the PPMO conjugates, which consist of a Pip6a peptide covalently conjugated to a PMO antisense oligonucleotide designed to skip *Dmd* exon 23. (B) Schematic of the experimental design. Male *mdx* mice were treated with Pip6a-PMO (PPMO) conjugates with doses and regimens as indicated and harvested at 14 weeks of age for "adult mice" and at 78 weeks for "aged" mice. Age- and sex-matched untreated WT and *mdx* mice were harvested in parallel (all groups, $n = 3$).

dystrophic muscle in individuals with DMD. The aged-stage multiple-dosing regimen was employed to maximize drug accumulation/exposure in muscle with more advanced disease. Age-, strain-, and sex-matched wild-type (WT, C57BL/10) and dystrophic *mdx* mice were harvested in parallel as controls (all $n = 3$). Tibialis anterior (TA) muscles were macrodissected at sacrifice and subjected to a panel of biochemical analyses.

PPMO treatment initiated at the adult stage results in greater dystrophin restoration than treatment initiated at the aged stage

Mean levels of *Dmd* exon 23 skipping were determined to be ∼79% and ∼44% for the adult and aged PPMO-treated *mdx* mice, respectively ($p < 0.05$), as determined by RT-qPCR (Figure 2A). Restoration of dystrophin protein expression was assessed by western blot, whereby robust re-expression was observed in the adult PPMO-treated *mdx* mice, while the treatment was much less effective in the aged mice (Figure 2B). Western blot quantification revealed mean dystrophin protein levels were ∼35% and ∼8% of WT levels for the adult and aged PPMO-treated *mdx* mice, respectively ($p < 0.05$, Figure 2C). Tissue-associated PMO content was quantified in TA muscle lysates and found to be ∼5.3-fold higher in aged relative to adult PPMO-treated *mdx* muscle ($p < 0.05$, Figure 2D).

Analysis of transverse TA muscle sections revealed a uniform pattern of sarcolemmal dystrophin expression in the PPMO-treated *mdx* animals at both ages (Figure 3A). By contrast, untreated *mdx* control animals were dystrophin negative, with the exception of sporadic revertant fibers. Untreated adult *mdx* mice exhibited characteristic histopathological features of dystrophin absence, including widespread centrally nucleated fibers and an increase in connective tissue surrounding myofibers (i.e., fibrosis), as assessed by hematoxylin and eosin staining (Figure 3B). PPMO-treated *mdx* adult animals exhibited a reduction in fibrotic damage, while the majority of myofibers were still centrally nucleated (consistent with them having recently undergone regeneration). We also observed that PPMO-treated adult myofibers appeared hypertrophic, consistent with previous observations.[63] Histopathological features were more pronounced in the aged *mdx* animals, whereby widespread fibrosis was observed together with irregular fiber sizes and central nucleation. In contrast with the adult mice, PPMO-treatment did not reverse muscle histopathology in the aged *mdx* mice (Figure 3B).

Taken together, these data indicate that PPMO treatment initiated at the adult stage is associated with substantially greater dystrophin protein restoration and histopathological improvement than treatment initiated at the aged stage, despite the higher overall dosing regimen administered to aged *mdx* animals.

PPMO treatment induces widespread correction of the dystrophic transcriptome at the adult stage

To better understand the gene expression changes that occur in adult and aged dystrophic muscles, we performed bulk RNA-seq analysis in all experimental groups described earlier (Figure 1B). Total RNA samples extracted from TA muscle sections were subjected to Illumina RNA-seq analysis with a target read depth of 50 million reads per sample. Libraries were total RNA, single-end, stranded, and rRNA-depleted. Prior to sequencing, a number of quality control analyses were performed that were indicative of high sample quality, including RNA quantification (pre- and post-rRNA depletion),

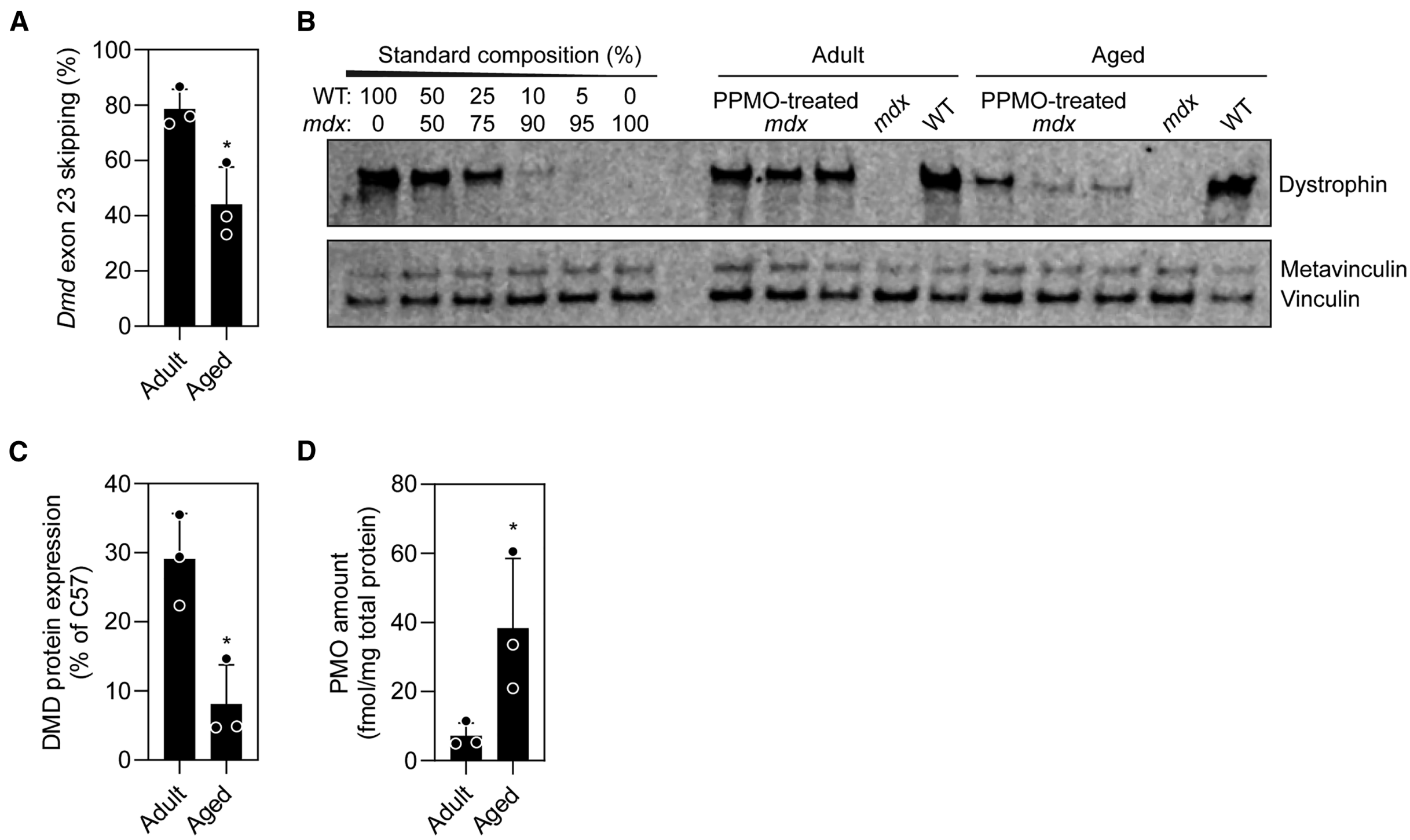

Figure 2. Exon skipping-mediated dystrophin restoration in Adult and Aged *mdx* mice

(A) RT-qPCR analysis of *Dmd* exon 23 skipping levels. (B) Western blot of dystrophin protein expression for all PPMO-treated *mdx* animals. Vinculin was used as a loading control. (C) Quantification of dystrophin protein expression. (D) Quantification of muscle PMO content using an ELISA-based method. All vales are mean + SD ($n = 3$). Statistical significance was assessed by Student's t test, $p < 0.05$.

RNA integrity assessment by Bioanalyzer, and assessment of genomic DNA contamination) (Figure S1).

Sequencing reads were processed using a custom in-house pipeline and analyzed to identify differentially expressed genes. Significantly differentially expressed genes ($p < 0.01$) were visualized by heatmap analysis, organized by unsupervised hierarchical clustering (Figure 2A). Replicate libraries clustered together for all experimental groups, indicative of low inter-replicate variance. Furthermore, WT samples were found to cluster together for both adult and aged samples. Conversely, the remaining samples formed the next largest cluster, with the adult and aged samples forming subclusters (containing a mixture of both *mdx* and PPMO-treated *mdx* samples). Inspection of the heatmap revealed an important qualitative difference between the adult and aged samples. Specifically, adult PPMO-treated animals clustered toward the WT samples, whereas aged PPMO-treated animals clustered with the *mdx* samples (Figure 4A).

A similar clustering pattern was observed by principal component analysis (PCA). In general, the difference between dystrophic and WT animals was represented by PC1, which contained 50.9% of the variance (Figure 4B). By contrast, PC2 contained 28.7% of the variance, which, to some extent, reflected the difference between adult and aged samples (Figure 4B). In adult animals, PPMO-treated *mdx* animals clustered toward the WT libraries, indicative of a shift (in PC1) from a dystrophic pattern of gene expression toward a more WT-like transcriptome. By contrast, aged PPMO-treated samples were very closely clustered with the age-matched *mdx* libraries (Figure 4B).

To investigate patterns of differential expression, volcano plots were generated for: (1) *mdx* vs. WT, (2) PPMO vs. WT, and (3) PPMO vs. *mdx* comparisons for both adult (Figure 5A) and aged (Figure 5B) samples. The greatest number of differentially expressed genes (DEGs) was observed in the adult *mdx* vs. WT comparison (3,946 DEGs). In adult animals, treatment with PPMO induced profound transcriptomic changes (2,085 DEGs) indicative of a partial reversal in gene expression changes associated with loss of dystrophin. By contrast, there were almost no significant differences between aged PPMO-treated and *mdx* libraries (7 DEGs).

A reduction in *Dmd* mRNA levels is a characteristic feature in *mdx* mice,[48,70,71] which has been attributed to a transcriptional mechanism.[72] Accordingly, in adult animals, normalized *Dmd*-mapping read counts were reduced by ~50% and was restored to near-WT levels by PPMO treatment (Figure 5C). By contrast, in aged animals, normalized *Dmd* transcript abundance across the locus was reduced by ~30% in *mdx* animals and was not restored by PPMO treatment (Figure 5C). (Note: *Dmd* transcript imbalance is discussed in the following section).

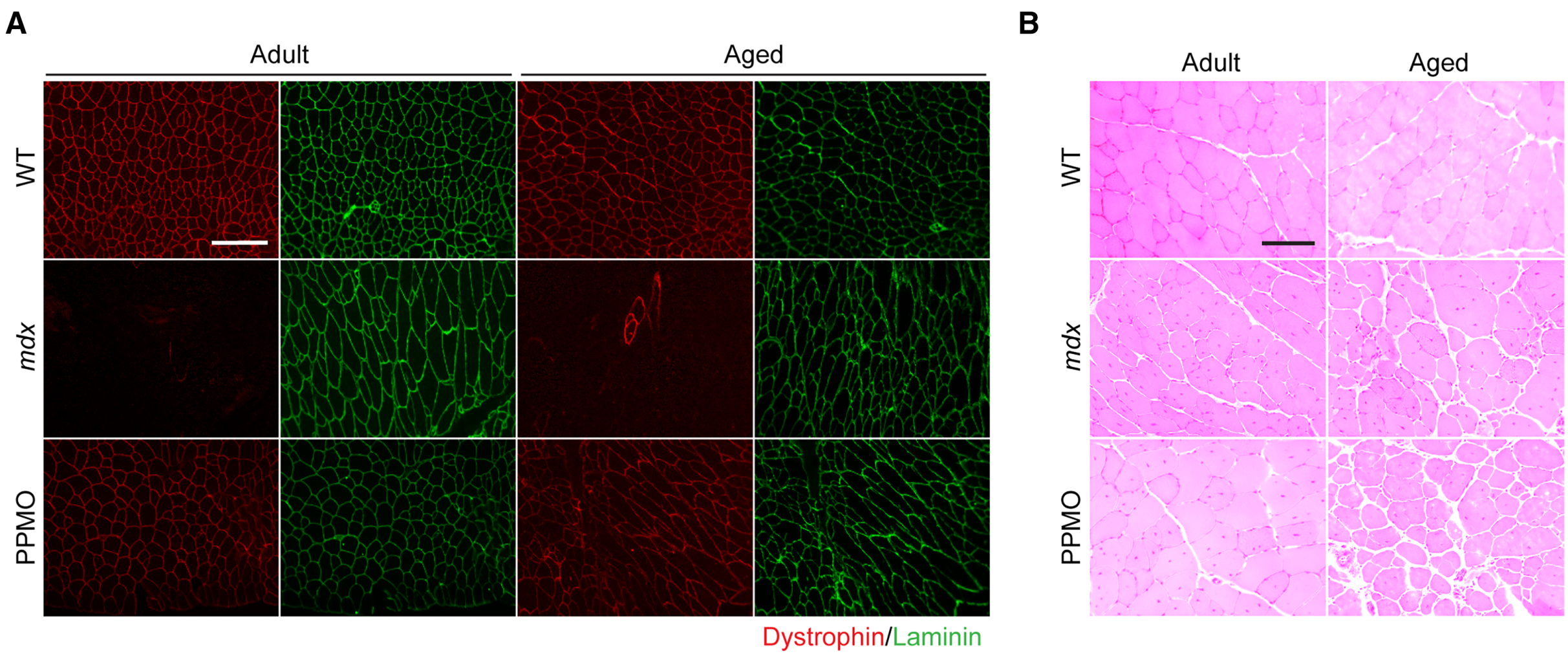

Figure 3. Restoration of sarcolemmal dystrophin expression and histopathological assessment in adult and aged *mdx* mice
(A) Representative dystrophin immunofluorescence micrographs of TA muscle sections in transverse orientation. Sections were co-stained with laminin to delineate myofiber boundaries. (B) H&E staining of TA muscle sections. Images were taken at 20× magnification and scale bars represent 100 μm.

Together, these data are indicative of widespread transcriptional perturbations in dystrophic muscle, which are more complex at the adult time point. PPMO treatment results in a shift in the transcriptional profile of adult dystrophic muscle toward that of WT muscle. By contrast, PPMO treatment of aged muscle had negligible influence on the dystrophic transcriptome.

PPMO treatment reverses *Dmd* transcript imbalance in adult dystrophic muscle

mdx animals are known to exhibit a transcript imbalance effect, whereby dystrophic muscle contains greater quantities of the 5' end of the *Dmd* transcript and reduced levels of the 3' end, as compared with WT muscle.[73,74] This phenomenon is not well understood, but it is thought to arise due to challenges associated with the unusually large *Dmd* locus, including impaired transcriptional elongation and altered transcript stability in dystrophic muscle.[72–74] We observed *Dmd* transcript imbalance in both adult and aged *mdx* mice when RNA-seq data were analyzed for differential exon usage through DEXSeq[75] (Figure 6). Treatment with PPMO reversed the transcript imbalance effect in adult *mdx* mice (Figures 6A and 6B) but not in aged *mdx* mice (Figures 6C and 6D). Notably, it has previously been reported that transcript imbalance is not corrected by exon skipping therapy, although the threshold of dystrophin restoration required to correct this phenomenon may not have been achieved in these studies.[74,76] These findings were further investigated by reverse transcription-droplet digital PCR (RT-ddPCR) using primers spanning *Dmd* exons 1–2, exons 44–45, and exons 63-64. These analyses recapitulated key findings including the presence of transcript imbalance in the *mdx* animals at both ages, the almost complete correction of transcript imbalance in the adult treated *mdx* animals, and a diminished correction in the aged *mdx* animals at the 3'-most exons measured (Figure S2).

Differential gene expression signatures in dystrophic muscle

To characterize the biological meaning of gene expression changes occurring in dystrophic muscle, we performed gene list enrichment analysis and semantic space reduction on DEG lists for the *mdx* vs. WT at the adult (Figure S3) and aged (Figure S4) stages. These analyses identified some expected perturbed pathways, including calcium ion binding, electron transfer activity, extracellular matrix (ECM) organization, immune system process, reactive oxygen species (ROS) metabolic process, and regulation of nitric oxide metabolic process, but also many other Gene Ontology (GO) terms that were mostly generic and not otherwise informative. By contrast, manual inspection of gene expression changes revealed gene expression signatures that are consistent with known aspects of dystrophic pathology. *Myh3* (embryonic myosin heavy chain) was upregulated at both adult and aged time points, although the magnitude of upregulation was much higher at the adult stage. Interestingly, PPMO-treatment restored *Myh3* expression to WT levels in both cases, indicative of a stabilization of muscle turnover, even in aged muscle (Figure S5A). Concordantly, *Pax7* expression was highly increased in *mdx* muscle at the adult stage only. This corresponds with the high level of muscle regeneration occurring at this age, with a concomitant expansion of the satellite (stem) cell pool (Figure S5B). The senescence markers *Cdkn1a* (p21) and *Cdkn2a* (p16) were strongly upregulated in *mdx* muscle, with *Cdkn1a* more prominent at the adult stage and *Cdkn2a* more pronounced at the aged stage. This is indicative of accumulated cell stress, which may contribute to a functionally compromised regenerative niche, especially in aged dystrophic muscle (Figure S5C). Neuronal nitric oxide synthase protein mislocalization is a characteristic feature of dystrophic muscle. However, we also observed downregulation of *Nos1* transcripts, which was more apparent at the aged time point (Figure S5D). *Nos3* was similarly downregulated in dystrophic muscle. *Vegfa* and its receptor *Kdr* were also downregulated in dystrophic

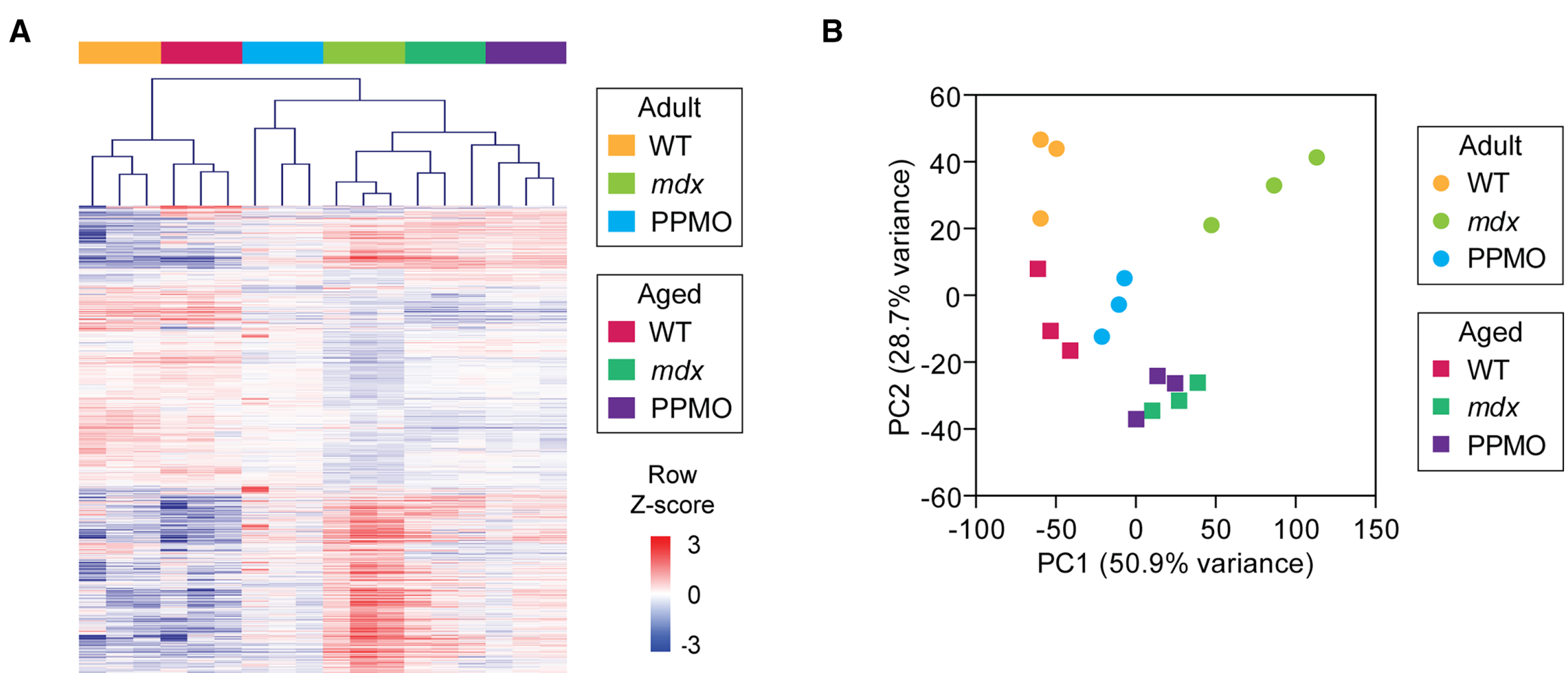

Figure 4. RNA-seq analysis in adult and aged dystrophic muscle

RNA-seq libraries from WT, *mdx*, and PPMO-treated *mdx* at both adult and aged time points were analyzed for differential expression. Data are visualized by (A) heatmap (unsupervised hierarchical clustering) and (B) principal component analysis (PCA) plot.

muscle. These data are consistent with gene expression changes that promote functional ischemia in *mdx* mouse muscle (Figure 5D).

Numerous transcripts encoding proteins involved in ROS handling were downregulated in dystrophic muscle, including *Egln1*, *Sod2*, *Ndufs1*, and *Prdx3* (Figure S6). Additionally, *Ucp3*, *Epas1*, *Cox4i1*, and *Txnrd1* were similarly downregulated, with aged animals exhibiting a further decrease (Figure S6). The antioxidant *Gpx3* was highly elevated in aged WT animals, but did not show a corresponding increase in aged *mdx* animals. These findings are consistent with a failure of dystrophin-deficient muscle to effectively manage ROS production. *Ppargc1* (PGC1-α) was downregulated in *mdx* tissues at both ages (Figure S6), consistent with a suppression of mitochondrial biogenesis and oxidative metabolism, which may further contribute to the oxidative stress phenotype in dystrophic muscle.

Persistent inflammation is a characteristic feature of dystrophic muscle, which was reflected in the RNA-seq gene expression patterns (Figure S7A). Specifically, leukocyte (*Ptprc*), T cell (*Cd3e* and *Cd4*), and macrophage/monocytes (*Cd68* and *Itgam*) markers were upregulated in dystrophic muscle. The dendritic cell marker (*Itgax*) was strongly upregulated at the adult stage, possibly associated with the peak of active regeneration observed at this time point. Transcript levels for cytokines and chemokines (e.g., *Tnf*, *Il1b*, *Il6*, and *Ccl2*) were undetectable or not-significantly changed between libraries (data not shown). Components of the non-canonical NF-κB pathway (*Relb* and *Nfkb2*) were significantly increased in *mdx* muscle (Figure S7B), consistent with a chronic, low-grade inflammatory or inflammaging-like state.

Markers of fibrosis were upregulated in *mdx* muscle, including *Col1a1*, *Col3a1*, *Col6a1*, *Fn1*, *Ctgf*, *Timp1*, *Mmp2*, and *Postn* (Figure S8A). Interestingly, these transcripts exhibited very similar patterns of expression, with a higher degree of upregulation in adult *mdx* muscle than in aged *mdx* muscle. This pattern of expression may reflect the shift from the period of active regeneration observed in the adult *mdx* mice (with associated intense ECM remodeling) to a period of more stable period of fibrotic decline. We have previously observed an elevation in COL3A1 protein expression only in aged dystrophic mice (*mdx* and *mdx52* strains) by mass-spectrometry-based proteomics analysis, highlighting a divergence between gene expression and tissue protein levels. Notably, *Col1a1* and *Col3a1* were reduced in aged WT muscle,[51] further highlighting that differences in the expression of these transcripts in dystrophic muscle substantially diverge from the pattern expected during the course of normal aging. *Tgfb1* was significantly upregulated in *mdx* mice but only at the adult stage. Markers of adipo-degeneration were not statistically different between *mdx* and C57 libraries (i.e., *Pparg*, *Cepba,* and *Fabp4*, *Plin1*, and *Adipoq;* data not shown) consistent with minimal pathology of this type observed in *mdx* mice. The fibro-adipo progenitor (FAP) cell marker *Pdgfra* was significantly increased by similar amounts in *mdx* tissues at both adult and aged stages (Figure S8B). Conversely, *Ly6a* (Sca-1) expression was not statistically significant between *mdx* and C57 libraries.

We next sought to investigate possible reasons for impaired re-expression of dystrophin expression in aged *mdx* muscle following PPMO treatment. Firstly, we investigated the possibility that a global downregulation of translation may underlie the impairment in dystrophin re-expression. The integrated stress response (ISR) is a pathway that controls the response to cellular stresses via the phosphorylation of eIF2α (eukaryotic initiation factor 2 alpha), which results in a global downregulation of translation while promoting selective activation of stress-responsive genes. Transcript levels for

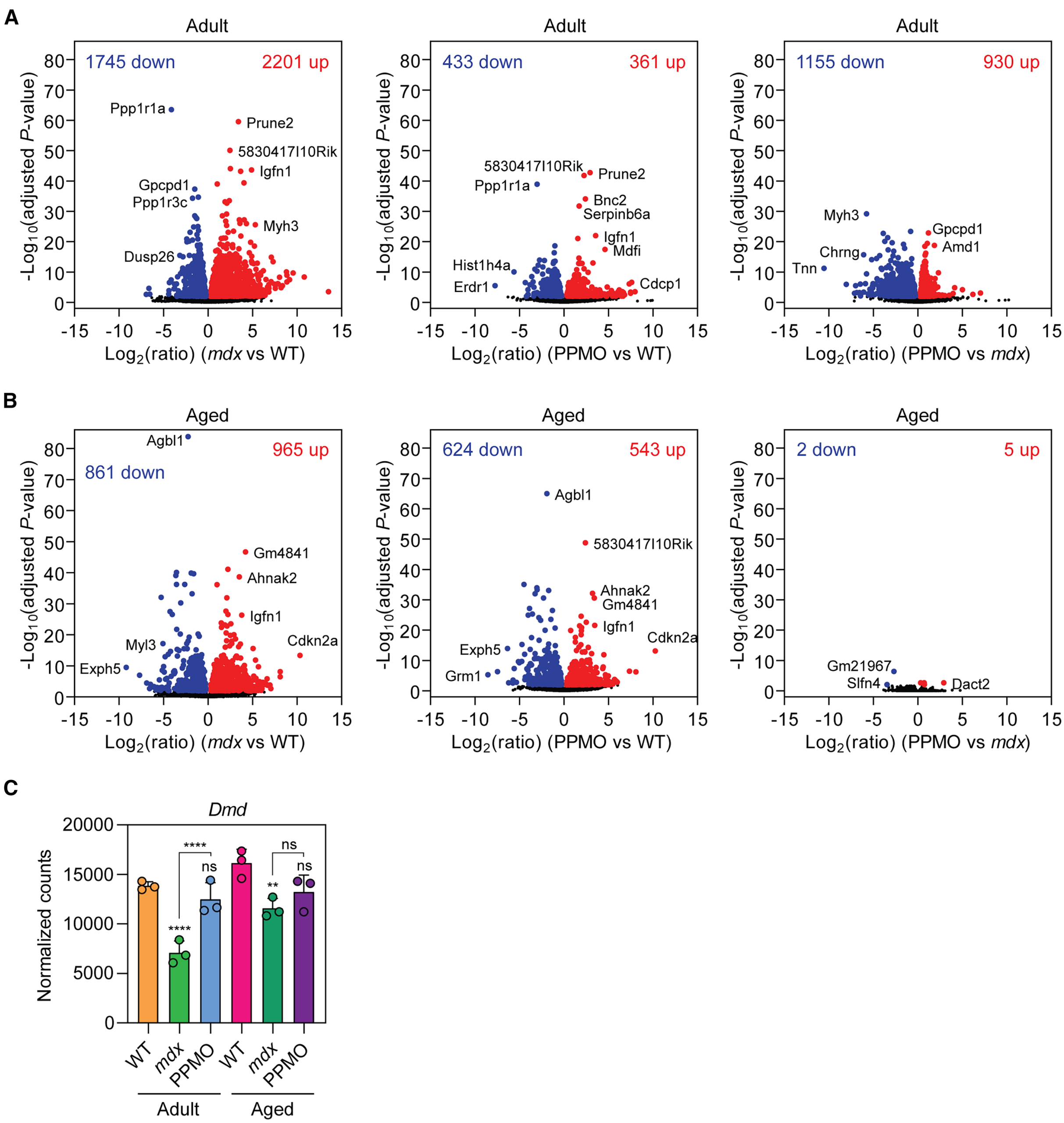

Figure 5. Differential expression analysis in adult and aged dystrophic muscle

RNA-seq libraries from WT, *mdx*, and PPMO-treated *mdx* at both adult and aged time points were analyzed for differential expression. Volcano plots for each differential expression comparison for (A) adult and (B) aged mice. (C) RNA-seq normalized counts data for the *Dmd* transcript for all libraries. Statistical significance was assessed by using DESeq2 and Benjamini-Hochberg-adjusted p values indicated. Vales are mean + SD (n = 3).Statistical comparisons are relative to the adult WT group unless otherwise indicated, ****adjusted $p < 0.0001$; ns, not significant.

key ISR components were unchanged across all libraries (*Atf4*, *Ddit3*, *Eif2ak1*, *Eif2ak2*, *Eif2ak3*, *Eif2ak4*, and *Eif2s1*, and *Ppp1r15a* [GADD34]; data not shown). Similarly, transcript levels for multiple downstream ATF4 targets were unchanged (*Asns*, *Trib3*, and *Psat1*, and *Ppp1r15a* [GADD34]; data not shown), suggesting that the ISR is not engaged in this context. Similarly, transcripts associated with the unfolded protein response (UPR) arm of the ISR were similarly unchanged (*Hspa5*, *Hsp90b1*, *Pdia3*, and *Calr*; data not shown). While ISR activation has been reported in DMD muscle and dystrophic animal models,[77,78] the data presented herein argue against the ISR involvement as a mechanism underlying impaired dystrophin re-expression in aged *mdx* muscle.

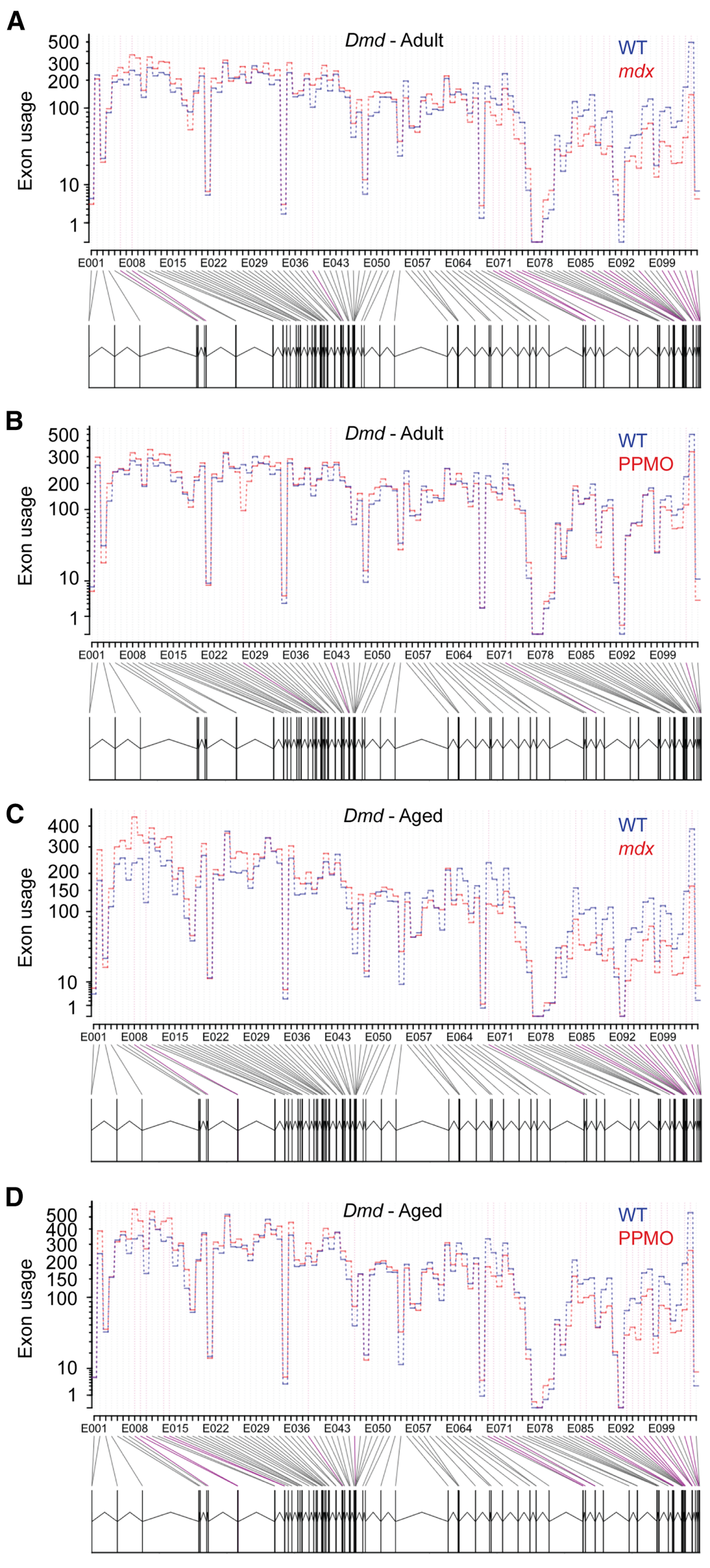

Figure 6. PPMO treatment restores *Dmd* transcript imbalance in adult but not aged *mdx* mice

Differential exon usage was calculated using DEXSeq for the *Dmd* transcript and visualized using RNA-seq data for four comparisons: (A) adult *mdx* vs. WT, (B) adult PPMO vs. WT, (C) aged *mdx* vs. WT, and (D) aged PPMO vs. WT. DEXSeq analysis reflects relative exon usage after normalization for sequencing depth and overall gene expression rather than absolute transcript abundance. Exons showing statistically significant differential usage are highlighted with purple lines.

The AKT/PI3K/mTOR signaling axis is an important regulator of protein synthesis in muscle. Analysis of transcript levels associated with this pathway points to a mixed picture, with no significant changes in *Ddit4*, *Tsc1*, *Slc1a5*, *Atg13*, *Trim63*, *Eif4g1*, *Mtor*, *Map1lc3b*, *Tsc2*, *Pik3r1*, or *Eif4ebp1* (data not shown). *Akt1* and *Myc* were elevated in adult *mdx* muscle, suggestive of a pro-growth phenotype consistent with muscle regeneration occurring at this age (Figure S9). However, *Slc38a2*, *Pik3ca*, *Prkaa2*, *Rheb*, *Insr*, *Rps6kb1*, *Rictor*, *Eif4e*, and *Fxbo32* were downregulated in adult *mdx* muscle, indicative of potentially reduced AKT/PI3K/mTOR pathway activity (Figure S9). *Igf1r* was downregulated in aged *mdx* muscle (Figure S9). Notably, previous proteomics analyses from our group identified the PI3K/AKT pathway as being activated in dystrophic muscle at 8 and 16 weeks and repressed in 80-week-old mice.[51] These data suggest that attenuated mTOR signaling may contribute to impaired dystrophin re-expression in aged dystrophic muscle.

Differential expression of molecular chaperones may influence dystrophin protein folding, stability, and post-translational processing in the context of exon skipping-mediated therapeutic restoration. To investigate this possibility, we examined transcriptomic changes in members of HSP70 and HSP90 molecular chaperone families. For the HSP70 family, there were no significant differences between *mdx* and C57 libraries in transcript levels for *Hspa1a* and *Hspa1b* (Figure S10A). Conversely, *Hspa8* was downregulated in both *mdx* and PPMO-treated *mdx* animals at the adult stage. There was no difference in *Hspa8* expression at the aged stage, although levels were significantly reduced in aged C57 vs. adult C57 libraries (Figure S10A). For the HSP90 family, there were no significant differences between any groups for *Hsp90aa1* and *Hsp90ab1* (Figure S10B). These data suggest that transcriptomic changes in the HSP70 and HSP90 chaperone families are unlikely to explain the insensitivity of aged dystrophic muscle to exon skipping therapy.

The ubiquitin-proteasome system is a major regulator of protein turnover in skeletal muscle, alterations in which could potentially influence the stability of restored dystrophin protein after exon skipping therapy. Expression of the E1 ubiquitin-activating enzyme *Uba1* was unchanged between all groups (Figure S11). Among the E2 ubiquitin-conjugating enzymes, expression of *Ube2a* was reduced in adult stage *mdx* tissue (Figure S11). *Ube2b* was downregulated in *mdx* muscle at both adult and aged stages. Notably, expression of *Ube2b* was partially restored toward WT levels by PPMO treatment at the adult stage but not at the aged stage (Figure S11). Similarly, *Ube2d1* and *Ube2n* were downregulated in adult *mdx* muscle and fully restored to WT levels by PPMO treatment but were unchanged between groups at the aged stage. For the E3 ubiquitin ligases, there were no significant changes observed for *Trim63* (MuRF1) between groups (Figure S11). Expression of *Fbxo32* (Atrogin-1) is discussed earlier in the context of the AKT/PI3K/mTOR axis (Figure S9). Together, these findings suggest that there is no clear age-associated ubiquitin-proteasome

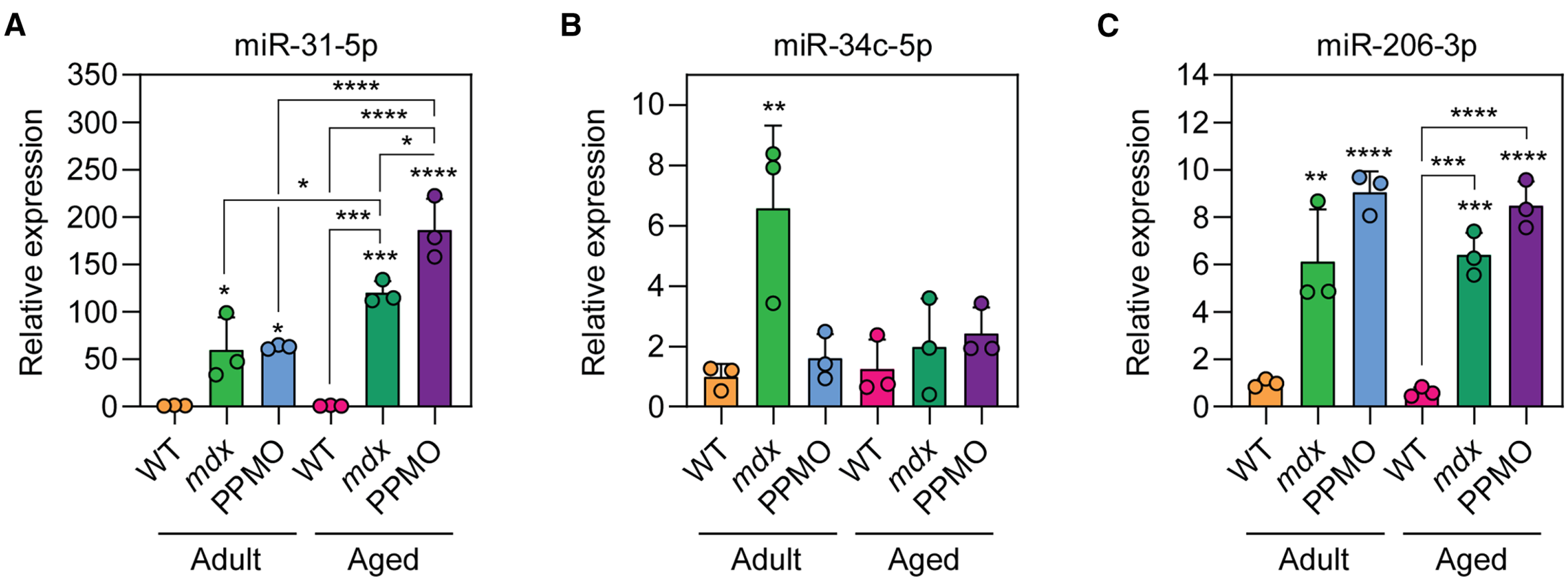

Figure 7. Upregulated miR-31 expression in adult dystrophic muscle is further increased in aged dystrophic muscle

RNA samples from WT, *mdx*, and PPMO-treated *mdx* mice at adult and aged time points were analyzed by RT-qPCR to determine differential miRNA expression for (A) miR-31-5p, (B) miR-34c-5p, and (C) miR-206-3p (normalized to miR-16-5p). All values are mean + SD ($n = 3$). Statistical significance was tested by one-way ANOVA with Tukey's post hoc test. Statistical comparisons are relative to the adult WT group unless otherwise indicated; *$p < 0.05$, **$p < 0.01$, ***$p < 0.001$, ****$p < 0.0001$.

system signature that explains the reduced dystrophin restoration effect in aged muscle.

Autophagy is an important regulator of skeletal muscle proteostasis and organelle turnover, and impaired autophagic pathways have been implicated in dystrophic muscle pathology and aging.[79] To investigate whether altered autopahgy might contribute to the differential response to exon skipping therapy, we examined the expression of genes involved in autophagosome formation and autophagic regulation in our transcriptomics dataset. *Map1lc3a* (LC3A) was unchanged at the adult stage and modestly increased in aged *mdx* muscle (with a partial restoration toward WT levels following PPMO treatment, Figure S12). Conversely, there were no significant changes in *Map1lc3b* (LC3B), *Sqstm1* (p62), or *Becn1* between any groups across both ages (Figure S12). Collectively, these data suggest that there is no obvious perturbation in the expression of autophagy-associated genes that could explain the lack of therapeutic response to exon skipping in the aged *mdx* animals used in this study.

While analysis of transcriptomic signatures has the potential to identify pathways and cellular functions that may be perturbed in dystrophic muscle, the use of bulk RNA-seq analysis presents a number of important limitations. Specifically, these analyses will not capture expression changes that occur only at the level of protein expression or through alterations in post-translational modification. Similarly, these analyses cannot provide information regarding epigenetic mechanisms, such as DNA methylation, histone modifications, or chromatin accessibility, which may be contributing to the regulation of dystrophin expression and therapeutic responsiveness in dystrophic muscle.

miR-31 upregulation is further enhanced in aged dystrophic muscle

Previous work has shown that miRNAs can repress dystrophin expression, which can limit the effectiveness of exon skipping therapies in *mdx* mice treated at the adult stage.[80,81] The most well-characterized example, miR-31-5p, is highly upregulated in dystrophic muscle.[48–50,80,82] To investigate the role of miRNAs in stage-specific PPMO-treatment efficacy, we measured the expression of miR-31-5p in all treatment groups. Two additional miRNAs, miR-34c-5p and miR-206-3p, were included as they are known to be differentially expressed in dystrophic muscle but are not expected to regulate dystrophin expression per se.[48–50,82,83] miR-31-5p was found to be highly elevated in adult *mdx* muscle (~60-fold, $p < 0.05$) and was not corrected by PPMO treatment (Figure 7A). miR-31-5p upregulation was more pronounced in aged *mdx* muscle (~120-fold, $p < 0.001$) with PPMO-treatment leading to an even greater increase (~186-fold relative to WT, $p < 0.0001$, Figure 7A). By contrast, miR-34c-5p was upregulated in *mdx* muscle at the adult stage only (~6.6-fold, $p < 0.01$, Figure 7B), consistent with previous reports,[48–50,82] and restored to WT levels by PPMO treatment. miR-206-3p was increased by similar magnitudes (~6-fold, $p < 0.01$, Figure 7C) in dystrophic muscle at both time points. These data suggest that an intensification in miR-31-5p expression in aged dystrophic muscle may be one factor contributing to the limited efficacy of PPMO-mediated exon skipping therapy at this age, although a causal relationship cannot be established from the present data.

DISCUSSION

Advanced therapeutics that can restore dystrophin protein expression in the muscles of patients with DMD are now reaching the clinic, with multiple approvals in the US for exon skipping and microdystrophin gene replacement approaches. Next-generation exon skipping approaches are on the horizon, which will likely offer improved dystrophin restoration potential. These developments bring into focus the question of treatment timing.

The data presented herein support the idea that exon skipping treatments should be initiated early in order to maximize therapeutic

benefit. A single intravenous dose of a PPMO conjugate resulted in high levels of *Dmd* exon 23 skipping and dystrophin protein restoration (~35% of WT levels) after only 2 weeks of treatment in adult mice (Figure 2). This was associated with a reversal of histopathological changes (Figure 3B) and a major shift in the dystrophic muscle transcriptome (Figures 4 and 5), indicative of therapeutic success. By contrast, three intravenous doses (2.4-times the total dose administered at the adult stage) resulted in more modest dystrophin restoration (~8%) (Figure 2), with negligible histopathological or transcriptomic restoration (Figures 3, 4, and 5). Together, these data show that the timing of exon skipping treatment is an important determinant of treatment efficacy.

Notably, all PPMOs used in this study were administered via tail vein injection in order to model the systemic infusions of ASOs used clinically in patients with DMD. Alternative delivery routes (e.g., intramuscular or intra-arterial) may result in distinct drug exposure and biodistribution profiles that could influence the relationship between treatment timing and therapeutic efficacy observed following intravenous administration.

The relative lack of therapeutic effectiveness may be a consequence of a number of factors. Firstly, reduced treatment efficacy may be the result of an impairment in drug delivery. Alterations in muscle vasculature may contribute to impaired drug exposure in advanced dystrophic muscle. However, mean RNA level exon skipping was ~44% in aged muscle, suggesting that impaired delivery alone is insufficient to explain the marked reduction in dystrophin protein restoration observed at this disease stage. Furthermore, quantification of the PMO content of muscle tissues revealed a high level of drug accumulation in the aged treated-*mdx* animals (Figure 2D). This is consistent with the drug accumulation following three sequential doses, a higher cumulative total dose, and reduced loss from muscle turnover, as expected. Importantly, this finding suggests that delivery (at least to bulk muscle) is not impaired in aged *mdx* TA muscles.

The muscles of aged *mdx* mice exhibit a greater degree of fibro-fatty degeneration when compared with the adult stage, which resembles the pathology observed in patients with DMD.[69] However, inefficient delivery alone is insufficient to explain all of the data. Specifically, the levels of PPMO-mediated protein restoration were ~4 times lower in aged vs. adult treated animals, while the level of PPMO-mediated exon skipping was only ~1.8 times lower (Figure 2). Indeed, the level of *Dmd* exon 23 skipping in the aged PPMO-treated animals was still relatively high (at 44%), indicative of successful drug delivery and target engagement (although still at a reduced level relative to the adult PPMO-treated mice) (Figure 2).

Secondly, it is possible that the relatively modest dystrophin restoration achieved in aged *mdx* animals may be insufficient to drive meaningful transcriptomic correction in the context of advance disease. In this scenario, dystrophin restoration may occur (at least to some extent) within a limited population of remaining myofibers but remain inadequate to overcome established fibro-fatty degeneration and diminished regenerative capacity (i.e., muscle quality). As a result, molecular correction at the level of individual myofibers may not necessarily translate into broader tissue-level transcriptomic improvement. The lack of transcriptomic response to PPMO treatment in aged *mdx* animals despite detectable exon skipping and ~8% dystrophin protein restoration supports this notion (Figures 2, 4, and 5). These findings raise the possibility that the relationship between dystrophin protein restoration and therapeutic benefit may be influenced by the extent of existing tissue pathology (and therefore, disease stage).

Lastly, it is possible that the aged dystrophic environment is inherently resistant to dystrophin restoration as a consequence of molecular and cellular pathologies. Such concepts have been reported previously, whereby dystrophic muscle is similarly refractory to therapeutic correction, including impaired AAV transgene expression in the post-regeneration environment,[84] direct oxidative damage to the *Dmd* transcript itself,[85] and suppression of dystrophin protein expression via miRNAs.[80,81] Indeed, we observed that miR-31-5p is elevated in adult dystrophic muscle and further increased in aged dystrophic muscle (Figure 7). miR-31-5p was previously shown to be highly upregulated in dystrophic muscle[49,50,82] to directly repress dystrophin, and its inhibition can enhance dystrophin expression following exon skipping therapy.[80,86] It is tempting to speculate that elevated miR-31-5p expression may be contributing to reduced therapeutic efficacy in aged dystrophic muscle and that this may be reversible using an anti-miRNA oligonucleotide approach. However, it is likely that a confluence of factors (e.g., multiple miRNAs, RNA-binding proteins, oxidative stress, gene expression changes and cellular senescence, and AKT/PI3K/mTOR axis perturbation) may act synergistically to limit exon skipping treatment effectiveness in aged dystrophic muscle.

A limitation of this study is that distinct dosing regimens were used for adult and aged treated-*mdx* mice. As such, direct quantitative comparisons should be interpreted with appropriate caution. However, given the multiple dosing (and higher overall dose) paradigm utilized for aged animals, greater overall drug exposure would be expected in this treatment group, which should, if anything, favor increased exon skipping efficacy. The observation of reduced therapeutic response in aged muscle despite this increased drug exposure supports the conclusion that disease stage significantly influences treatment responsiveness. This study was designed to enable deep transcriptomic characterization within a single muscle and therefore, did not include a systemic comparison of exon skipping activity or transcriptomic correction across multiple other muscle tissues. Previous studies using the same, or related, PPMO conjugates have demonstrated widespread exon skipping and dystrophin restoration activity across multiple muscle groups in adult *mdx* mice (including in the heart and the diaphragm),[31–34,49,50,52] supporting broad tissue activity in younger animals. However, whether such tissue-wide efficacy is maintained in aged dystrophic muscle remains to be determined. In addition, we did not assess whole-animal functional outcomes in this study beyond muscle histopathological analysis, which

would have been of interest for contextualizing the transcriptomic findings. Similarly, phenotypic correction can be assessed through circulating biomarkers such as creatine kinase and serum miRNAs.[87] While circulating biomarkers are informative for assessing the response to PPMO therapy in *mdx* mice at the adult stage, as we have reported previously,[33,50,64] their utility in aged animals is limited due to the well-established age-dependent decline in their levels.[50,67,69] As serum samples were not collected in the present study, we were unable to evaluate these measures. However, their usefulness in assessing the therapeutic response to PPMO therapy in the late-stage dystrophic pathology setting is likely to be very limited.

Importantly, an inherent limitation of bulk RNA-seq analysis is the inability to resolve cell-type-specific transcriptomic changes. As such, the gene expression alteration reported here necessarily represent averaged effects across multiple cellular populations within dystrophic muscle, including myofibres, satellite cells, inflammatory cells, FAPs, motor neurons, and vascular endothelial cells. Future studies utilizing single cell sequencing, single nucleus sequencing, and/or spatial transcriptomics techniques may provide further insights into whether cellular composition and cell-type-specific gene expression profiles contribute to the underlying age-dependent reduction in exon skipping efficacy observed in dystrophic muscle.

Importantly, early therapeutic intervention in the case of spinal muscular atrophy (SMA) has proven critical for therapeutic success.[88] Such a benefit of early intervention in patients with DMD remains to be demonstrated. Furthermore, an ongoing challenge in the treatment/management of DMD is the problem of diagnostic delay (the average age at diagnosis is 5 years, with an average time from first signs to diagnostic confirmation of 2.2 years),[89] which hampers efforts toward early initiation of therapy. This issue highlights a need for newborn screening programs for early diagnosis of DMD.[90] The approval of the Elevidys microdystrophin gene therapy (which is potentially applicable to the majority of patients with DMD) strengthens the case for newborn screening, although there are important safety concerns related to this treatment following several instances of fatal acute liver failure in patients with non-ambulatory DMD.[29,91]

Exon skipping therapies may also be implemented as part of a combination therapy. In this manner, exon skipping therapy could be initiated in young DMD boys with the aim of maintaining their muscle function until such an age that treatment with a gene therapy may be safe and appropriate. Indeed, preclinical evidence is supportive of a synergistic effect of combined PMO-mediated exon skipping with either AAV-U7-mediated exon skipping or AAV-microdystrophin gene therapy.[92]

This study adds to the discourse concerning the appropriate time to start exon skipping therapy in patients with DMD. Evidence from the preclinical data presented herein supports the notion of initiating exon skipping therapy at an early age, before the establishment of advanced tissue pathology.

MATERIALS AND METHODS

Animals

Male WT C57 (C57BL/10) and dystrophic *mdx* (C57BL/10ScSn-Dmd^{mdx}/J) mice (both $n = 3$) were harvested at either of two ages: adult (14 weeks) or aged (80 weeks). PPMO conjugates consisting of a PMO (5′-GGCCAAACCTCGGCTTACCTGAAAT, Gene Tools, Philomath, OR, USA) conjugated by amide linkage to Pip6a (Ac-RXRRBRRXRYQFLIRXRBRXRB-OH, X = aminohexanoyl and B = β-Alanine) were prepared in sterile saline, as described previously.[31] All animals were injected via the tail vein under isoflurane anesthesia. All mice were sacrificed by escalating CO_2 concentration, and death was confirmed by cervical dislocation. TA muscles were macrodissected immediately postmortem, mounted onto cork discs using opticla cutting temperature (OCT) media, and flash frozen in liquid nitrogen-cooled isopentane. Animal experimentation was carried out in accordance with the Animals (Scientific Procedures) Act 1986, and all procedures were approved by the UK home office.

RNA preparation

RNA was extracted from 10 mg of TA tissue using the Maxwell 16 RSC instrument with the Maxwell RSC Simply RNA Tissue kit (both Promega, Southampton, UK), which includes a rigorous DNase step. RNA concentration and quality were assessed by Nanodrop spectrophotometry to measure absorbance at 260 nm and the 260/280 and 260/230 ratios. RNA integrity assessed by Bioanalyzer (Eukaryote RNA Pico chip) (Agilent, Didcot, UK). Contamination of RNA samples with genomic DNA was assessed by qPCR (with or without reverse transcription) using a TaqMan assay, which amplifies *Actb* mRNA/genomic DNA (Mm00607939_s1). RNA samples (1 μg each) were depleted of rRNA using the NEBNext rRNA Depletion Kit (human/mouse/rat) (New England Biolabs, Hitchin, UK) according to the manufacturer's instructions with minor modifications (reaction volumes were scaled to accommodate sample RNA concentrations). An rRNA-depleted total RNA library approach was used to enable quantification of non-polyadenylated and partially processed transcripts and to minimize the 3' coverage bias commonly associated with poly(A)-enrichment protocols, which is particularly relevant for very large transcripts such as *Dmd*. rRNA-depleted RNA samples were eluted in 10 μL of nuclease-free water, and 8 μL was retained for further analysis. The concentrations of rRNA-depleted RNA samples were determined by RNA fluorometry by using the Quantus instrument and appropriate reagents (QunatiFluor RNA System) (both Promega). RNA quality control is summarized in Figure S1.

RNA-seq

RNA-seq was performed as a service at Karolinsk Institutet on the Illumina NextSeq 500 platform. Sequencing reads were processed using a custom in-house pipeline. In brief, the suitability of FASTQ files for analyses was assessed by using FastQC (v0.10.1) and MultiQC (v0.7).[93] Reads were trimmed using Trim_Galore (v0.3.1) and aligned to the mouse genome (GRCm38) by using HISAT2

(v.2.1.0).[94] Alignment files were processed using Samtools (v1.3).[95] Reads were counted using HTSeq (v0.9.1) against the v96 GTF file obtained from Ensembl.[96] Differential expression was assessed using DESeq2 (v1.28.1). Differential exon expression usage was assessed using DEXSeq (v3.8).[75] GO analysis was performed using a combination of g:Profiler and REVIGO.[97,98] Raw RNA-seq data are deposited in NCBI SRA under BioProject: PRJNA1384431. Counts data are provided in Table S3.

Exon skipping RT-qPCR

Exon skipping was confirmed by RT-qPCR to measure the levels of *Dmd* transcripts lacking exon 23. Briefly, reverse transcription through a random priming strategy was performed on 1 μg total RNA by using the High Capacity cDNA Kit (Thermo Fisher Scientific, Rugby, UK) according to manufacturer's instructions. Levels of *Dmd* Δ23 transcripts were determined through primer/probe assays (Integrated DNA Technologies, Leuven, Belgium) spanning the exon 23-24 boundary (to measure unskipped *Dmd* transcripts) and spanning the exon 20-21 boundary (to measure total *Dmd* transcripts) (Table S1). Probes were HEX- and FAM-labeled, respectively, and run in singleplex format. qPCR was performed on a StepOne Plus real-time PCR Thermocycler using TaqMan Gene Expression Master Mix (both Thermo Fisher Scientific) and 25 ng cDNA template was added per reaction. The percentage of *Dmd* exon 23 skipping was determined by calculating (1 − the ratio of unskipped:total *Dmd* transcripts) × 100%.

Dystrophin western blot

For dystrophin protein quantification, 8 μm cryosections (∼80) were prepared from the mid-belly of TA muscle and samples lysed in lysis buffer (50 mM Tris pH 8, 150 mM NaCl, 1% NP-40, 0.5% sodium deoxycholate, 10% sodium dodecyl sulfate, and protease inhibitors). Protein lysates were incubated at 100°C for 3 min and then centrifuged at 14,000 ×*g* for 10 min at 4°C to pellet debris and supernatants transferred to fresh tubes. Equal amounts of protein lysate (20 μg) were separated on a 3%–8% tris-acetate gel (Life Technologies), electrotransferred to a polyvinylidene fluoride (PVDF) membrane, and probed with monoclonal anti-dystrophin (NCL-DYS1, Leica Biosystems, Lincoln, NE, USA) and anti-vinculin (hVIN-1 V9131, Sigma, Dorset, UK) primary antibodies). Secondary antibody fluorescence was quantified using the Odyssey imaging system (IRDye 800CW goat anti-mouse, LiCOR Biosystems). To quantify dystrophin expression, the dystrophin to vinculin ratio for each PPMO-treated sample was compared with a dilution series of C57Bl/10 lysate (diluted in *mdx* lysate to maintain consistent vinculin expression levels between standards), as described previously.[52]

RT-ddPCR

Absolute quantification of RNA transcript abundance across the *Dmd* gene was determined by RT-ddPCR. Reactions were prepared using QX200 ddPCR EvaGreen Supermix (Bio-Rad, Watford, UK) and 10 pmol of each primer was used per 20 μL reaction. cDNA (generated as described earlier) was diluted 1:4 in nuclease-free water, and 1 μL of diluted cDNA was used per reaction. Droplets were prepared using the QX200 Droplet Generator and QX200 Droplet Generation Oil for EvaGreen (both Bio-Rad). Subsequently, droplets were transferred to a 96 well plate and the plate sealed using the PX1 PCR Plate Sealer (Bio-Rad). PCR was performed by using the following cycling conditions: 95°C enzyme activation step for 5 min followed by 40 cycles of a two-step cycling protocol (96°C for 30 s and 58°C for 1 min). The ramp rate between these steps was set to 2°C per second. After thermal cycling, the plate was analyzed using a QX600 droplet reader (Bio-Rad), according to the manufacturer's instructions. Positive and negative droplet populations were distinguished using assay-specific fluorescence thresholds, and absolute transcript abundance was calculated using Poisson statistics. Results were reported as copies/μL. RT-ddPCR assays were adapted from Spitali et al.[74] and are listed in Table S1.

PMO quantification

The PMO content of muscle lysates was determined by using an ELISA-like workflow, as described previously.[99] Briefly, tissue lysates were prepared by homogenization in RIPA buffer containing proteinase K (2 mg/mL), followed by overnight incubation at 55°C and then centrifugation at 13,800 ×*g* for 15 min. Cleared lysates were diluted in PMO dilution buffer (10 mM Tris-HCl, 1 mM EDTA, pH 8, 0.1% w/v Triton X-100) and hybridized with a biotin- and digoxigenin-labeled PMO capture probe in hybridization buffer (10 mM Tris-HCl, 1 mM EDTA, pH 8, 1 M NaCl, 0.1% w/v Triton X-100). The probe sequence was 5′-BIO-A*T*T*T*C*A*G*G TAAGCCGAGG*T*T*T*G*G*C*C-DIG-3′, where phosphorothioate backbone linkages are indicated by asterisks (*). Hybridized complexes were captured on NeutrAvidin-coated 96 well plates (Thermo Fisher Scientific), and unbound probe was removed by digestion with micrococcal nuclease (New England Biolabs). Bound probe was detected using alkaline phosphatase-conjugated anti-digoxigenin antibody (Roche Diagnostics, Burgess Hill, UK) and the AttoPhos AP fluorescent substrate system (Promega). Fluorescence was measured using a CLARIOstar microplate reader (BMG LABTECH, Aylesbury, UK) with excitation at 444 nm and emission at 555 nm. PMO concentrations were determined by interpolation from a standard curve generated using serial dilutions of PMO standards (25–2,000 pM) and corrected for sample dilution factors. PMO content was normalized to total protein content determined by bicinchoninic acid assay (BCA) assay.

Histology and immunofluorescence

8 μm sections of TA muscles were analyzed at the Histology Core, Kennedy Institute of Rheumatology, University of Oxford.

miRNA RT-qPCR

miRNAs were quantified using the small RNA TaqMan RT-qPCR method using miRNA-specific stem loop reverse transcription primers. Details of miRNA assays are listed in Table S2. Reverse transcription was performed using the TaqMan MicroRNA Reverse Transcription Kit (Thermo Fisher Scientific) with and 20 ng of input total RNA per reaction. cDNA was amplified through a StepOne Plus real-time PCR thermocycler with TaqMan Gene Expression Master

Mix (both Thermo Fisher Scientific) by using universal cycling conditions: 95°C for 10 min, followed by 40 cycles of 95°C for 15 s, and 60°C for 1 min. All samples were analyzed in duplicate. Relative quantification was performed using the Pfaffl method[100] and miRNA of interest expression normalized to miR-16-5p.

Statistics

Statistical analysis was performed in GraphPad Prism (v10.2.3) (GraphPad Software Inc., San Diego, California, USA) on $\log_2$-transformed values using an unpaired Student's *t* test for comparisons between two group and an ordinary one-way analysis of variance (-ANOVA) with Tukey's post hoc test for inter-group comparisons when more than two groups were being compared.

DATA AND CODE AVAILABILITY

Raw data have been deposited in NCBI sequence read archive (SRA) under BioProject: PRJNA1384431. Processed counts data are included as a supplemental information file. All other data are contained in the manuscript.

ACKNOWLEDGMENTS

This work was supported by a grant from the UK Medical Research Council (MR/X008029/1) awarded to M.J.A.W. and T.C.R. S.S. was funded by an MRC Confidence-in-Concept grant awarded to M.J.A.W. and T.C.R. T.L.E.v.W. was supported by a doctoral studentship from Muscular Dystrophy UK (MDUK) awarded to M.J.A.W. and T.C.R.

AUTHOR CONTRIBUTIONS

T.C.R., S.E.A., and M.J.A.W. conceived the study; T.C.R., S.E.A., and M.J.A.W. supervised the work; S.S., J.H., T.L.E.v.W., A.M.L.C.-S., Y.J., and K.K. performed experimentation; T.C.R. wrote the first draft of the manuscript. All authors contributed to and approved the final version of the manuscript.

DECLARATION OF INTERESTS

M.J.A.W. is a founder, advisor, and shareholder in PepGen Ltd, a company which aims to commercialize PPMO technologies similar to those utilized in this manuscript. M.J.A.W. has filed patents related to PPMO technology.

SUPPLEMENTAL INFORMATION

Supplemental information can be found online at https://doi.org/10.1016/j.omtn.2026.103017.

REFERENCES

1. Hoffman, E.P., Brown, R.H., and Kunkel, L.M. (1987). Dystrophin: The protein product of the duchenne muscular dystrophy locus. Cell *51*, 919–928. https://doi.org/10.1016/0092-8674(87)90579-4.
2. Petrof, B.J., Shrager, J.B., Stedman, H.H., Kelly, A.M., and Sweeney, H.L. (1993). Dystrophin protects the sarcolemma from stresses developed during muscle contraction. Proc. Natl. Acad. Sci. USA *90*, 3710–3714. https://doi.org/10.1073/pnas.90.8.3710.
3. Moriuchi, T., Kagawa, N., Mukoyama, M., and Hizawa, K. (1993). Autopsy analyses of the muscular dystrophies. Tokushima J. Exp. Med. *40*, 83–93.
4. Chiang, D.Y., Allen, H.D., Kim, J.J., Valdes, S.O., Wang, Y., Pignatelli, R.H., Lotze, T.E., and Miyake, C.Y. (2016). Relation of Cardiac Dysfunction to Rhythm Abnormalities in Patients With Duchenne or Becker Muscular Dystrophies. Am. J. Cardiol. *117*, 1349–1354. https://doi.org/10.1016/j.amjcard.2016.01.031.
5. Ishikawa, Y., Miura, T., Ishikawa, Y., Aoyagi, T., Ogata, H., Hamada, S., and Minami, R. (2011). Duchenne muscular dystrophy: Survival by cardio-respiratory interventions. Neuromuscul. Disord. *21*, 47–51. https://doi.org/10.1016/j.nmd.2010.09.006.
6. Birnkrant, D.J., Bushby, K., Bann, C.M., Apkon, S.D., Blackwell, A., Brumbaugh, D., Case, L.E., Clemens, P.R., Hadjiyannakis, S., Pandya, S., et al. (2018). Diagnosis and management of Duchenne muscular dystrophy, part 1: diagnosis, and neuromuscular, rehabilitation, endocrine, and gastrointestinal and nutritional management. Lancet Neurol. *17*, 251–267. https://doi.org/10.1016/S1474-4422(18)30024-3.
7. Birnkrant, D.J., Bushby, K., Bann, C.M., Alman, B.A., Apkon, S.D., Blackwell, A., Case, L.E., Cripe, L., Hadjiyannakis, S., Olson, A.K., et al. (2018). Diagnosis and management of Duchenne muscular dystrophy, part 2: respiratory, cardiac, bone health, and orthopaedic management. Lancet Neurol. *17*, 347–361. https://doi.org/10.1016/S1474-4422(18)30025-5.
8. Birnkrant, D.J., Bushby, K., Bann, C.M., Apkon, S.D., Blackwell, A., Colvin, M.K., Cripe, L., Herron, A.R., Kennedy, A., Kinnett, K., et al. (2018). Diagnosis and management of Duchenne muscular dystrophy, part 3: primary care, emergency management, psychosocial care, and transitions of care across the lifespan. Lancet Neurol. *17*, 445–455. https://doi.org/10.1016/S1474-4422(18)30026-7.
9. Ricotti, V., Ridout, D.A., Scott, E., Quinlivan, R., Robb, S.A., Manzur, A.Y., and Muntoni, F.; NorthStar Clinical Network (2013). Long-term benefits and adverse effects of intermittent versus daily glucocorticoids in boys with Duchenne muscular dystrophy. J. Neurol. Neurosurg. Psychiatry *84*, 698–705. https://doi.org/10.1136/jnnp-2012-303902.
10. Kim, S., Campbell, K.A., Fox, D.J., Matthews, D.J., and Valdez, R.; MD STARnet (2015). Corticosteroid Treatments in Males With Duchenne Muscular Dystrophy: Treatment Duration and Time to Loss of Ambulation. J. Child Neurol. *30*, 1275–1280. https://doi.org/10.1177/0883073814558120.
11. Kourakis, S., Timpani, C.A., Campelj, D.G., Hafner, P., Gueven, N., Fischer, D., and Rybalka, E. (2021). Standard of care versus new-wave corticosteroids in the treatment of Duchenne muscular dystrophy: Can we do better? Orphanet J. Rare Dis. *16*, 117. https://doi.org/10.1186/s13023-021-01758-9.
12. Roberts, T.C., Wood, M.J.A., and Davies, K.E. (2023). Therapeutic approaches for Duchenne muscular dystrophy. Nat. Rev. Drug Discov. *22*, 917–934. https://doi.org/10.1038/s41573-023-00775-6.
13. Hanson, B., Wood, M.J.A., and Roberts, T.C. (2021). Molecular correction of Duchenne muscular dystrophy by splice modulation and gene editing. RNA Biol. *18*, 1048–1062. https://doi.org/10.1080/15476286.2021.1874161.
14. Roberts, T.C., Langer, R., and Wood, M.J.A. (2020). Advances in oligonucleotide drug delivery. Nat. Rev. Drug Discov. *19*, 673–694. https://doi.org/10.1038/s41573-020-0075-7.
15. Bladen, C.L., Salgado, D., Monges, S., Foncuberta, M.E., Kekou, K., Kosma, K., Dawkins, H., Lamont, L., Roy, A.J., Chamova, T., et al. (2015). The TREAT-NMD DMD global database: Analysis of more than 7,000 duchenne muscular dystrophy mutations. Hum. Mutat. *36*, 395–402. https://doi.org/10.1002/humu.22758.
16. Chwalenia, K., Wood, M.J.A., and Roberts, T.C. (2025). Progress and prospects in antisense oligonucleotide-mediated exon skipping therapies for Duchenne muscular dystrophy. J. Muscle Res. Cell Motil. *46*, 293–300. https://doi.org/10.1007/s10974-024-09688-2.
17. Charleston, J.S., Schnell, F.J., Dworzak, J., Donoghue, C., Lewis, S., Chen, L., Young, G.D., Milici, A.J., Voss, J., DeAlwis, U., et al. (2018). Eteplirsen treatment for Duchenne muscular dystrophy: Exon skipping and dystrophin production. Neurology *90*, e2146–e2154. https://doi.org/10.1212/WNL.0000000000005680.
18. Shirley, M. (2021). Casimersen: First Approval. Drugs *81*, 875–879. https://doi.org/10.1007/s40265-021-01512-2.
19. Komaki, H., Nagata, T., Saito, T., Masuda, S., Takeshita, E., Sasaki, M., Tachimori, H., Nakamura, H., Aoki, Y., and Takeda, S. (2018). Systemic administration of the antisense oligonucleotide NS-065/NCNP-01 for skipping of exon 53 in patients with Duchenne muscular dystrophy. Sci. Transl. Med. *10*, eaan0713. https://doi.org/10.1126/scitranslmed.aan0713.
20. Servais, L., Mercuri, E., Straub, V., Guglieri, M., Seferian, A.M., Scoto, M., Leone, D., Koenig, E., Khan, N., Dugar, A., et al. (2022). Long-Term Safety and Efficacy Data of Golodirsen in Ambulatory Patients with Duchenne Muscular Dystrophy Amenable to Exon 53 Skipping: A First-in-human, Multicenter, Two-Part, Open-Label, Phase 1/2 Trial. Nucleic Acid Ther. *32*, 29–39. https://doi.org/10.1089/nat.2021.0043.

21. Muntoni, F., Fletcher, S., and Wilton, S. (2017). Response to "Railroading at the FDA". Nat. Biotechnol. *35*, 207–209. https://doi.org/10.1038/nbt.3819.

22. Aartsma-Rus, A., and Krieg, A.M. (2017). FDA Approves Eteplirsen for Duchenne Muscular Dystrophy: The Next Chapter in the Eteplirsen Saga. Nucleic Acid Ther. *27*, 1–3. https://doi.org/10.1089/nat.2016.0657.

23. Dowling, J.J. (2016). Eteplirsen therapy for Duchenne muscular dystrophy: skipping to the front of the line. Nat. Rev. Neurol. *12*, 675–676. https://doi.org/10.1038/nrneurol.2016.180.

24. FDA Briefing Document. Peripheral and Central Nervous System Drugs Advisory Committee Meeting, January 22, 2016, NDA 206488, Eteplirsen. 2016. https://www.fda.gov/files/advisory%20committees/published/FDA-Briefing-Information-for-the-January-22–2016-Meeting-of-the-Peripheral-and-Central-Nervous-System-Drugs-Advisory-Committee.pdf.

25. (2016). Railroading at the FDA. Nat. Biotechnol. *34*, 1078. https://doi.org/10.1038/nbt.3733.

26. Mendell, J.R., Khan, N., Sha, N., Eliopoulos, H., McDonald, C.M., Goemans, N., Mercuri, E., Lowes, L.P., and Alfano, L.N.; Eteplirsen Study Group (2021). Comparison of Long-term Ambulatory Function in Patients with Duchenne Muscular Dystrophy Treated with Eteplirsen and Matched Natural History Controls. J. Neuromuscul. Dis. *8*, 469–479. https://doi.org/10.3233/JND-200548.

27. Hoy, S.M. (2023). Delandistrogene Moxeparvovec: First Approval. Drugs *83*, 1323–1329. https://doi.org/10.1007/s40265-023-01929-x.

28. Mendell, J.R., Muntoni, F., McDonald, C.M., Mercuri, E.M., Ciafaloni, E., Komaki, H., Leon-Astudillo, C., Nascimento, A., Proud, C., Schara-Schmidt, U., et al. (2024). AAV gene therapy for Duchenne muscular dystrophy: the EMBARK phase 3 randomized trial. Nat. Med. *31*, 332–341. https://doi.org/10.1038/s41591-024-03304-z.

29. Chwalenia, K., Feng, V.-Y., Hemmer, N., Friedrichsen, H.J., Vorobieva, I., Wood, M.J.A., and Roberts, T.C. (2025). AAV microdystrophin gene replacement therapy for Duchenne muscular dystrophy: progress and prospects. Gene Ther. *32*, 447–461. https://doi.org/10.1038/s41434-025-00561-6.

30. Wu, B., Lu, P., Benrashid, E., Malik, S., Ashar, J., Doran, T.J., and Lu, Q.L. (2010). Dose-dependent restoration of dystrophin expression in cardiac muscle of dystrophic mice by systemically delivered morpholino. Gene Ther. *17*, 132–140. https://doi.org/10.1038/gt.2009.120.

31. Betts, C., Saleh, A.F., Arzumanov, A.A., Hammond, S.M., Godfrey, C., Coursindel, T., Gait, M.J., and Wood, M.J. (2012). Pip6-PMO, A New Generation of Peptide-oligonucleotide Conjugates With Improved Cardiac Exon Skipping Activity for DMD Treatment. Mol. Ther. Nucleic Acids *1*, e38. https://doi.org/10.1038/mtna.2012.30.

32. Betts, C.A., Saleh, A.F., Carr, C.A., Hammond, S.M., Coenen-Stass, A.M.L., Godfrey, C., McClorey, G., Varela, M.A., Roberts, T.C., Clarke, K., et al. (2015). Prevention of exercised induced cardiomyopathy following Pip-PMO treatment in dystrophic mdx mice. Sci. Rep. *5*, 8986. https://doi.org/10.1038/srep08986.

33. Roberts, T.C., Godfrey, C., McClorey, G., Vader, P., Briggs, D., Gardiner, C., Aoki, Y., Sargent, I., Morgan, J.E., and Wood, M.J.A. (2013). Extracellular microRNAs are dynamic non-vesicular biomarkers of muscle turnover. Nucl. Acids Res. *41*, 9500–9513. https://doi.org/10.1093/nar/gkt724.

34. Yin, H., Saleh, A.F., Betts, C., Camelliti, P., Seow, Y., Ashraf, S., Arzumanov, A., Hammond, S., Merritt, T., Gait, M.J., and Wood, M.J. (2011). Pip5 transduction peptides direct high efficiency oligonucleotide-mediated dystrophin exon skipping in heart and phenotypic correction in mdx mice. Mol. Ther. *19*, 1295–1303. https://doi.org/10.1038/mt.2011.79.

35. Ezzat, K., Aoki, Y., Koo, T., McClorey, G., Benner, L., Coenen-Stass, A., O'Donovan, L., Lehto, T., Garcia-Guerra, A., Nordin, J., et al. (2015). Self-Assembly into Nanoparticles Is Essential for Receptor Mediated Uptake of Therapeutic Antisense Oligonucleotides. Nano Lett. *15*, 4364–4373. https://doi.org/10.1021/acs.nanolett.5b00490.

36. Lehto, T., Castillo Alvarez, A., Gauck, S., Gait, M.J., Coursindel, T., Wood, M.J.A., Lebleu, B., and Boisguerin, P. (2014). Cellular trafficking determines the exon skipping activity of Pip6a-PMO in mdx skeletal and cardiac muscle cells. Nucleic Acids Res. *42*, 3207–3217. https://doi.org/10.1093/nar/gkt1220.

37. Kreher, N., Kumar, A., Hicks, A., Peddigari, S., Li, X., Pathak, A., Kheirabadi, M., Kamer, K., Estrella, N., Dougherty, P., et al. (2022). P.194 Development of a novel, EEV-Conjugated PMO for Duchenne muscular dystrophy. Neuromuscul. Disord. *32*, S126. https://doi.org/10.1016/j.nmd.2022.07.349.

38. PepGen Reports Positive Data from Phase 1 Trial of PGN-EDO51 for the Treatment of Duchenne Muscular Dystrophy | PepGen https://investors.pepgen.com/news-releases/news-release-details/pepgen-reports-positive-data-phase-1-trial-pgn-edo51-treatment/.

39. Moulton, H.M., and Moulton, J.D. (2010). Morpholinos and their peptide conjugates: Therapeutic promise and challenge for Duchenne muscular dystrophy. Biochim. Biophys. Acta *1798*, 2296–2303. https://doi.org/10.1016/j.bbamem.2010.02.012.

40. Amantana, A., Moulton, H.M., Cate, M.L., Reddy, M.T., Whitehead, T., Hassinger, J.N., Youngblood, D.S., and Iversen, P.L. (2007). Pharmacokinetics, biodistribution, stability and toxicity of a cell-penetrating peptide-morpholino oligomer conjugate. Bioconjug. Chem. *18*, 1325–1331. https://doi.org/10.1021/bc070060v.

41. Community Letter: Update SRP-5051 Program | Sarepta Therapeutics https://www.sarepta.com/community-letter-update-srp-5051-program.

42. Sarepta Therapeutics Announces Third Quarter 2024 Financial Results and Recent Corporate Developments | Sarepta Therapeutics, Inc. https://investorrelations.sarepta.com/news-releases/news-release-details/sarepta-therapeutics-announces-third-quarter-2024-financial.

43. Dystrophy, P.P.M. (2025). PepGen Announces Discontinuation of PGN-EDO51 Development (Parent Project Muscular Dystrophy). https://www.parentprojectmd.org/pepgen-announces-discontinuation-of-pgn-edo51-development/.

44. Avidity Biosciences Announces Phase 1/2 EXPLORE44^{TM} Trial of AOC 1044 for Duchenne Muscular Dystrophy Mutations Amenable to Exon 44 Skipping Investors | Avidity Biosciences. https://aviditybiosciences.investorroom.com/2022-10-11-Avidity-Biosciences-Announces-Phase-1-2-EXPLORE44-TM-Trial-of-AOC-1044-for-Duchenne-Muscular-Dystrophy-Mutations-Amenable-to-Exon-44-Skipping.

45. Desjardins, C.A., Yao, M., Hall, J., O'Donnell, E., Venkatesan, R., Spring, S., Wen, A., Hsia, N., Shen, P., Russo, R., et al. (2022). Enhanced exon skipping and prolonged dystrophin restoration achieved by TfR1-targeted delivery of antisense oligonucleotide using FORCE conjugation in mdx mice. Nucleic Acids Res. *50*, 11401–11414. https://doi.org/10.1093/nar/gkac641.

46. Bulfield, G., Siller, W.G., Wight, P.A., and Moore, K.J. (1984). X chromosome-linked muscular dystrophy (mdx) in the mouse. Proc. Natl. Acad. Sci. USA *81*, 1189–1192. https://doi.org/10.1073/pnas.81.4.1189.

47. Sicinski, P., Geng, Y., Ryder-Cook, A.S., Barnard, E.A., Darlison, M.G., and Barnard, P.J. (1989). The molecular basis of muscular dystrophy in the mdx mouse: a point mutation. Science *244*, 1578–1580. https://doi.org/10.1126/science.2662404.

48. Roberts, T.C., Johansson, H.J., McClorey, G., Godfrey, C., Blomberg, K.E.M., Coursindel, T., Gait, M.J., Smith, C.I.E., Lehtiö, J., El Andaloussi, S., and Wood, M.J.A. (2015). Multi-level omics analysis in a murine model of dystrophin loss and therapeutic restoration. Hum. Mol. Genet. *24*, 6756–6768. https://doi.org/10.1093/hmg/ddv381.

49. Roberts, T.C., Blomberg, K.E.M., McClorey, G., Andaloussi, S.E., Godfrey, C., Betts, C., Coursindel, T., Gait, M.J., Edvard Smith, C., and Wood, M.J. (2012). Expression Analysis in Multiple Muscle Groups and Serum Reveals Complexity in the MicroRNA Transcriptome of the mdx Mouse with Implications for Therapy. Mol. Ther. Nucleic Acids *1*, e39. https://doi.org/10.1038/mtna.2012.26.

50. Coenen-Stass, A.M.L., Sork, H., Gatto, S., Godfrey, C., Bhomra, A., Krjutškov, K., Hart, J.R., Westholm, J.O., O'Donovan, L., Roos, A., et al. (2018). Comprehensive RNA-Sequencing Analysis in Serum and Muscle Reveals Novel Small RNA Signatures with Biomarker Potential for DMD. Mol. Ther. Nucleic Acids *13*, 1–15. https://doi.org/10.1016/j.omtn.2018.08.005.

51. van Westering, T.L.E., Johansson, H.J., Hanson, B., Coenen-Stass, A.M.L., Lomonosova, Y., Tanihata, J., Motohashi, N., Yokota, T., Takeda, S., Lehtiö, J., et al. (2020). Mutation-independent Proteomic Signatures of Pathological Progression in Murine Models of Duchenne Muscular Dystrophy. Mol. Cell. Proteomics *19*, 2047–2068. https://doi.org/10.1074/mcp.RA120.002345.

52. Godfrey, C., Muses, S., McClorey, G., Wells, K.E., Coursindel, T., Terry, R.L., Betts, C., Hammond, S., O'Donovan, L., Hildyard, J., et al. (2015). How much dystrophin is enough: the physiological consequences of different levels of dystrophin in the mdx mouse. Hum. Mol. Genet. *24*, 4225–4237. https://doi.org/10.1093/hmg/ddv155.

53. van den Bergen, J.C., Wokke, B.H., Janson, A.A., van Duinen, S.G., Hulsker, M.A., Ginjaar, H.B., van Deutekom, J.C., Aartsma-Rus, A., Kan, H.E., and Verschuuren, J.J. (2014). Dystrophin levels and clinical severity in Becker muscular dystrophy patients. J. Neurol. Neurosurg. Psychiatry *85*, 747–753. https://doi.org/10.1136/jnnp-2013-306350.

54. Hoffman, E.P., Kunkel, L.M., Angelini, C., Clarke, A., Johnson, M., and Harris, J.B. (1989). Improved diagnosis of Becker muscular dystrophy by dystrophin testing. Neurology *39*, 1011–1017. https://doi.org/10.1212/wnl.39.8.1011.

55. England, S.B., Nicholson, L.V., Johnson, M.A., Forrest, S.M., Love, D.R., Zubrzycka-Gaarn, E.E., Bulman, D.E., Harris, J.B., and Davies, K.E. (1990). Very mild muscular dystrophy associated with the deletion of 46% of dystrophin. Nature *343*, 180–182. https://doi.org/10.1038/343180a0.

56. (2022). Nationwide Children's Announces Restoration of Full-Length Dystrophin Using Dup 2 Gene Therapy Approach (Parent Project Muscular Dystrophy). https://www.parentprojectmd.org/nationwide-childrens-hospital-announces-restoration-of-full-length-dystrophin-using-duplication-2-gene-therapy-approach/.

57. Tasfaout, H., Halbert, C.L., McMillen, T.S., Allen, J.M., Reyes, T.R., Flint, G.V., Grimm, D., Hauschka, S.D., Regnier, M., and Chamberlain, J.S. (2024). Split intein-mediated protein trans-splicing to express large dystrophins. Nature *632*, 192–200. https://doi.org/10.1038/s41586-024-07710-8.

58. Lindley, S.R., Subbaiah, K.C.V., Priyanka, F., Poosala, P., Ma, Y., Jalinous, L., West, J.A., Richardson, W.A., Thomas, T.N., and Anderson, D.M. (2024). Ribozyme-activated mRNA trans-ligation enables large gene delivery to treat muscular dystrophies. Science *386*, 762–767. https://doi.org/10.1126/science.adp8179.

59. Zhou, Y., Zhang, C., Xiao, W., Herzog, R.W., and Han, R. (2024). Systemic delivery of full-length dystrophin in Duchenne muscular dystrophy mice. Nat. Commun. *15*, 6141. https://doi.org/10.1038/s41467-024-50569-6.

60. Counsell, J.R., Asgarian, Z., Meng, J., Ferrer, V., Vink, C.A., Howe, S.J., Waddington, S.N., Thrasher, A.J., Muntoni, F., Morgan, J.E., and Danos, O. (2017). Lentiviral vectors can be used for full-length dystrophin gene therapy. Sci. Rep. *7*, 79. https://doi.org/10.1038/s41598-017-00152-5.

61. Huard, J., Krisky, D., Oligino, T., Marconi, P., Day, C.S., Watkins, S.C., and Glorioso, J.C. (1997). Gene transfer to muscle using herpes simplex virus-based vectors. Neuromuscul. Disord. *7*, 299–313. https://doi.org/10.1016/s0960-8966(97)00054-0.

62. Dudley, R.W.R., Lu, Y., Gilbert, R., Matecki, S., Nalbantoglu, J., Petrof, B.J., and Karpati, G. (2004). Sustained improvement of muscle function one year after full-length dystrophin gene transfer into mdx mice by a gutted helper-dependent adenoviral vector. Hum. Gene Ther. *15*, 145–156. https://doi.org/10.1089/104303404772679959.

63. van Westering, T.L.E., Lomonosova, Y., Coenen-Stass, A.M.L., Betts, C.A., Bhomra, A., Hulsker, M., Clark, L.E., McClorey, G., Aartsma-Rus, A., van Putten, M., et al. (2020). Uniform sarcolemmal dystrophin expression is required to prevent extracellular microRNA release and improve dystrophic pathology. J. Cachexia Sarcopenia Muscle *11*, 578–593. https://doi.org/10.1002/jcsm.12506.

64. Chwalenia, K., Oieni, J., Zemła, J., Lekka, M., Ahlskog, N., Coenen-Stass, A.M.L., McClorey, G., Wood, M.J.A., Lomonosova, Y., and Roberts, T.C. (2022). Exon skipping induces uniform dystrophin rescue with dose-dependent restoration of serum miRNA biomarkers and muscle biophysical properties. Mol. Ther. Nucleic Acids *29*, 955–968. https://doi.org/10.1016/j.omtn.2022.08.033.

65. Hanson, B., Stenler, S., Ahlskog, N., Chwalenia, K., Svrzikapa, N., Coenen-Stass, A.M.L., Weinberg, M.S., Wood, M.J.A., and Roberts, T.C. (2022). Non-uniform dystrophin re-expression after CRISPR-mediated exon excision in the dystrophin/utrophin double-knockout mouse model of DMD. Mol. Ther. Nucleic Acids *30*, 379–397. https://doi.org/10.1016/j.omtn.2022.10.010.

66. Morin, A., Stantzou, A., Petrova, O.N., Hildyard, J., Tensorer, T., Matouk, M., Petkova, M.V., Richard, I., Manoliu, T., Goyenvalle, A., et al. (2023). Dystrophin myonuclear domain restoration governs treatment efficacy in dystrophic muscle. Proc. Natl. Acad. Sci. USA *120*, e2206324120. https://doi.org/10.1073/pnas.2206324120.

67. Chwalenia, K., Feng, V.-Y., Hemmer, N., Hildyard, J.C.W., Roskrow, L.E., Piercy, R.J., Wang, E.T., Aartsma-Rus, A., van Putten, M., Wood, M.J.A., and Roberts, T. (2026). Myonuclear Domain-Associated and Central Nucleation-Dependent Spatial Restriction of Dystrophin Protein Expression. J. Cachexia Sarcopenia Muscle *17*, e70284. https://doi.org/10.1002/jcsm.70284.

68. Coenen-Stass, A.M.L., McClorey, G., Manzano, R., Betts, C.A., Blain, A., Saleh, A.F., Gait, M.J., Lochmüller, H., Wood, M.J.A., and Roberts, T.C. (2015). Identification of novel, therapy-responsive protein biomarkers in a mouse model of Duchenne muscular dystrophy by aptamer-based serum proteomics. Sci. Rep. *5*, 17014. https://doi.org/10.1038/srep17014.

69. Coenen-Stass, A.M.L., Betts, C.A., Lee, Y.F., Mäger, I., Turunen, M.P., El Andaloussi, S., Morgan, J.E., Wood, M.J.A., and Roberts, T.C. (2016). Selective release of muscle-specific, extracellular microRNAs during myogenic differentiation. Hum. Mol. Genet. *25*, 3960–3974. https://doi.org/10.1093/hmg/ddw237.

70. Chamberlain, J.S., Pearlman, J.A., Muzny, D.M., Gibbs, R.A., Ranier, J.E., Caskey, C.T., and Reeves, A.A. (1988). Expression of the murine Duchenne muscular dystrophy gene in muscle and brain. Science *239*, 1416–1418. https://doi.org/10.1126/science.3347839.

71. Haslett, J.N., Sanoudou, D., Kho, A.T., Bennett, R.R., Greenberg, S.A., Kohane, I.S., Beggs, A.H., and Kunkel, L.M. (2002). Gene expression comparison of biopsies from Duchenne muscular dystrophy (DMD) and normal skeletal muscle. Proc. Natl. Acad. Sci. USA *99*, 15000–15005. https://doi.org/10.1073/pnas.192571199.

72. García-Rodríguez, R., Hiller, M., Jiménez-Gracia, L., van der Pal, Z., Balog, J., Adamzek, K., Aartsma-Rus, A., and Spitali, P. (2020). Premature termination codons in the DMD gene cause reduced local mRNA synthesis. Proc. Natl. Acad. Sci. USA *117*, 16456–16464. https://doi.org/10.1073/pnas.1910456117.

73. Tennyson, C.N., Shi, Q., and Worton, R.G. (1996). Stability of the human dystrophin transcript in muscle. Nucleic Acids Res. *24*, 3059–3064. https://doi.org/10.1093/nar/24.15.3059.

74. Spitali, P., van den Bergen, J.C., Verhaart, I.E.C., Wokke, B., Janson, A.A.M., van den Eijnde, R., den Dunnen, J.T., Laros, J.F.J., Verschuuren, J.J.G.M., 't Hoen, P.A.C., and Aartsma-Rus, A. (2013). DMD transcript imbalance determines dystrophin levels. FASEB J. *27*, 4909–4916. https://doi.org/10.1096/fj.13-232025.

75. Anders, S., Reyes, A., and Huber, W. (2012). Detecting differential usage of exons from RNA-seq data. Genome Res. *22*, 2008–2017. https://doi.org/10.1101/gr.133744.111.

76. Bizot, F., Goossens, R., Tensorer, T., Dmitriev, S., Garcia, L., Aartsma-Rus, A., Spitali, P., and Goyenvalle, A. (2022). Histone deacetylase inhibitors improve antisense-mediated exon-skipping efficacy in mdx mice. Mol. Ther. Nucleic Acids *30*, 606–620. https://doi.org/10.1016/j.omtn.2022.11.017.

77. Krishna, S., Spaulding, H.R., Koltes, J.E., Quindry, J.C., Valentine, R.J., and Selsby, J.T. (2023). Indicators of increased ER stress and UPR in aged D2-mdx and human dystrophic skeletal muscles. Front. Physiol. *14*, 1152576. https://doi.org/10.3389/fphys.2023.1152576.

78. Hulmi, J.J., Hentilä, J., DeRuisseau, K.C., Oliveira, B.M., Papaioannou, K.G., Autio, R., Kujala, U.M., Ritvos, O., Kainulainen, H., Korkmaz, A., and Atalay, M. (2016). Effects of muscular dystrophy, exercise and blocking activin receptor IIB ligands on the unfolded protein response and oxidative stress. Free Radic. Biol. Med. *99*, 308–322. https://doi.org/10.1016/j.freeradbiomed.2016.08.017.

79. Xia, Q., Huang, X., Huang, J., Zheng, Y., March, M.E., Li, J., and Wei, Y. (2021). The Role of Autophagy in Skeletal Muscle Diseases. Front. Physiol. *12*, 638983. https://doi.org/10.3389/fphys.2021.638983.

80. Cacchiarelli, D., Incitti, T., Martone, J., Cesana, M., Cazzella, V., Santini, T., Sthandier, O., and Bozzoni, I. (2011). miR-31 modulates dystrophin expression: new implications for Duchenne muscular dystrophy therapy. EMBO Rep. *12*, 136–141. https://doi.org/10.1038/embor.2010.208.

81. Fiorillo, A.A., Heier, C.R., Novak, J.S., Tully, C.B., Brown, K.J., Uaesoontrachoon, K., Vila, M.C., Ngheim, P.P., Bello, L., Kornegay, J.N., et al. (2015). TNF-α-Induced microRNAs Control Dystrophin Expression in Becker Muscular Dystrophy. Cell Rep. *12*, 1678–1690. https://doi.org/10.1016/j.celrep.2015.07.066.

82. Greco, S., De Simone, M., Colussi, C., Zaccagnini, G., Fasanaro, P., Pescatori, M., Cardani, R., Perbellini, R., Isaia, E., Sale, P., et al. (2009). Common micro-RNA signature in skeletal muscle damage and regeneration induced by Duchenne muscular dystrophy and acute ischemia. FASEB J. *23*, 3335–3346. https://doi.org/10.1096/fj.08-128579.

83. Yuasa, K., Hagiwara, Y., Ando, M., Nakamura, A., Takeda, S., and Hijikata, T. (2008). MicroRNA-206 is highly expressed in newly formed muscle fibers: implications regarding potential for muscle regeneration and maturation in muscular dystrophy. Cell Struct. Funct. *33*, 163–169. https://doi.org/10.1247/csf.08022.

84. Mollard, A., Peccate, C., Forand, A., Chassagne, J., Julien, L., Meunier, P., Guesmia, Z., Marais, T., Bitoun, M., Piétri-Rouxel, F., et al. (2022). Muscle regeneration affects Adeno Associated Virus 1 mediated transgene transcription. Sci. Rep. *12*, 9674. https://doi.org/10.1038/s41598-022-13405-9.

85. Dupont, J.-B., Tournaire, B., Georger, C., Marolleau, B., Jeanson-Leh, L., Ledevin, M., Lindenbaum, P., Lecomte, E., Cogné, B., Dubreil, L., et al. (2015). Short-lived recombinant adeno-associated virus transgene expression in dystrophic muscle is associated with oxidative damage to transgene mRNA. Mol. Ther. Methods Clin. Dev. *2*, 15010. https://doi.org/10.1038/mtm.2015.10.

86. Hildyard, J.C., and Wells, D.J. (2016). Investigating Synthetic Oligonucleotide Targeting of Mir31 in Duchenne Muscular Dystrophy. PLoS Curr. *8*. ecurrents.md.99d88e72634387639707601b237467d7. https://doi.org/10.1371/currents.md.99d88e72634387639707601b237467d7.

87. Coenen-Stass, A.M.L., Wood, M.J.A., and Roberts, T.C. (2017). Biomarker Potential of Extracellular miRNAs in Duchenne Muscular Dystrophy. Trends Mol. Med. *23*, 989–1001. https://doi.org/10.1016/j.molmed.2017.09.002.

88. Finkel, R.S., Chiriboga, C.A., Vajsar, J., Day, J.W., Montes, J., De Vivo, D.C., Yamashita, M., Rigo, F., Hung, G., Schneider, E., et al. (2016). Treatment of infantile-onset spinal muscular atrophy with nusinersen: a phase 2, open-label, dose-escalation study. Lancet *388*, 3017–3026. https://doi.org/10.1016/S0140-6736(16)31408-8.

89. Thomas, S., Conway, K.M., Fapo, O., Street, N., Mathews, K.D., Mann, J.R., Romitti, P.A., Soim, A., Westfield, C., Fox, D.J., et al. (2022). Time to diagnosis of Duchenne muscular dystrophy remains unchanged: Findings from the Muscular Dystrophy Surveillance, Tracking, and Research Network, 2000-2015. Muscle Nerve *66*, 193–197. https://doi.org/10.1002/mus.27532.

90. Ke, Q., Zhao, Z.-Y., Mendell, J.R., Baker, M., Wiley, V., Kwon, J.M., Alfano, L.N., Connolly, A.M., Jay, C., Polari, H., et al. (2019). Progress in treatment and newborn screening for Duchenne muscular dystrophy and spinal muscular atrophy. World J. Pediatr. *15*, 219–225. https://doi.org/10.1007/s12519-019-00242-6.

91. Sarepta Therapeutics Announces Voluntary Pause of ELEVIDYS Shipments in the U.S. | Sarepta Therapeutics, Inc. https://investorrelations.sarepta.com/news-releases/news-release-details/sarepta-therapeutics-announces-voluntary-pause-elevidys.

92. Peccate, C., Mollard, A., Le Hir, M., Julien, L., McClorey, G., Jarmin, S., Le Heron, A., Dickson, G., Benkhelifa-Ziyyat, S., Piétri-Rouxel, F., et al. (2016). Antisense pre-treatment increases gene therapy efficacy in dystrophic muscles. Hum. Mol. Genet. *25*, 3555–3563. https://doi.org/10.1093/hmg/ddw201.

93. Ewels, P., Magnusson, M., Lundin, S., and Käller, M. (2016). MultiQC: summarize analysis results for multiple tools and samples in a single report. Bioinformatics *32*, 3047–3048. https://doi.org/10.1093/bioinformatics/btw354.

94. Kim, D., Langmead, B., and Salzberg, S.L. (2015). HISAT: a fast spliced aligner with low memory requirements. Nat. Methods *12*, 357–360. https://doi.org/10.1038/nmeth.3317.

95. Li, H., Handsaker, B., Wysoker, A., Fennell, T., Ruan, J., Homer, N., Marth, G., Abecasis, G., and Durbin, R.; 1000 Genome Project Data Processing Subgroup (2009). The Sequence Alignment/Map format and SAMtools. Bioinformatics *25*, 2078–2079. https://doi.org/10.1093/bioinformatics/btp352.

96. Zerbino, D.R., Achuthan, P., Akanni, W., Amode, M.R., Barrell, D., Bhai, J., Billis, K., Cummins, C., Gall, A., Girón, C.G., et al. (2018). Ensembl 2018. Nucleic Acids Res. *46*, D754–D761. https://doi.org/10.1093/nar/gkx1098.

97. Raudvere, U., Kolberg, L., Kuzmin, I., Arak, T., Adler, P., Peterson, H., and Vilo, J. (2019). g:Profiler: a web server for functional enrichment analysis and conversions of gene lists (2019 update). Nucleic Acids Res. *47*, W191–W198. https://doi.org/10.1093/nar/gkz369.

98. Supek, F., Bošnjak, M., Škunca, N., and Šmuc, T. (2011). REVIGO summarizes and visualizes long lists of gene ontology terms. PLoS One *6*, e21800. https://doi.org/10.1371/journal.pone.0021800.

99. Burki, U., Keane, J., Blain, A., O'Donovan, L., Gait, M.J., Laval, S.H., and Straub, V. (2015). Development and Application of an Ultrasensitive Hybridization-Based ELISA Method for the Determination of Peptide-Conjugated Phosphorodiamidate Morpholino Oligonucleotides. Nucleic Acid Ther. *25*, 275–284. https://doi.org/10.1089/nat.2014.0528.

100. Pfaffl, M.W. (2001). A new mathematical model for relative quantification in real-time RT–PCR. Nucleic Acids Res. *29*, e45. https://doi.org/10.1093/nar/29.9.e45.

OMTN, Volume *37*

Supplemental information

Timing matters: Exon skipping therapy is most effective when initiated early in a mouse model of Duchenne muscular dystrophy

Sofia Stenler, Junyu Huang, Tirsa L.E. van Westering, Anna M.L. Coenen-Stass, Yahya Jad, Kaarel Krjutškov, Samir EL Andaloussi, Matthew J.A. Wood, and Thomas C. Roberts

Supplemental Information

Table S1

RT-qPCR and RT-ddPCR assays used in this study.

All sequence are 5′ to 3′.

		Sequence
***Dmd* exon 20-21**	**FWD**	AGATGACAACTACTGCCGAA
	**REV**	GAAGAGCTGACAATCTGTTGAC
	**PROBE**	AGTCTACCACCCTATCAGAGCCAACA
***Dmd* exon 23-24**	**FWD**	GAAACTTTCCTCCCAGTTGGT
	**REV**	CAGGCCATTCCTCTTTCAGG
	**PROBE**	TCAACTTCAGCCATCCATTTCTGTAAGGT
***Dmd* exon 1-2**	**FWD**	AGCCTCCTGGAGCAATAAAA
	**REV**	AATTGTGCATTTATCCATTTTGTGA
***Dmd* exon 44-45**	**FWD**	GCGATTCGACAGATCAGTTG
	**REV**	CCTTTCTGCCAGCTCTTTGC
***Dmd* exon 63-64**	**FWD**	CCACGAGACCCAAACCACT
	**REV**	GGGCCTTCTGGAGCCTTCT

Table S2

List of Small RNA TaqMan RT-qPCR assays used in this study.

All assays were obtained from Thermo Fisher Scientific.

Target	Product ID
mmu-miR-16-5p	000391
mmu-miR-31-5p	000185
mmu-miR-34c-5p	000428
mmu-miR-206-3p	000510

Table S3

Summary of RNA-Seq counts data and differential expression analysis.

Spreadsheet containing DESeq2 outputs including normalized counts data and pairwise differential expression analyses.

A

		Total RNA				rRNA minus
	Sample ID	ng/µl	A260/A280	A260/A230	RIN	ng/µl
Adult (14 weeks)	C57 1	77.2	2.11	1.85	9.6	16
	C57 2	72.4	2.09	1.79	8.4	10
	C57 3	63.9	2.08	1.77	9.2	9
	mdx 1	154.3	2.07	1.97	7.8	8.8
	mdx 2	65.7	2.03	1.71	6.7	8.7
	mdx 3	84.7	2.08	1.73	7.6	15
	PPMO 1	73.3	2.06	1.79	9.4	11
	PPMO 2	76	2.05	1.62	9.2	13
	PPMO 3	78.3	2.07	1.79	9.2	9.8
Aged (78-80 weeks)	C57 1	62.2	2.09	1.77	9.2	15
	C57 2	45.4	2.01	1.58	8.9	12
	C57 3	46.9	2.07	1.71	8.7	7.4
	mdx 1	70.5	2.06	1.77	8.1	11
	mdx 2	61	2.08	1.82	7.9	14
	mdx 3	51.9	2.07	1.72	8.4	14
	PPMO 1	60.8	2.03	1.53	8.7	12
	PPMO 2	46.5	2.01	1.63	8.4	7.5
	PPMO 3	77.5	2.06	1.71	9	4.8

B

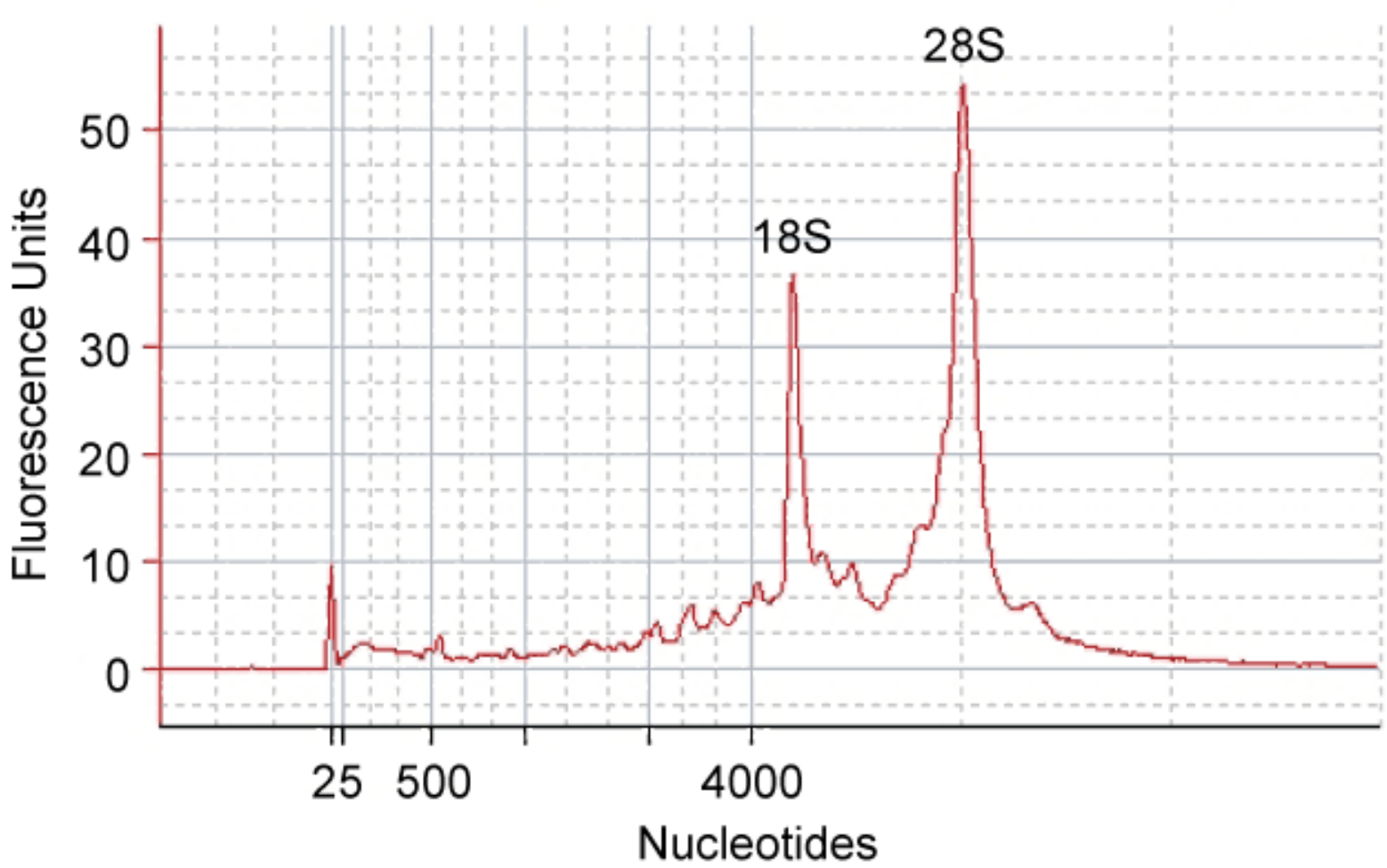

C

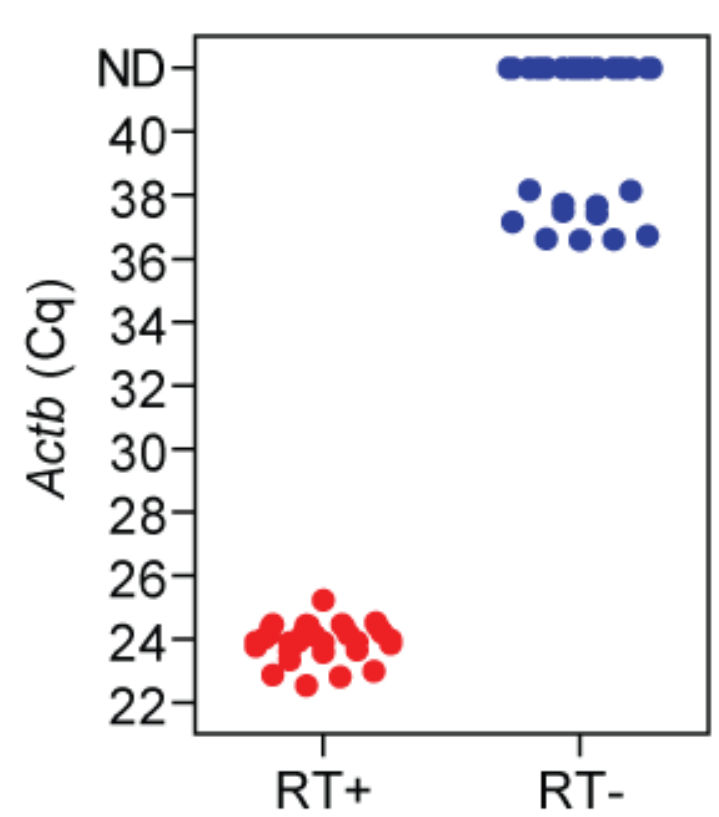

Figure S1

RNA-Seq quality control analyses.

(**A**) Summary of sample assessments of RNA concentration, quality, and integrity by UV spectrophotometry, bioanalyzer, and fluorimetry (post rRNA depletion). (**B**) Typical bioanalyzer trace indicating intact RNA samples with prominent rRNA peaks (pre rRNA removal). (**C**) RNA samples were assessed by genomic DNA contamination by measurement of *Actb* mRNA expression by RT-qPCR. This assay is designed in such a way that it has the potential to amplify both mRNA and its encoding DNA. Amplification in a RT- control sample would therefore be indicative of genomic DNA contamination. *Actb* levels in the RT- samples were either note detected (ND) or in the single copy range (Ct >35).

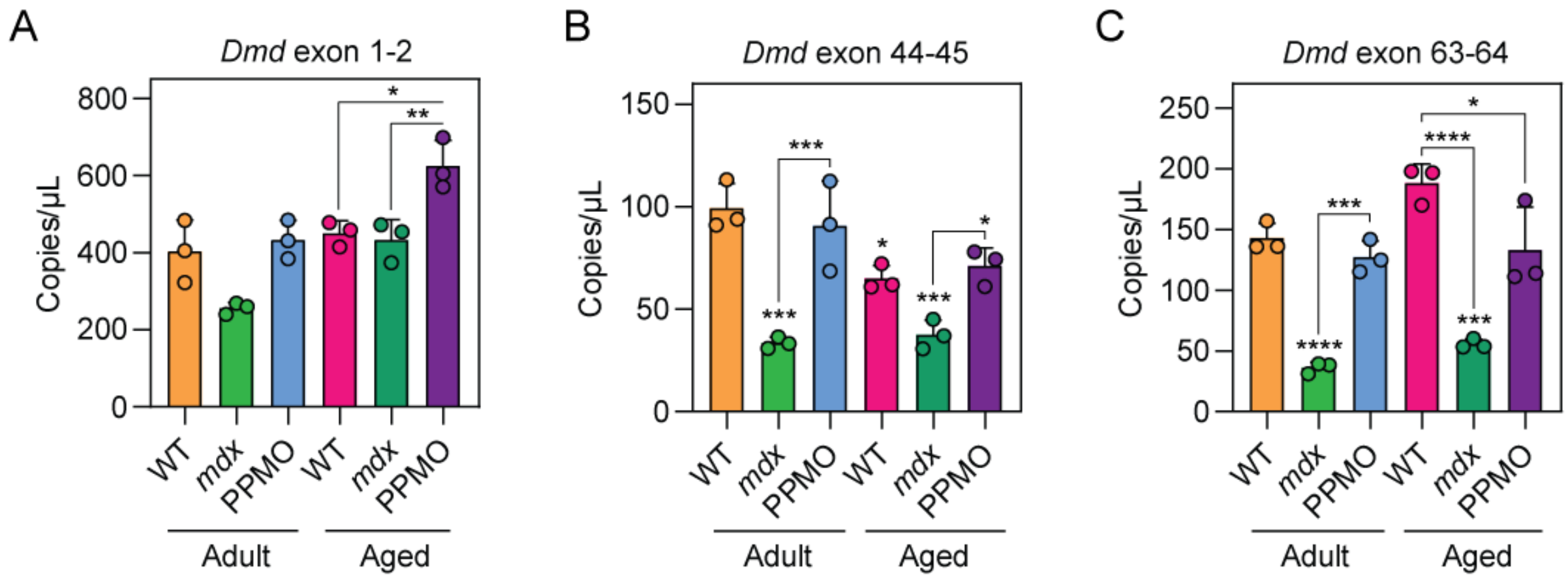

Figure S2

Analysis of *Dmd* transcript imbalance phenomena in Adult and Aged *mdx* mice.

RNA samples from WT, *mdx*, and PPMO-treated *mdx* mice at Adult and Aged time points were analysed by RT-ddPCR to determine absolute quantification of *Dmd* transcripts targeting regions (**A**) exon 1-2 (**B**) exon 44-45, and (**C**) exon 63-63. All values are mean+SD (*n*=3). Statistical significance was tested by one-way ANOVA with Tukey's *post hoc test*. Statistical comparisons are relative to the Adult WT group unless otherwise indicated, *P<0.05, **P<0.01, ***P<0.001, ****P<0.0001.

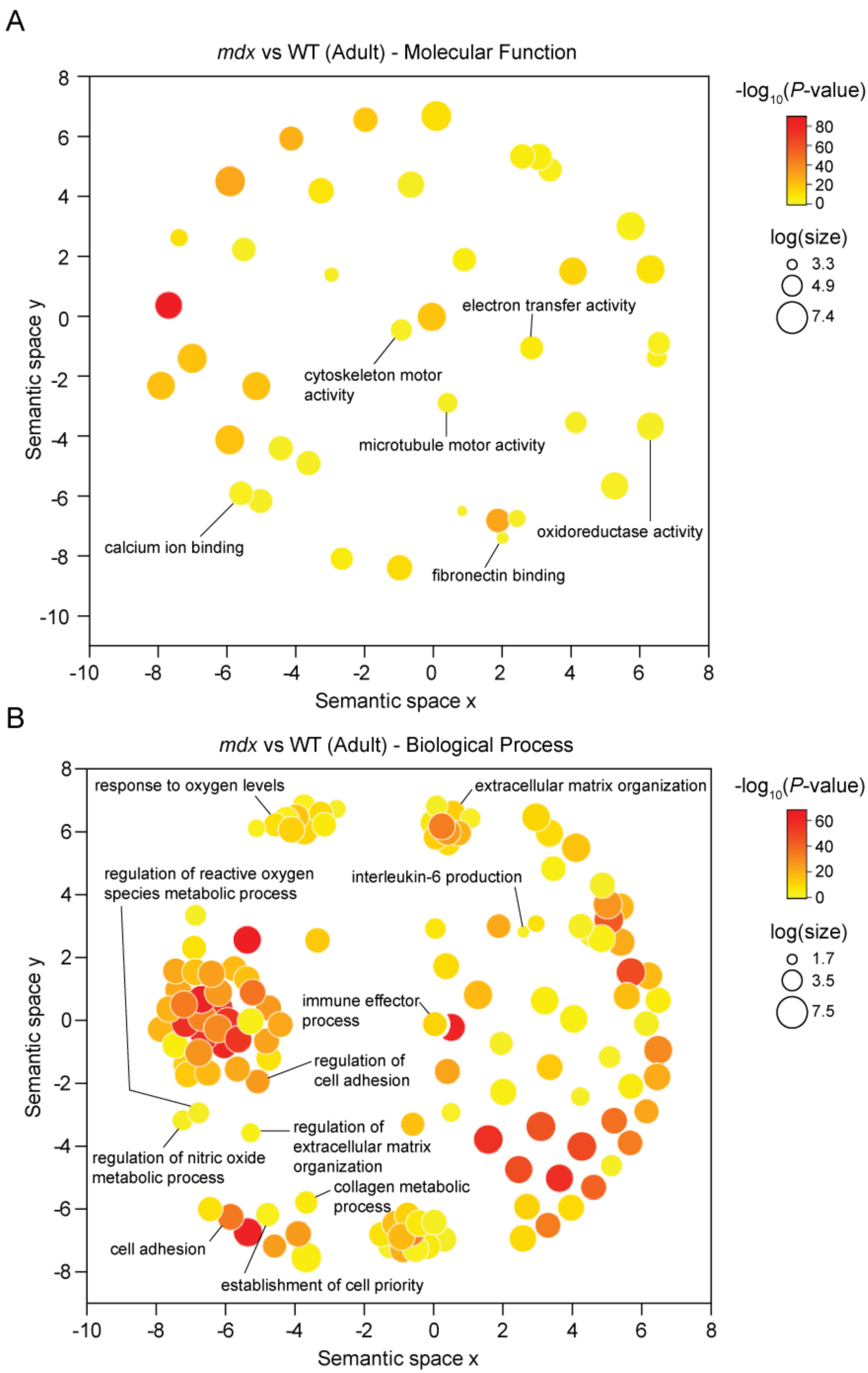
A
mdx vs WT (Adult) - Molecular Function
-log10(P-value)
80
60
40
20
0
log(size)
3.3
4.9
7.4
electron transfer activity
cytoskeleton motor activity
microtubule motor activity
calcium ion binding
oxidoreductase activity
fibronectin binding
Semantic space y
Semantic space x
B
mdx vs WT (Adult) - Biological Process
response to oxygen levels
extracellular matrix organization
regulation of reactive oxygen species metabolic process
interleukin-6 production
immune effector process
regulation of cell adhesion
regulation of extracellular matrix organization
regulation of nitric oxide metabolic process
collagen metabolic process
cell adhesion
establishment of cell priority
-log10(P-value)
60
40
20
0
log(size)
1.7
3.5
7.5
Semantic space y
Semantic space x

Figure S3

Gene ontology analysis for differentially expressed genes in Adult *mdx* vs WT.

Differentially-expressed genes from RNA-Seq data (adjusted $P<0.01$) for the Adult *mdx* vs WT libraries were analysed for enriched gene ontology (GO) terms using g:Profiler and the statistically significant (adjusted $P<0.05$) terms visualized using REVIGO to reduce redundancy and improve interpretability for (**A**) molecular function, and (**B**) biological process. Circle size indicates the frequency of the gene ontology annotation term in the underlying database, and colour indicates the statistical significance of the enrichment. Distances between circles reflect semantic similarity among GO terms.

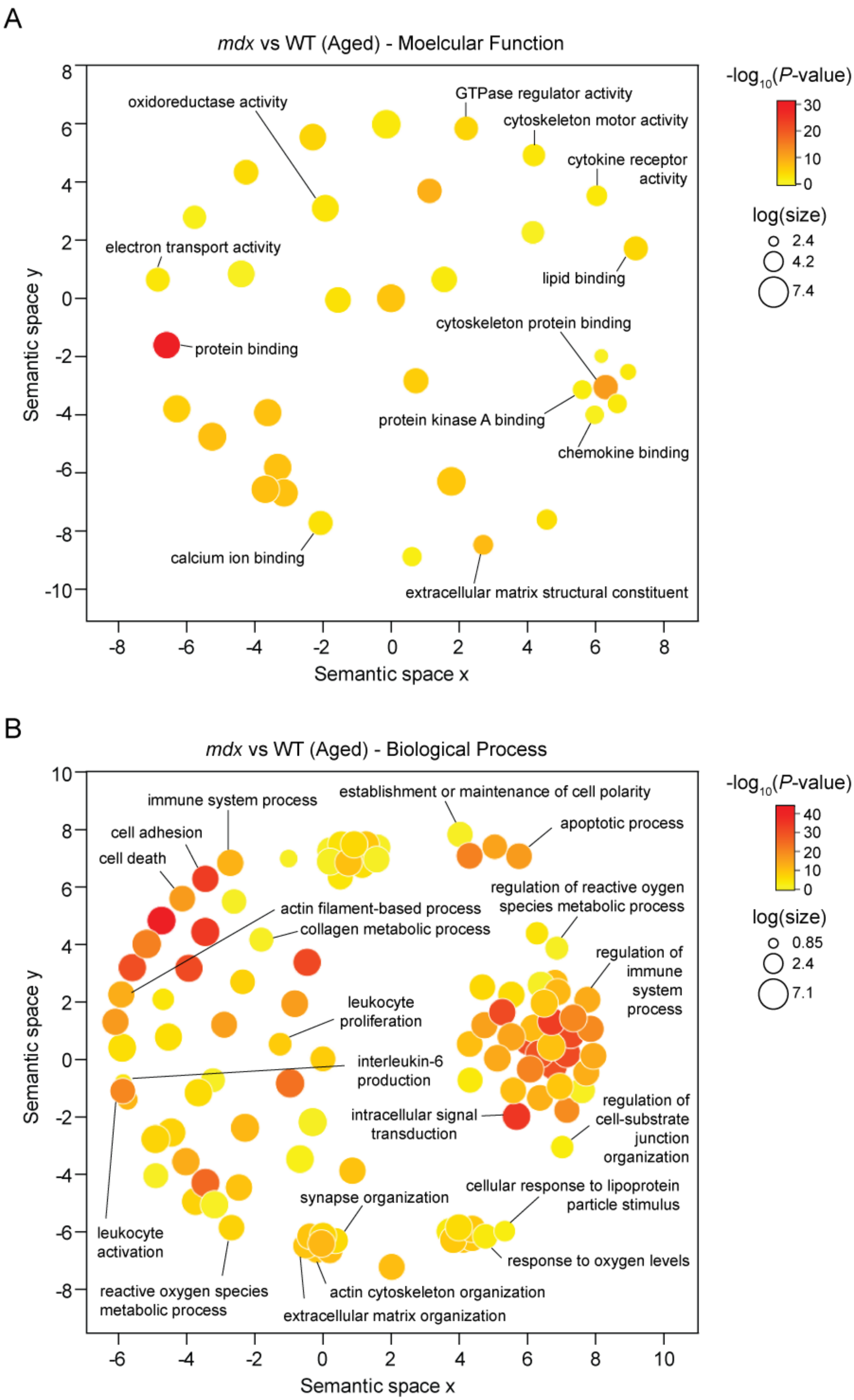

A
mdx vs WT (Aged) - Moelcular Function
oxidoreductase activity
GTPase regulator activity
cytoskeleton motor activity
cytokine receptor activity
electron transport activity
lipid binding
cytoskeleton protein binding
protein binding
protein kinase A binding
chemokine binding
calcium ion binding
extracellular matrix structural constituent
Semantic space y
Semantic space x
-log10(P-value)
30
20
10
0
log(size)
2.4
4.2
7.4
8
6
4
2
0
-2
-4
-6
-8
-10
-8
-6
-4
-2
0
2
4
6
8
B
mdx vs WT (Aged) - Biological Process
immune system process
establishment or maintenance of cell polarity
cell adhesion
apoptotic process
cell death
regulation of reactive oygen species metabolic process
actin filament-based process
collagen metabolic process
regulation of immune system process
leukocyte proliferation
interleukin-6 production
intracellular signal transduction
regulation of cell-substrate junction organization
cellular response to lipoprotein particle stimulus
synapse organization
leukocyte activation
response to oxygen levels
reactive oxygen species metabolic process
actin cytoskeleton organization
extracellular matrix organization
Semantic space y
Semantic space x
-log10(P-value)
40
30
20
10
0
log(size)
0.85
2.4
7.1
10
8
6
4
2
0
-2
-4
-6
-8
-6
-4
-2
0
2
4
6
8
10

Figure S4

Gene ontology analysis for differentially expressed genes in Aged *mdx* vs WT.

Differentially-expressed genes from RNA-Seq data (adjusted $P<0.01$) for the Aged *mdx* vs WT libraries were analysed for enriched gene ontology (GO) terms using g:Profiler and the statistically significant (adjusted $P<0.05$) terms visualized using REVIGO to reduce redundancy and improve interpretability for (**A**) molecular function, and (**B**) biological process. Circle size indicates the frequency of the gene ontology annotation term in the underlying database, and colour indicates the statistical significance of the enrichment. Distances between circles reflect semantic similarity among GO terms.

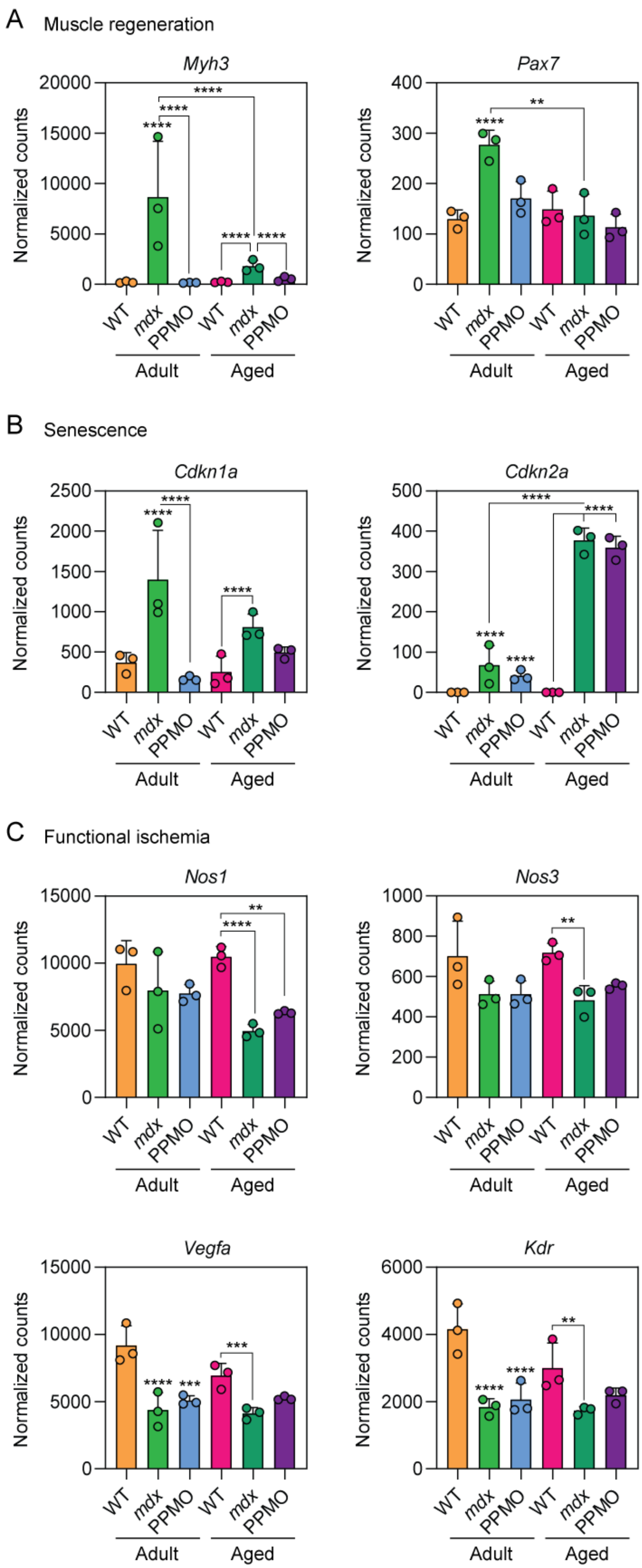

A
Muscle regeneration
Myh3
Pax7
Normalized counts
20000
15000
10000
5000
0
400
300
200
100
****
**
WT
mdx
PPMO
Adult
Aged
B
Senescence
Cdkn1a
Cdkn2a
2500
2000
1500
1000
500
C
Functional ischemia
Nos1
Nos3
1000
800
600
400
200
***
Vegfa
Kdr
6000
4000
2000

Figure S5

Differential expression of muscle regeneration, senescence, and functional ischemia markers in dystrophic muscle.

RNA-Seq normalized counts data for marker genes associated with (**A**) muscle regeneration, (**B**) senescence, and (**C**) functional ischemia. Values are mean+SD. Statistical significance was assessed using DESeq2 and Benjamini-Hochberg-adjusted *P*-values indicated. Statistical comparisons are relative to the Adult WT group unless otherwise indicated, $^{**}P<0.01$, $^{***}P<0.001$, $^{****}P<0.0001$.

Handling of reactive oxygen species

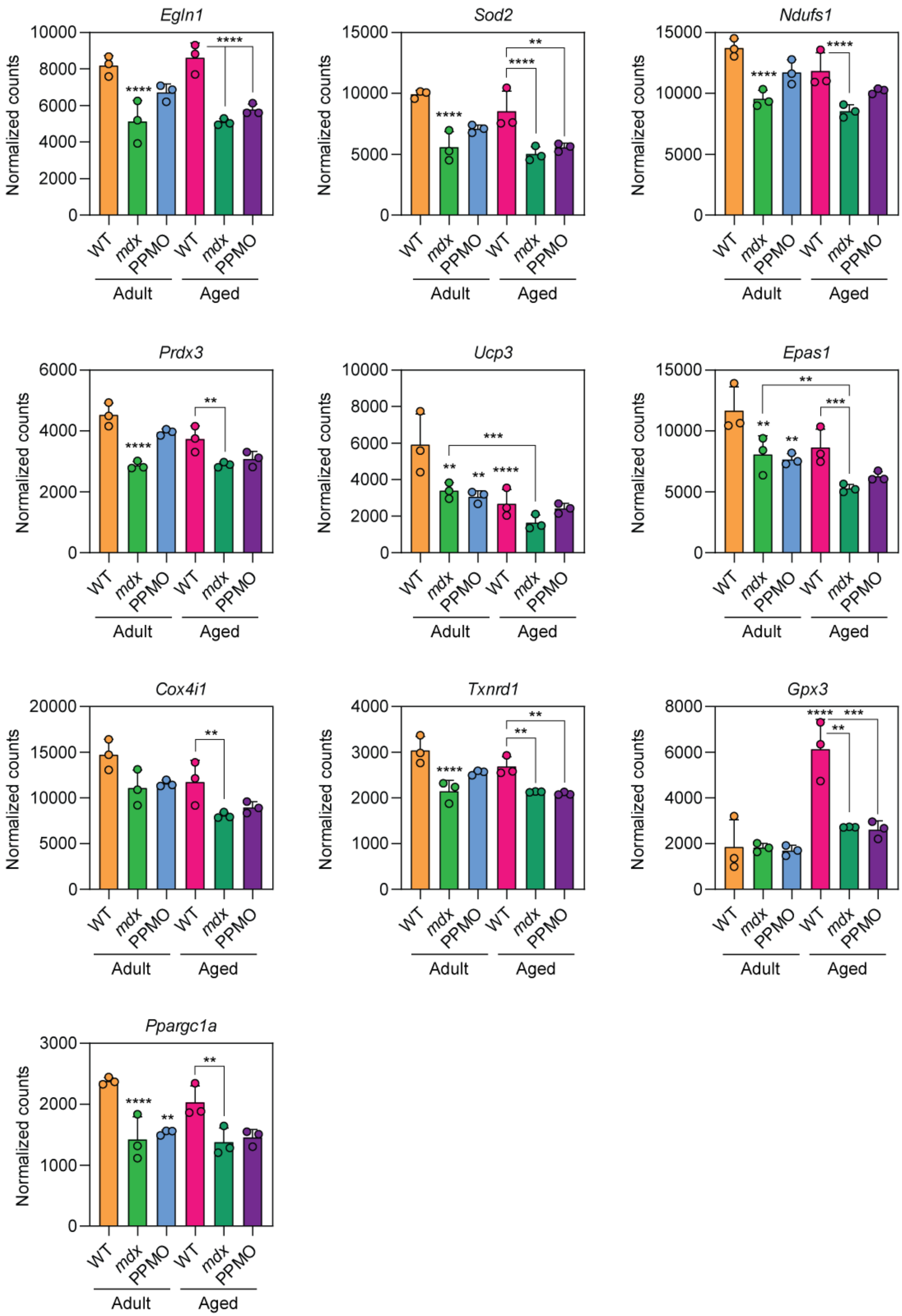

Figure S6

Differential expression of reactive oxygen species handling markers in dystrophic muscle.

RNA-Seq normalized counts data for marker genes associated with handling of reactive oxygen species. Values are mean+SD. Statistical significance was assessed using DESeq2 and Benjamini-Hochberg-adjusted *P*-values indicated. Statistical comparisons are relative to the Adult WT group unless otherwise indicated, **$P<0.01$, ***$P<0.001$, ****$P<0.0001$.

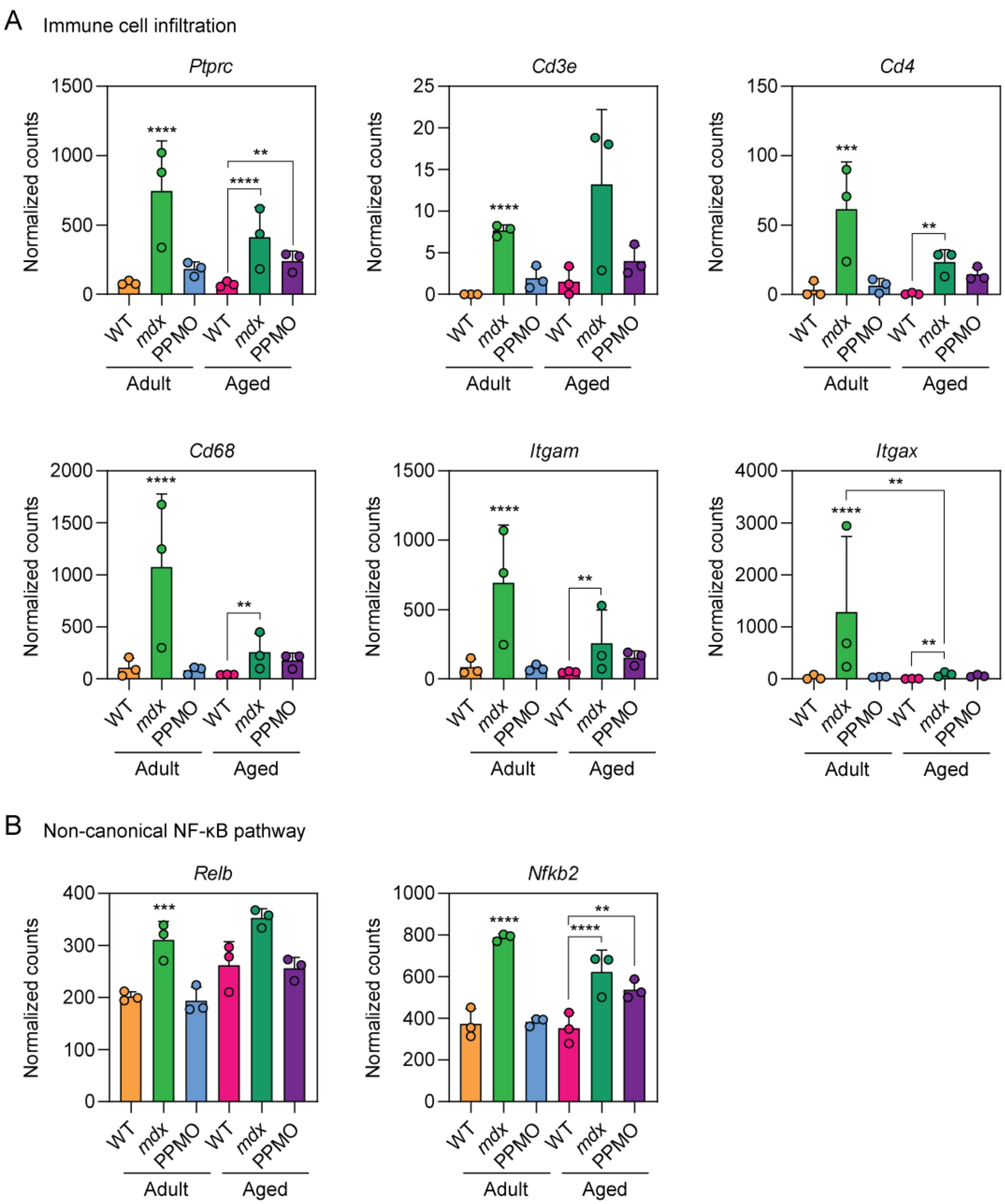
A
Immune cell infiltration
Ptprc
Cd3e
Cd4
Cd68
Itgam
Itgax
Normalized counts
WT
mdx
PPMO
Adult
Aged
B
Non-canonical NF-κB pathway
Relb
Nfkb2

Figure S7

Differential expression of immune infiltration and NF-κB pathway markers in dystrophic muscle.

RNA-Seq normalized counts data for marker genes associated with (**A**) immune cell infiltration and (**B**) the non-canonical NF-κB pathway. Values are mean+SD. Statistical significance was assessed using DESeq2 and Benjamini-Hochberg-adjusted *P*-values indicated. Statistical comparisons are relative to the Adult WT group unless otherwise indicated, **$P<0.01$, ***$P<0.001$, ****$P<0.0001$.

A Fibrosis

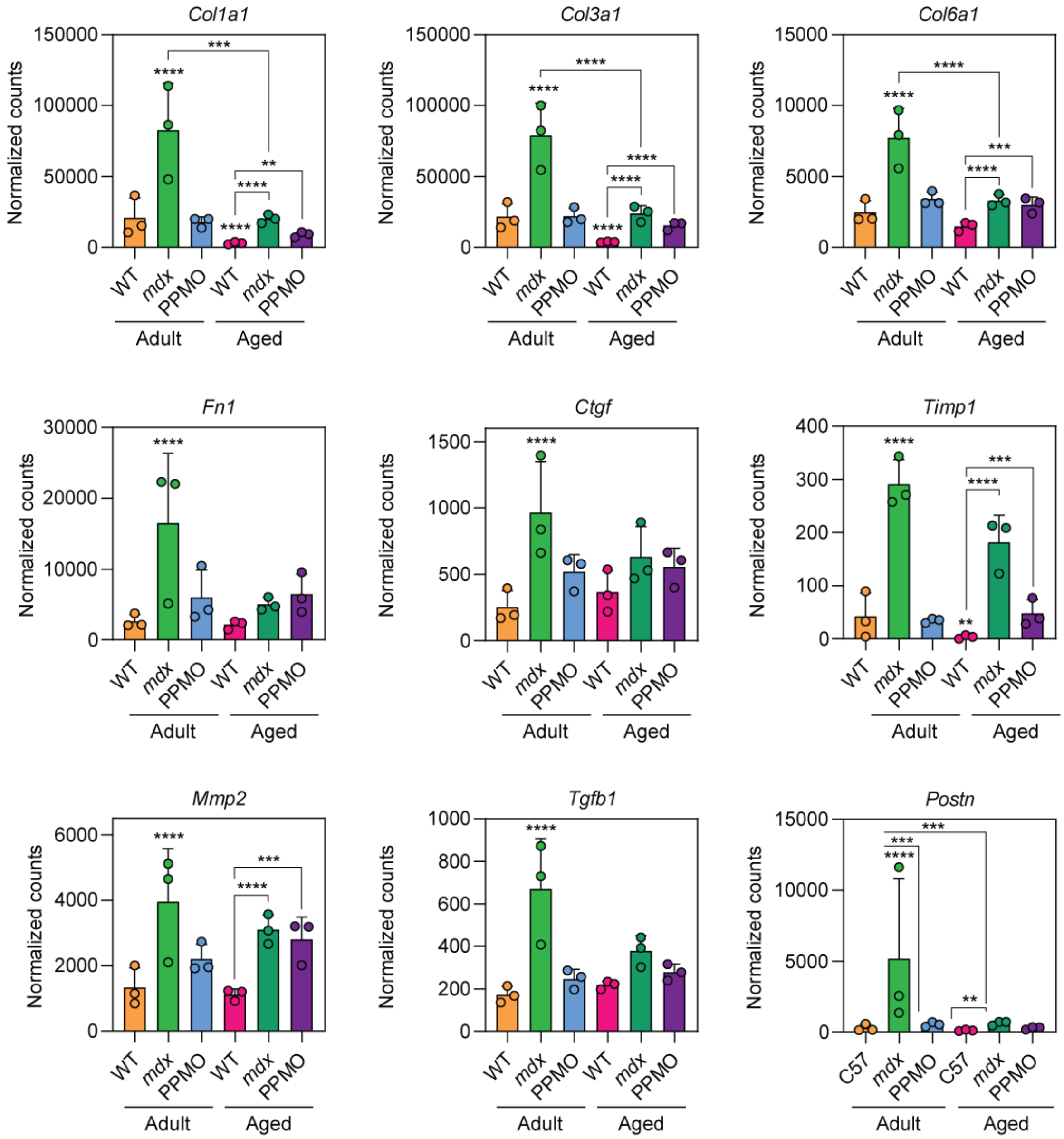

B Fibroadipo progenitor (FAP) markers

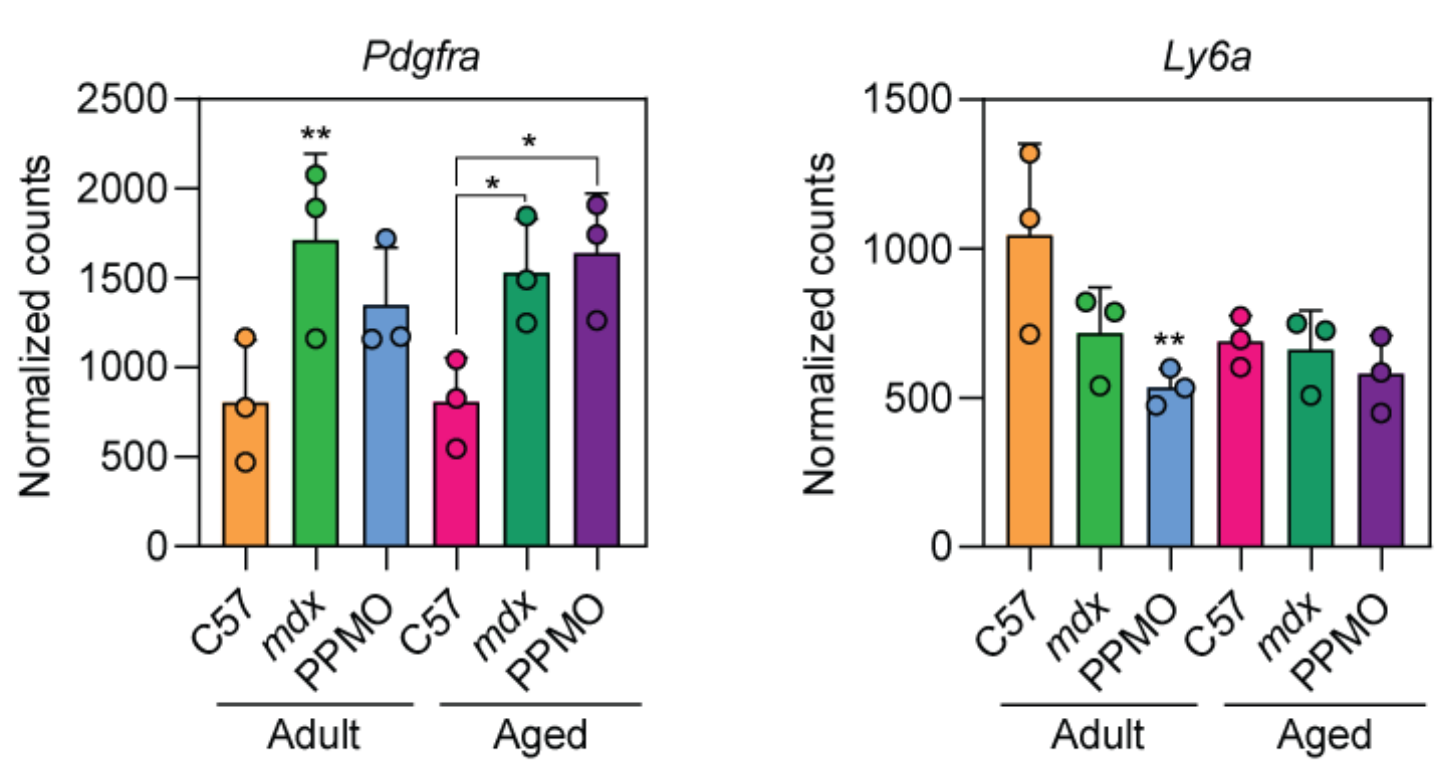

Figure S8

Differential expression of fibrosis markers in dystrophic muscle.

RNA-Seq normalized counts data for marker genes associated with (**A**) fibrosis, and (**B**) fibroadipo progenitor (FAP) markers. Values are mean+SD. Statistical significance was assessed using DESeq2 and Benjamini-Hochberg-adjusted *P*-values indicated. Statistical comparisons are relative to the Adult WT group unless otherwise indicated, $^{*}P<0.05$, $^{**}P<0.01$, $^{***}P<0.001$, $^{****}P<0.0001$.

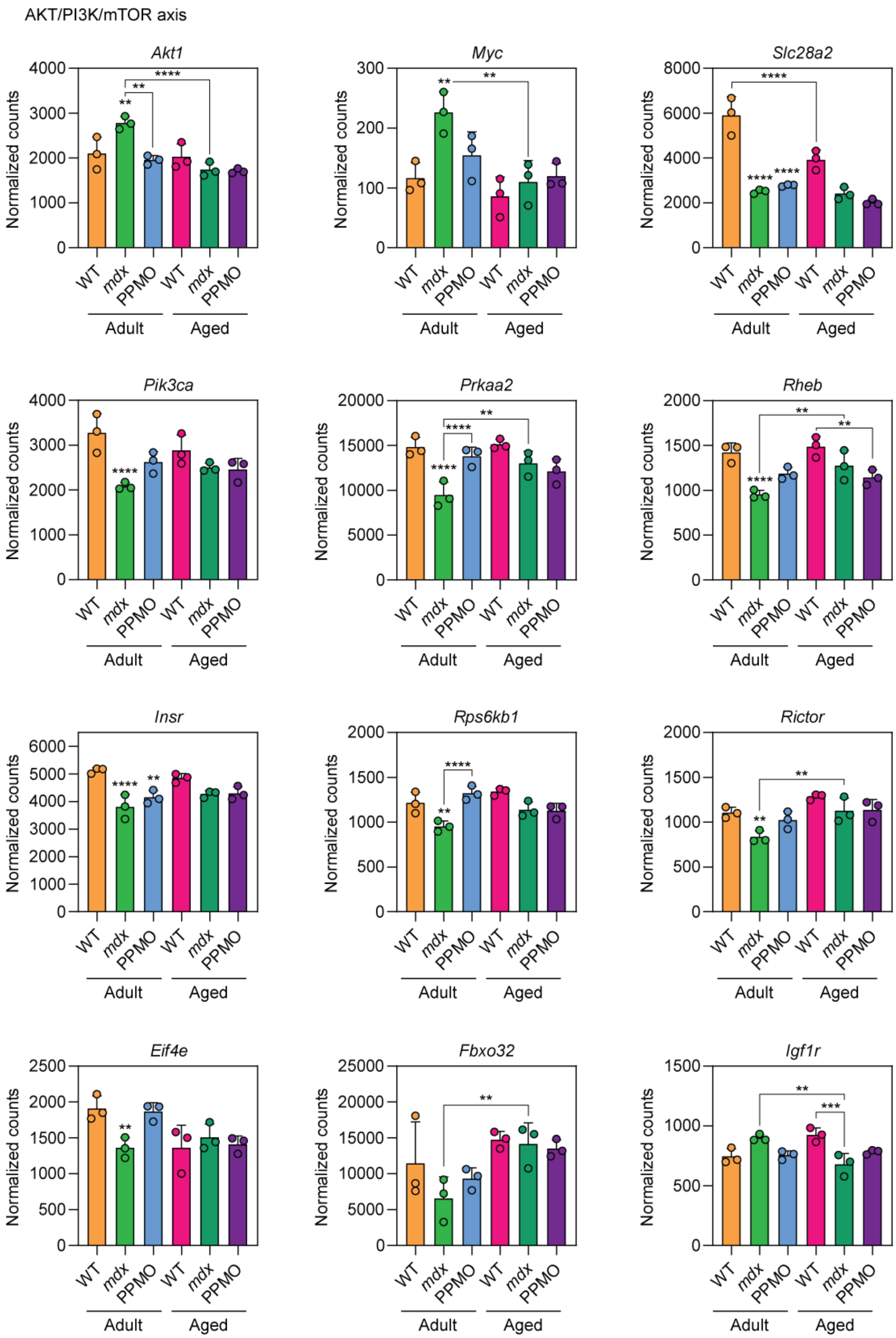
AKT/PI3K/mTOR axis
Akt1
Myc
Slc28a2
Pik3ca
Prkaa2
Rheb
Insr
Rps6kb1
Rictor
Eif4e
Fbxo32
Igf1r
Normalized counts
WT
mdx
PPMO
Adult
Aged

Figure S9

Differential expression of AKT/PI3K/mTOR axis markers in dystrophic muscle.

RNA-Seq normalized counts data for marker genes associated with the AKT/PI3K/mTOR axis. Values are mean+SD. Statistical significance was assessed using DESeq2 and Benjamini-Hochberg-adjusted *P*-values indicated. Statistical comparisons are relative to the Adult WT group unless otherwise indicated, $^{**}P<0.01$, $^{***}P<0.001$, $^{****}P<0.0001$.

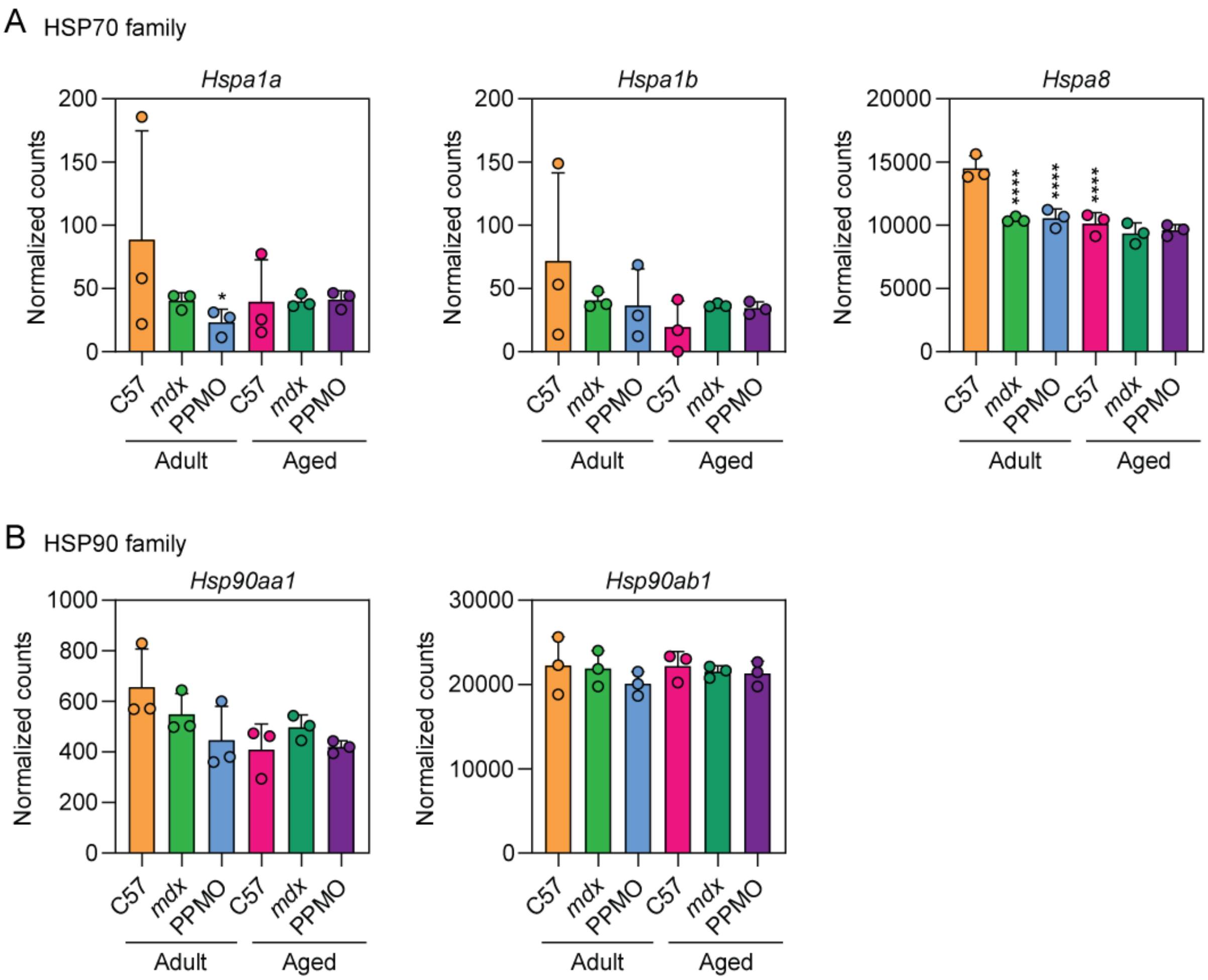
A
HSP70 family
Hspa1a
Hspa1b
Hspa8
Normalized counts
200
150
100
50
0
20000
15000
10000
5000
*
****
C57
mdx
PPMO
Adult
Aged
B
HSP90 family
Hsp90aa1
Hsp90ab1
1000
800
600
400
200
30000
20000
10000

Figure S10

Differential expression of molecular chaperones in dystrophic muscle.

RNA-Seq normalized counts data for marker genes associated with molecular chaperons from the (**A**) HSP70 family, and (**B**) HSP90 family. Values are mean+SD. Statistical significance was assessed using DESeq2 and Benjamini-Hochberg-adjusted *P*-values indicated. Statistical comparisons are relative to the Adult WT group unless otherwise indicated, $^{**}P<0.01$, $^{****}P<0.0001$.

Ubiquitin-proteasome system

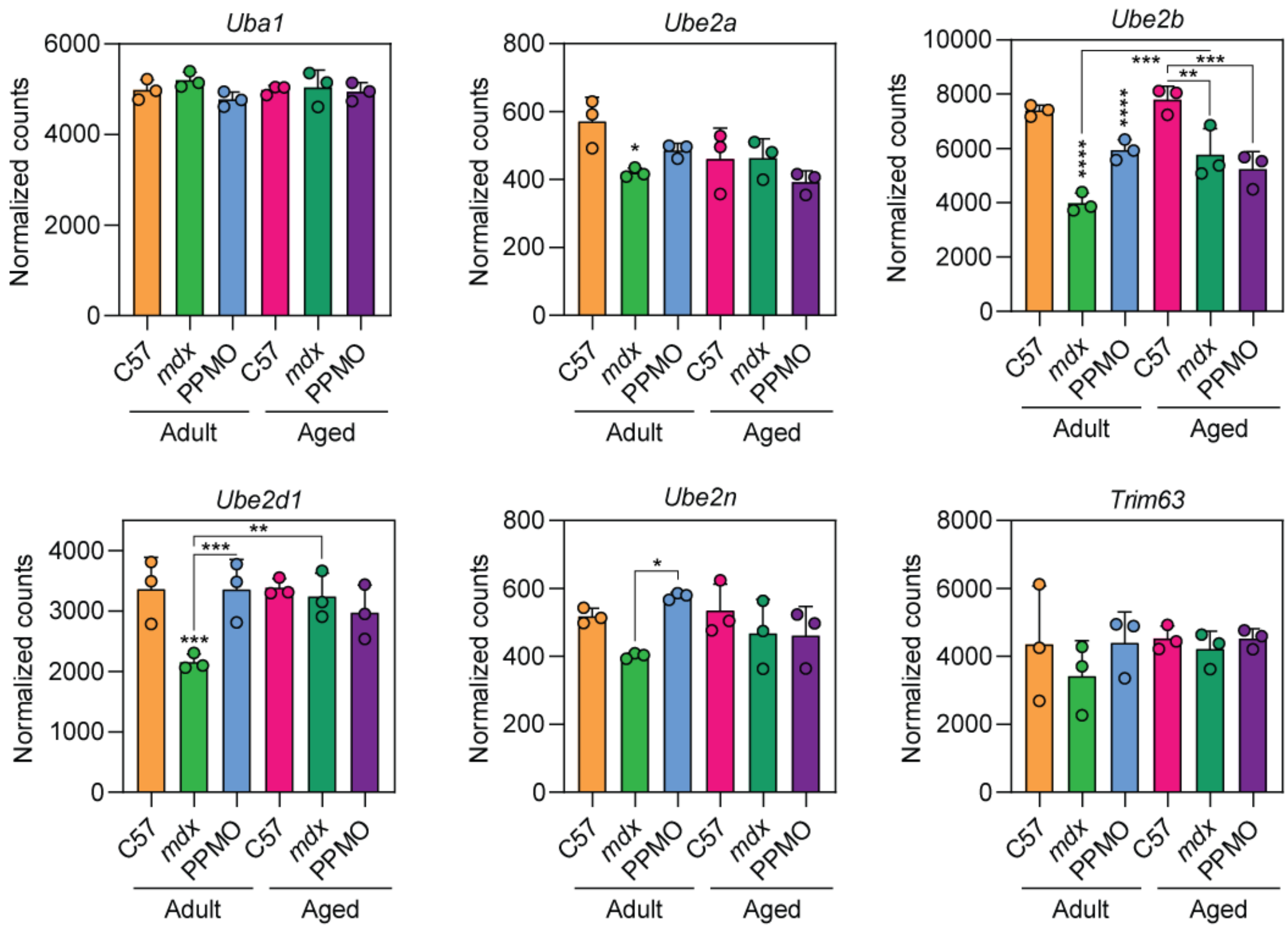

Figure S11

Differential expression of components of the ubiquitin-proteasome system in dystrophic muscle.

RNA-Seq normalized counts data for marker genes associated with components of the ubiquitin-proteasome system. Values are mean+SD. Statistical significance was assessed using DESeq2 and Benjamini-Hochberg-adjusted *P*-values indicated. Statistical comparisons are relative to the Adult WT group unless otherwise indicated, *$P<0.05$, **$P<0.01$, ***$P<0.001$, ****$P<0.0001$.

Autophagy

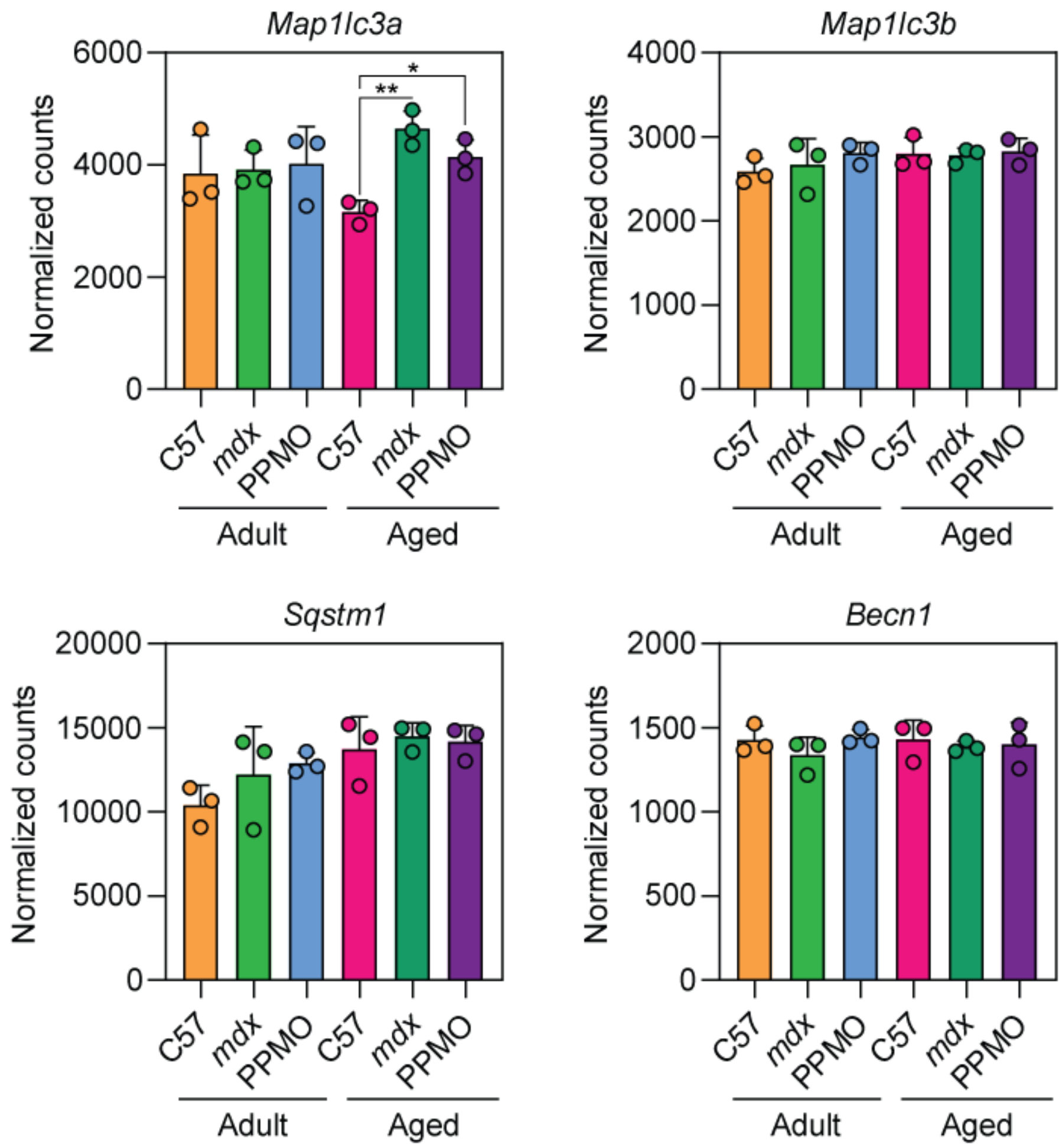

Map1lc3a
Map1lc3b
Sqstm1
Becn1
Normalized counts
6000
4000
2000
0
3000
1000
20000
15000
10000
5000
1500
500
**
*
C57
mdx
PPMO
Adult
Aged

Figure S12

Differential expression autophagy associated genes in dystrophic muscle.

RNA-Seq normalized counts data for marker genes associated with autophagy. Values are mean+SD. Statistical significance was assessed using DESeq2 and Benjamini-Hochberg-adjusted *P*-values indicated. $^*P<0.05$, $^{**}P<0.01$.
